# Incidence, timing, and clinical significance of adverse immune events after gene replacement therapy: A systematic review and meta-analysis

**DOI:** 10.1016/j.ymthe.2026.01.004

**Published:** 2026-01-10

**Authors:** Niccolò Maurizi, Enrico Ammirati, Elizabeth Silver, Kimberly Hong, Quan Bui, Alessia Argirò, Iacopo Olivotto, Eric D. Adler

**Affiliations:** 1 Cardiomyopathy Unit, Careggi University Hospital, Florence, Italy; 2 Service of Cardiology, University Hospital of Lausanne (CHUV) and University of Lausanne (Unil), Lausanne, Switzerland; 3 De Gasperis Cardio Center, Transplant Center, Niguarda Hospital, Milano, Italy; 4 Division of Cardiovascular Medicine, Department of Medicine, University of California, San Diego, San Diego, CA, USA; 5 Cardiology Unit, IRCCS Meyer’s Children Hospital, Florence, Italy

**Keywords:** gene replacement, adverse events, AAV therapy

## Abstract

Adeno-associated virus (AAV)-based gene replacement has emerged as a transformative platform for severe genetic disorders, yet immune-mediated adverse events (AEs) pose significant barriers to widespread clinical adoption. We performed a systematic review and meta-analysis of prospective and retrospective studies of AAV gene therapy published between January 2005 and March 2025 (PROSPERO CRD420251046546). Data from 801 studies encompassing 1,972 patients and 2,142 patient-years were pooled to estimate the incidence and clinical impact of immunotoxicity. Random-effects meta-analysis yielded a 30.0% (95% CI, 22.5–38.9; *I*^2^ = 83.1%) overall AE rate, including hepatotoxicity in 23.8% (17.4–31.7; *I*^2^ = 81.7%), myocarditis in 6.2% (4.6–8.1; *I*^2^ = 46%), thrombotic microangiopathy (TMA) in 4.7% (4.4–6.5; *I*^2^ = 18.5%), and treatment-related death in 4.7% (3.0–5.3; *I*^2^ = 46.1%). Hepatotoxicity and myocarditis were generally mild (97% and 96% non-serious), whereas all TMA episodes carried substantial morbidity. Time course analyses revealed TMA clustered in week 1, myocarditis at week 2, and hepatotoxicity up to 6 months post-infusion. In individual-patient analyses, vector serotype and doses >1 × 10^12^ vg/kg significantly increased AE risk (OR = 5.59 [1.35–12.2], *p* = 0.018; OR = 2.31 [1.04–5.53], *p* = 0.041), whereas combined corticosteroid, anti-CD20, mTOR- and calcineurin inhibitor regimens were protective (OR = 0.67 [0.47–0.96], *p* = 0.040). At least five cases of TMA, one of myocarditis, and three deaths could not be included in the present analysis because these events were described in company statements. These findings underscore that one-third of AAV recipients experience immunotoxicity, predominantly early and mild, and support proactive immunosuppression and vector optimization to enhance safety.

## Graphical abstract

**Figure:**
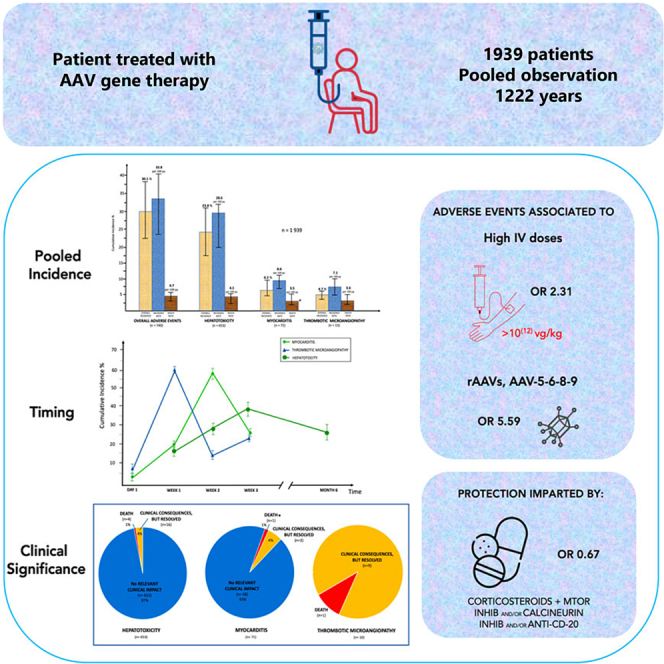

## Teaser

Meta-analysis of 1,939 patients in systemic AAV trials found immune-mediated adverse events in 23%, with hepatotoxicity being the most common, while myocarditis and thrombotic microangiopathy remain less frequent but warrant vigilant monitoring due to potential severity. These results emphasize early post-infusion surveillance and a need for better understanding of immunomodulation strategies.

## Introduction

Gene therapy using adeno-associated viral (AAV) vectors has emerged as a transformative approach for treating a range of severe genetic disorders.^1^^,^^2^^,^^3^ However, the prospect of widespread clinical adoption has been tempered by concerns over immune activation and related adverse events (AEs) such as hepatotoxicity, myocarditis, and thrombotic microangiopathy.^4^^,^^5^^,^^6^^,^^7^ To date, our understanding of these complications has been derived exclusively from individual trial reports and small case-series or industry communications, leaving the incidence and clinical impact of immune-mediated AEs largely undefined.^8^ Furthermore, because genetic therapies are often trialed in small numbers of patients, AEs at low or even medium frequencies may not be detected until more patients are treated. Characterizing the immunological responses associated with various gene therapy treatments would be crucial for optimizing treatment protocols, ensuring better safety and efficacy. Despite multiple studies documenting isolated incidents of AAV-associated immunotoxicity,^9^ there has not been a comprehensive analysis that quantifies the overall incidence, characterizes clinical significance, or identifies factors associated with AEs. Therefore, this systematic review and meta-analysis aims to address this critical knowledge gap by synthetizing and characterizing AAV-based gene therapy AEs described in published studies and records from the International Pharmacovigilance Databases.

## Results

**Figure 1:**
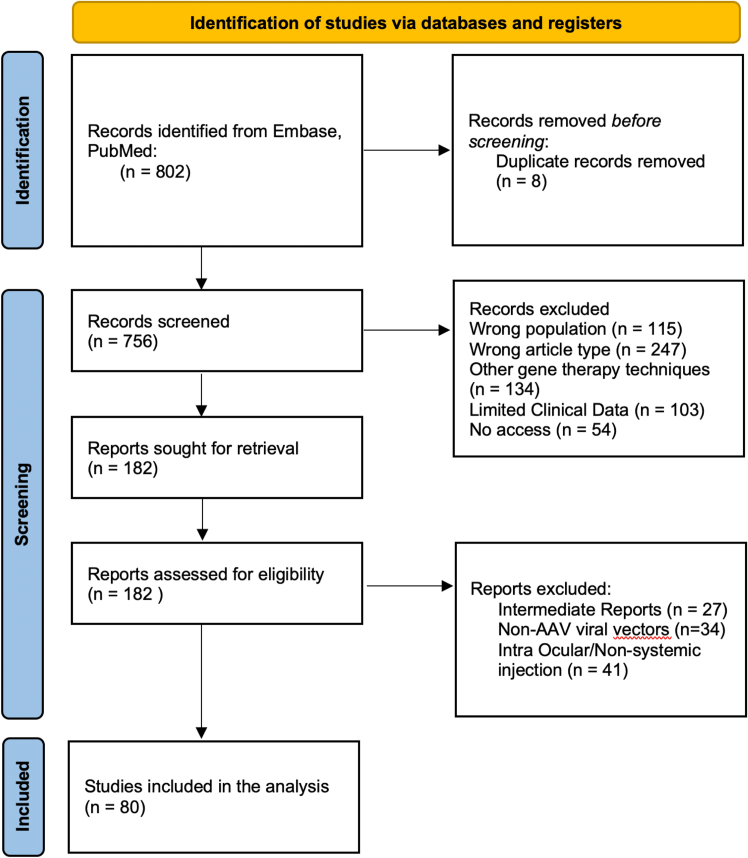
Preferred reporting items for systematic reviews and meta-analyses selection and analysis process of the reports included in the systematic review and meta-analysis

A total of 756 abstracts were screened and 182 full texts have been reviewed, resulting in 81 studies comprising 1,972 patients^23^^,^^24^^,^^25^^,^^26^^,^^27^^,^^28^^,^^29^^,^^30^^,^^31^^,^^32^^,^^33^^,^^34^^,^^35^^,^^36^^,^^37^^,^^38^^,^^39^^,^^40^^,^^41^^,^^42^^,^^43^^,^^44^^,^^45^^,^^46^^,^^47^^,^^48^^,^^49^^,^^50^^,^^51^^,^^52^^,^^53^^,^^54^^,^^55^^,^^56^^,^^57^^,^^58^^,^^59^^,^^60^^,^^61^^,^^62^^,^^63^^,^^64^^,^^65^ (Figure 1). Details about included studies, treated disease, type of vector used, dose, and immunosuppressive regimen can be found in Tables 1 and S4. The majority of included studies were clinical trials (61, 76%), whereas 19 (24%) were observational studies. Most of the studies included <50 patients (70 [88%] studies) and dose ranged from 3.3 × 10^8^ to 1.1 × 10^14^ vg/kg. Three types of immunosuppressive regimen were used: reactive corticosteroids (investigator administration of corticosteroids following a possible AE) (28 [35%] studies, 915 patients), pre- and post-injection corticosteroid therapy (42 [53%] studies, 968 patients), and pre- and post-injection corticosteroid therapy and/or mTOR inhibitors and/or calcineurin inhibitor and/or anti-CD-20 monoclonal antibodies (9 [12%] studies, 56 patients) (Table 1). One study did not report the immunosuppression regimen.^24^ A total of 35/81 studies involved patients treated for spinal muscular atrophy (SMA) and Duchenne muscular dystrophy (DMD).

**Table 1:** Overview of the studies included by the target disease

Disease treated	No. of studies	Sample size			Vector used	Doses	Immunosuppressive regimen		
		<10	10–50	>50			Reactive corticosteroids	Pre- + post-treatment corticosteroids	Pre- + post-treatment corticosteroids + mTOR inhib and/or calcineurin inhib and/or anti-CD-20
Danon^23^	1	1	0	0	AAV-9	6.7 × 10^13^ to 1.1 × 10^14^ vg/kg	0	0	1
Anderson-Fabry^24^	1	1	0	0	AAV-2	1 × 10^13^ to 5 × 10^13^ vg/kg	0	1	0
Duchenne muscular dystrophy^25^^,^^26^^,^^27^^,^^28^^,^^29^^,^^30^^,^^31^^,^^32^^,^^33^^,^^34^^,^^35^	11	8	2	1	AAV-8, AAV-9, rAAVrh74	1 × 10^12^ to 1 × 10^14^ vg/kg	1	6	2
Limb-girdle muscular dystrophy 2B^36^^,^^37^	2	2	0	0	rAAV-1, rAAVrh74	1 × 10^11^ to 7.4 × 10^13^ vg/kg	1	1	0
Pompe^38^^,^^39^^,^^40^	3	3	0	0	rAAV, AAV-8, AAV-1	1 × 10^12^ to 5 × 10^12^ vg/kg	1	1	1
Spinal muscular atrophy^41^^,^^42^^,^^43^^,^^44^^,^^45^^,^^46^^,^^47^^,^^48^^,^^49^^,^^50^^,^^51^^,^^52^^,^^53^^,^^54^^,^^55^^,^^56^^,^^57^^,^^58^^,^^59^^,^^60^^,^^61^^,^^62^^,^^63^^,^^64^	24	8	13	3	scAAV9-FL-SMNcDNA	6.7 × 10^13^ to 1.1 × 10^14^ vg/kg	0	24	0
X-linked myotubular myopathy^65^	1	0	1	0	AAV-8	1.3 × 10^14^ to 3.5 × 10^14^ vg/kg	0	1	0
Hemophilia A^66^^,^^67^^,^^68^^,^^69^^,^^70^^,^^71^^,^^72^	7	1	4	2	AAV-3, AAV-5, rAAV-6	9 × 10^11^ to 6 × 10^13^ vg/kg	5	2	0
Hemophilia B^73^^,^^74^^,^^75^^,^^76^^,^^77^^,^^78^^,^^79^	7	2	3	2	rAAV-2, AAV-5, AAV-8, AAV-2, AAVs3	8 × 10^10^ to 2 × 10^13^ vg/kg	5	2	0
AADC deficiency^80^	1	0	1	0	AAV-2	1.8 × 10^11^ to 2.4 × 10^13^ vg/kg	1	0	0
Autosomal recessive deafness^81^	1	1	0	0	AAV-1	9 × 10^11^ to 1.5 × 10^12^ vg/kg	1	0	0
Frontotemporal dementia^82^	1	0	1	0	AAV-9	2.1 × 10^13^ to 4.2 × 10^13^ vg/kg	0	0	1
Crigler-Najar^83^	1	1	0	0	AAV-8	2 × 10^12^ to 5 × 10^12^ vg/kg	0	0	1
Mucoplysaccharidosis type IIIA/IIB/IV^84^^,^^85^^,^^86^^,^^87^	4	4	0	0	AAV-2/5, AAV-8, rAAV	6 × 10^11^ to 6 × 10^12^ vg/kg	0	1	3
Becker muscular dystrophy^88^	1	1	0	0	AAV-1	3 × 10^11^ to 6 × 10^13^ vg/kg	0	1	0
Chronic heart failure^89^^,^^90^^,^^91^^,^^92^^,^^93^	5	2	2	1	AAV-1, Ad5-FDG	1.4 × 10^11^ to 1 × 10^13^ vg/kg	5	0	0
Stable angina pectoris^94^^,^^95^^,^^96^	4	0	4	0	Ad5-FDG	3.3 × 10^8^ to 1 × 10^11^ vg/kg	4	0	0
Acute intermittent porphyria^97^	1	1	0	0	rAAV-2/5	5 × 10^11^ to 1.8 × 10^13^ vg/kg	1	0	0
Lipoprotein lipase deficiency^98^	1	1	0	0	AAV-1	1 × 10^12^ to 5 × 10^12^ vg/kg	0	1	0
Glycogen storage disease type Ia^99^	1	0	1	0	AAV-2	2 × 10^12^ to 6 × 10^13^ vg/kg	1	1	0
Tay-Sachs^100^	1	1	0	0	rAAVrh8	1 × 10^13^ vg/Kg	0	1	0
HIV^101^	1	0	1	0	AVV-1	1 × 10^12^ to 1 × 10^14^ vg/kg	1	0	0
AAT deficiency^102^	1	0	1	0	rAAV, AAV-2	0.5 × 10^13^ to 5 × 10^13^ vg/kg	0	1	0

AAV, adenovirus; AAT, α-1 antitrypsin.

The risk for bias assessments showed that, for clinical trials, four (5%) were at moderate risk of bias for outcome measure, four (5%) at unclear risk for selective reporting, with an overall risk of moderate bias of 3%. For observational studies, four (17%) presented some concerns in the patient selection, four (17%) in confounding, with an overall risk of bias of 8% (Tables S5 and S6; Figure S1); funnel plots are presented in Figure S2. Missing data per study are reported in Table S7. A random-effects meta-regression on the logit-transformed incidence proportions formally tested that the incidence of AEs did not differ between clinical trials and observational studies (β = −0.580, OR = 0.51 [0.24–1.12], *p* = 0.081) (Table S5).

### Pooled incidence of immune-mediated AEs

A total of 734 AEs were reported over 2,152 patient-years of pooled observation. Hepatotoxicity occurred in 653 patients, followed by myocarditis in 71 and TMA in 10. Six treatment-related deaths have been reported. A total of 31 (39%) studies did not report any AE, whereas 9 (11%) presented an AE in more than 80% of the patients included (Figure S3).

**Figure 2:**
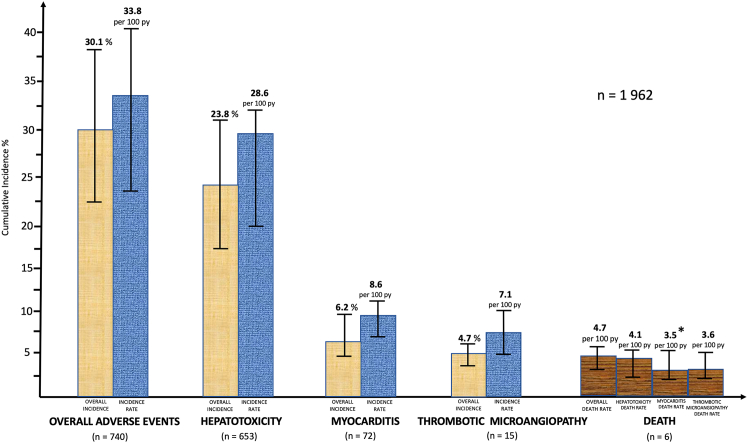
Pooled and annual incidence of immune adverse events and death after AAV gene replacement therapy Pooled incidence of adverse events, hepatotoxicity, myocarditis, and thrombotic microangiopathy cases are presented with their 95% confidence intervals. py, patient-years. ∗Episode of death occurred in the context of a cytokine-mediated capillary leak syndrome with an associated cardiac dysfunction due to treatment acute toxic effect on a background of a pre-existing cardiomyopathy. Concomitant myocarditis was possible, but did not probably represented the direct cause of death..

Pooled incidences were, respectively: 30% (95% CI, 22.5%–38.9%; *I*^2^ = 83.1%, *p* < 0.01) for all AEs, 23.8% (95% CI, 17.4%–31.7%; *I*^2^ = 81.7%, *p* < 0.01) for the occurrence of hepatotoxicity, 6.2% (95% CI, 4.6%–8.1%; *I*^2^ = 46%, *p* = 0.07) for myocardial injury/myocarditis, 4.73% (95% CI, 4.4%–6.5%; *I*^2^ = 18.5%, *p* = 0.083) for TMA and 4.7% (95% CI, 3%–5.3%; *I*^2^ = 46.1%, *p* = 0.809) for treatment-related death (Figure 2). Pooled incidence rates per 100 patient-years were: 33.8 (95% CI, 23.4–43.1%; *I*^2^ = 46.1%, *p* < 0.01) for all AEs, 28.6% (95% CI, 19.9%–36.7%; *I*^2^ = 82.6%, *p* < 0.01) for hepatotoxicity, 8.6% (95% CI, 5.8%–10.7%; *I*^2^ = 63.2%, *p* = 0.13) for myocardial injury/myocarditis, and 7.1% (95% CI, 4.7%–10.7%; *I*^2^ = 52.8%, *p* = 0.065) for TMA (Figure 2).

### Timing and clinical significance of immune-mediated Aes

**Figure 3:**
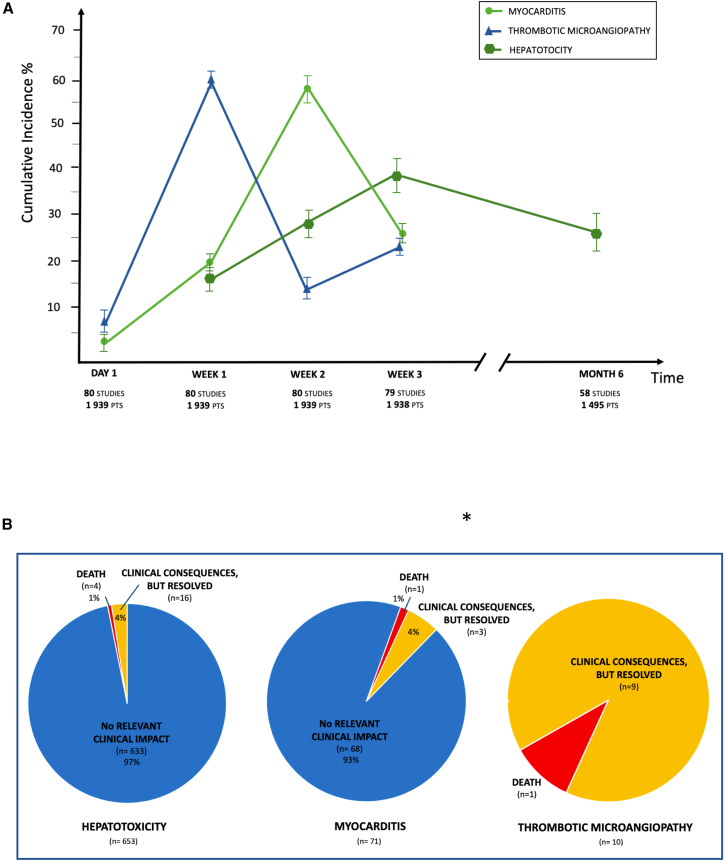
Timing of onset and clinical impact of gene replacement-related myocarditis, thrombotic microangiopathy, and hepatitis (A) The timing of onset of myocarditis, hepatitis, and thrombotic microangiopathy is presented. For each time point, the cumulative incidence is displayed, with the 95% confidence intervals. (B) The clinical significance for adverse events is shown: from no relevant clinical impact, with clinical consequences but resolved in the follow-up or if it resulted in death. ∗Episode of death occurred in the context of a cytokine-mediated capillary leak syndrome with an associated cardiac dysfunction due to treatment acute toxic effect on a background of a pre-existing cardiomyopathy. Concomitant myocarditis was possible, but did not probably represent the direct cause of death.

TMA occurred mostly at week 1 after treatment (60.2% [95% CI, 57.2%–62.9%; *I*^2^ = 47.5%]), whereas myocardial injury/myocarditis peaked at week 2 (57.9% [95% CI, 55.3%–62.4%; *I*^2^ = 16.1%]). No cases of myocardial injury/myocarditis or TMA were reported after the first month post-injection (Figure 3A). Hepatotoxicity had a median time of onset of 38 days (IQR = 19–67 days) and occurred up to 6 months after injection (24.2% [95% CI, 22.1%–28.8%; *I*^2^ = 74.1%]).

Of the 71 cases of myocarditis, in 36 (51%) patients it presented with concomitant hepatotoxicity^26^^,^^28^^,^^32^^,^^35^^,^^42^^,^^49^^,^^50^^,^^51^^,^^52^^,^^59^^,^^60^^,^^65^ (Figure S4). The clinical picture involved mild elevation of cardiac enzymes without echocardiographic changes (68/71, 94%) (Table S8). In the four cases who had clinical consequences, all but one resolved completely at the end of the study period (Figure 3B).^26^^,^^28^^,^^35^ Hepatotoxicity was the most common AE, reported in 653 patients, consisting in an asymptomatic elevation of liver function tests in 633 (97%). In 16 (4%) patients, it progressed to transient hepatic failure. TMA was rare, occurring in 10 patients, but all had serious clinical consequences requiring hospitalization (Figure 2B).

A total of six deaths have been reported.^32^^,^^46^^,^^65^ Case 1^32^ was a 27-year-old male with DMD treated with rAAV-9 using a dead *Staphylococcus aureus* Cas9 at high dose. The patient had restrictive pulmonary defect, severe reduced muscle mass with severe muscle wasting, and a compensated cardiomyopathy. He experienced a cytokine-mediated capillary leak syndrome with consequent cardiac dysfunction, related to an acute effect of the AAV gene therapy. Concomitant myocarditis was possible, but was likely not the primary cause of death. Case 2^46^ occurred in a 4-month-old patient affected by SMA, treated with high dose scAAV-9, carrying a variant in the complement factor 1 gene (probably partially responsible for the severity of the reaction) who developed the first week after injection a severe clinical picture of TMA leading to multi-organ failure day 30 after injection. The remaining four cases^65^ were reported in patients from 2 to 6 years old treated with AAV-8 gene replacement for X-linked myotubular myopathy (XLMTM) due to liver failure. Although baseline transaminases and bilirubin were within protocol limits, subsequent clinical and histopathologic evaluations in several XLMTM patients revealed pre-existing hepatobiliary structural abnormalities that likely increased susceptibility to AAV-associated hepatotoxicity^65^ (Table S9).

### Factors associated with occurrence of immune-mediated AEs

Hepatotoxicity occurred only in patients with hematologic, neurologic, or hepatic diseases, whereas myocardial injury/myocarditis and TMA were reported only in patients with muscular and cardiac diseases (Figure S6).

**Figure 4:**
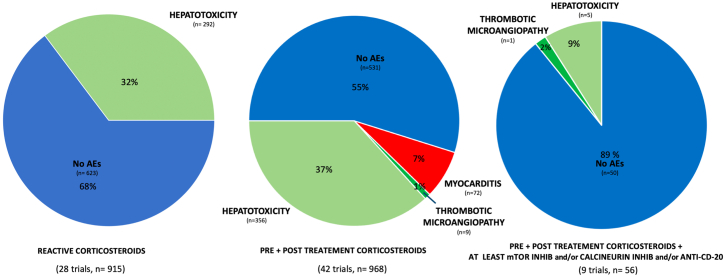
Incidence of adverse events based on the immunosuppressive regimen Inhib, inhibitors.

AAV-5, AAV-6, AVV-2/8 and AAV-8, AAV-9, and recombinant AAVs increased the risk of AEs (OR = 5.59 [1.35–12.2], *p* = 0.018 and OR = 3.96 [1.01–12.2], *p* = 0.04), as well as a dose >10^12^ vg/kg (OR = 2.31 [1.04–5.53], *p* = 0.04) (Table 2). Aggressive immunosuppressive regimen (pre- or post-treatment corticosteroids with mTOR inhibitor and/or calcineurin inhibitors and/or anti-CD-20) showed protection against AEs (OR = 0.67 [0.07–0.96], *p* = 0.04) (Figure 4).

**Table 2:** Predictors of adverse to immune activation after gene replacement therapy

Variable	Category	Immune-mediated adverse events (*n* = 734)	OR (95% CI)	*p* values
Vector class	high pre-existing population immunogenicity (Ad5-FGF5; AAV-1; AAV-2/5)	14/367 (4%)	–	–
	intermediate pre-existing population immunogenicity (AAV-5; AAV-6)	361/678 (53%)	5.59 [1.35–12.2]	0.018
	low pre-existing population immunogenicity (AAV-8; AAV-9, recombinants AAV)	373/894 (42%)	3.96 [1.01–9.7]	0.04
Dose	≤10 (12) vg/kg	91/495 (18%)	–	–
	10 (13) to 10 (14) vg/kg	648/1 444 (45%)	2.31 (1.04–5.53)	0.041
Immunosuppression	reactive corticosteroids only	292/915 (32%)	–	–
	pre- + post-treatment corticosteroids	437/968 (45%)	0.8 (0.6–1.49)	0.164
	pre- + post-treatment corticosteroids + mTOR inhib and/or calcineurin inhib and/or anti-cd-20	6/56 (14%)	0.67 (0.07–0.96)	0.04

For each variable, a clinically relevant comparison group have been chosen. For vector class, for doses the odds ratio of 10^13^ to 10^14^ vg/kg with respect to ≤10^12^ vg/kg are provided. For immunosuppression regimen, reactive corticosteroids are compared with more intense immunosuppressive regimens and the odds ratios are presented.

AAV, adenovirus; OR, odds ratio; mTOR Inhib, mammalian target of rapamycin inhibitors.

### Global pharmacovigilance analysis

A total of 2,134 and 2,197 AEs have been reported in VigiAccess and FDA Adverse Event Reporting System (FAERS) Databases, respectively, for 5 commercially available gene replacement drugs (Table 3). Immune-mediated AEs were 262/2,134 (12%) and 448/2,197 (20%). Hepatotoxicity was the most common reported AEs for all four drugs in all databases. Myocarditis occurred in relation to delandistrogene moxeparvovec (1/16 [6%] in VigiAccess, 11/29 [38%] in FAERDS) and onasemnogene abeparvovec (14/217 [6%] in VigiAccess, 118/395 [30%] in FAERDS, although for the latter it is not known whether a baseline increase of the troponin was present).

**Table 3:** Real-world immune adverse events as reported in the VigiAccess and FAERS Database in commercially available gene replacement treatments

		Delandistrogene moxeparvovec	Onasemnogene abeparvovec	Etranacogene dezaparvovec	Valoctocogene roxaparvovec	Eladocagene exuparvovec
Vector		rAAVeh74	AAV-9	AAV-5	AAV-5	AAV2-hRPE65v2
Disease		DMD	SMA	Hem B	Hem A	AADC deficiency
Patients treated		≅800	≅4,000	N/A	N/A	N/A
Overall reported AEs	VigiAccess	101	1,969	23	20	21
	FAERS	122	2,012	22	34	6
Total immune-mediated AEs	VigiAccess	16/101 (16%)	217/1,969 (11%)	18/23 (78%)	11/20 (55%)	0
	FAERS	29/122 (24%)	395/2,012 (20%)	5/22 (23%)	19/34 (56%)	0
Hepatotoxicity	VigiAccess	15/16 (94%)	160/217 (74%)	18 (100%)	11 (100%)	0
	FAERS	18/29 (62%)	277/395 (70%)	5 (100%)	19 (100%)	0
Myocarditis	VigiAccess	1/16 (6%)	14/217 (6%)	0	0	0
	FAERS	11/29 (38%)	118/395 (30%)	0	0	0
Thrombotic microangiopathy	VigiAccess	0	43/217 (21%)	0	0	0
	FAERS	0	0	0	0	0

AEs, adverse events; DMD, Duchenne muscular dystrophy; FAERS, FDA Adverse Event Reporting System.

TMA cases (43/217, 20%) were reported only in VigiAccess in relation to onasemnogene abeparvovec. No AEs were present for eldocagene exuparvovec (Table 3).

## Discussion

The widespread clinical adoption of gene replacement therapy has been tempered by concerns over immune activation.^5^^,^^6^^,^^7^ To date, the understanding of these complications has been limited and derived exclusively from individual trial reports, small case-series, or company communications.^8^ We report here the first comprehensive analysis to our knowledge of the incidence and clinical significance of immune-mediated AEs after gene replacement therapy. We analyzed 80 studies encompassing 1,939 patients treated over a total of 2,122 patient-years. Overall, one-third of patients experienced at least one immune-mediated AE (pooled incidence 30%; 95% CI, 22.5%–38.9%; *I*^2^ = 83.1%). Hepatotoxicity was the most prevalent complication (pooled incidence 23.8%), followed by myocarditis (6.2%) and TMA (pooled incidence 4.7%). Time-to-onset differed by AE type: TMA generally emerged within the first week, myocarditis peaked in week 2, and hepatotoxicity could manifest up to 6 months post-infusion. While AAV-mediated immunotoxicity is relatively common, most events cluster in the early post-treatment period and are transient or mild in severity.^103^

### Timing and clinical significance of immune-mediated AEs

Clinically, the majority of AEs were mild and required minimal intervention. Of 653 hepatotoxicity events, 97% were asymptomatic liver function test elevations; only 16 cases (4%) progressed to transient hepatic failure, resolved by study end. Importantly, several of the most severe hepatobiliary events occurred in the context of pre-existing hepatobiliary vulnerability (histopathologic or developmental biliary anomalies), not always apparent on routine baseline transaminase testing.^64^ Thus, while transaminase elevations were common, they were not uniformly predictive of liver failure. A major limitation of the current literature is inconsistent reporting of liver-synthetic and cholestatic indices. These parameters were not available in a standardized fashion across studies and therefore could not be systematically analyzed. Finally, although the majority of hepatotoxic events clustered within the first 4–12 weeks, a minority of reports described new liver abnormalities up to 6 months after dosing. Potential explanations for delayed presentations include protracted immune activation, evolving cholestatic processes in predisposed individuals, drug interactions unmasking subclinical disease, or delayed recovery from earlier subclinical injury.^7^

Myocarditis and myocardial injury was reported in 71 patients, mostly (96%) presenting with mild troponin elevations without left ventricular dysfunction or wall motion abnormalities.^42^^,^^49^^,^^50^^,^^51^^,^^52^^,^^59^^,^^60^^,^^65^ Four required brief hospitalization and all but one fully recovered.^32^ Early (week 1–2) troponin elevations, often with systemic TMA or complement features are compatible with innate and complement-driven endothelial injury (C3a/C5a-mediated activation), whereas later events are more consistent with adaptive, cytotoxic T cell responses against the capsid or transgene product. Complement cleavage products (C3a, C5a), which are potent endothelial activators and implicated in AAV-associated TMA, might be useful in monitoring algorithms to differentiate early innate events from delayed cellular myocarditis.^4^ No cases of myocarditis/myocardial injury or TMA were reported beyond 1 month post-infusion. In contrast, TMA, although infrequent (*n* = 10), was uniformly severe, necessitating hospitalization and advanced supporting care. Six deaths related to AAV therapy were documented. These fatal cases were characterized by high vector doses, underlying organ-specific vulnerability (e.g., pre-existing liver disease), and early onset (<8 weeks), suggesting that patient selection (e.g., baseline organ function) and more conservative dosing may be crucial in selected cases for preventing lethal outcomes.

We recommend systematic reporting of AE with rigorous scientific studies. At least five cases of TMA, one of myocarditis, and three deaths could not be included in the present analysis because these events were described in company statements.^104^^,^^105^^,^^106^^,^^107^^,^^108^ The impact on the pooled incidence would have not been substantial, but the absence of transparent and scientifically rigorous reporting are missed opportunities to improve the understanding of the underlying pathophysiologic mechanisms and their prevention.

### Factors associated with immune-mediated AEs

Vector serotype and dose were associated with AEs. Specifically, certain serotypes conferred a more than 5-fold increase in AE risk and higher doses (>1 × 10^12^ vg/kg) doubled the risk (Figure S5), underscoring the need to balance effective transgene expression with immunological safety. Conversely, regimens incorporating both pre- and post-infusion corticosteroids and mTOR inhibitors and/or calcineurin inhibitors and/or anti-CD-20 monoclonal antibodies reduced AE risk by 33% (despite use in fewer studies, resulting in a small sample size). However, because these agents differ fundamentally in their targets, the pooled estimates should be interpreted as hypothesis-generating only. Yet, the underlying immunopathogenic pathways remain incompletely elucidated.^4^ For instance, TMA appears to be mediated by complement activation in the presence of high anti-capsid antibody titers,^6^ yet the precise sequence of endothelial injury and platelet consumption is poorly characterized. There is a critical need for mechanistic studies, both in preclinical models and in early-phase trials, to systematically evaluate cytokine profiles, complement activation markers, endothelial injury assays, and histological characterization, alongside pharmacokinetic and pharmacodynamic parameters.^4^^,^^109^ Such investigations should also test whether tailored immunosuppressive regimens (e.g., complement inhibitors, transient B cell depletion) can interrupt these pathways without blunting transgene expression or excessively immunosuppressing the patient.

### Pharmacovigilance analysis

Our analysis of two major pharmacovigilance databases corroborates the toxicity profile observed in clinical trials. Immune-mediated events comprised 12%–20% of AEs reported for the five commercially approved AAV therapies. Hepatotoxicity remained the most common AE, while myocarditis was reported in association with delandistrogene moxeparvovec (6% in VigiBase; 38% in FAERS) and onasemnogene abeparvovec (6% in VigiBase; 30% in FAERS), although baseline troponin data were inconsistently documented in the latter. TMA represented 20% of all AEs for onasemnogene abeparvovec in VigiBase but was not captured in FAERS, likely reflecting underreporting or differences in case-definition adjudication. The alignment of real-world pharmacovigilance data with our pooled trial results reinforces the external validity of our findings and highlights that, even in less-controlled settings, immune-mediated AEs follow a similar distribution by organ system and severity.

### Limitations of the study

This analysis is limited by underlying study quality. Considerable proportions of studies had limited patient numbers and moderate risks for bias. By design, substantial interstudy heterogeneity exists regarding the inclusion criteria and more than 40% of studies available were analyzing patients treated for DMD and SMA. Accordingly, the analysis employed random-effects meta-analyses applying the method of Hartung, Knapp, Sidik, and Jonkman to better account for interstudy variance and conducted meta-regression to explore the heterogeneity in the magnitude of associations. Small-study effects, including publication bias, may have resulted in the overestimation of the pooled incidences, particularly for total number of AEs and hepatotoxicity. Our reliance on published aggregate data and the construction of a pseudo-individual participant dataset precludes adjustment for key patient-level confounders such as baseline disease severity and concomitant medications and may not reflect causal relationship. Moreover, variability in follow-up duration and AE surveillance windows across studies may have led to underestimation of late-onset or subclinical immunotoxicity. Pharmacovigilance database analyses are limited by passive reporting and lack of detailed clinical adjudication, which limit conclusions about real-world immunotoxicity profiles. Lastly, we acknowledge that, while drafting this analysis, two deaths occurred. The first patient, participating in the phase 2 trial of RP-A501^107^ complications related to a capillary leak syndrome and the concomitant use of a novel immune suppression agent in the pre-treatment regimen, implemented to mitigate complement activation observed in a phase I study. On May 23, 2025, the FDA placed a clinical hold on the trial.^108^ The second death occurred in a patient with DMD treated with delandistrogene moxeparvovec-rokl following complications from acute liver failure, treated with pre- and post-injection corticosteroids.^108^

### Conclusions

AAV gene therapy is associated with a 30% pooled incidence of immune-mediated AE, mostly occurring early and clinically mild and transient. Hepatotoxicity is the most commonly reported, while myocarditis and TMA remain less frequent but warrant vigilant monitoring due to potential severity. Vector serotype and high dose are associated with AEs, whereas robust peri-infusion immunosuppression might mitigate this risk. Fatalities, although rare, usually occur in the context of high vector burden and pre-existing organ compromise, underscoring the importance of careful patient selection and dosing. Future efforts should focus on elucidating the immune pathophysiology of these events and on prospectively evaluating targeted immunomodulatory strategies. Overall, our data demonstrate the safety of gene therapy for the majority of patients enrolled in published trials, but that further innovation is required to fulfill its promise. Real-world pharmacovigilance data paralleled that of trial-derived safety signals.

## Materials and methods

This meta-analysis is reported in line with the Meta-analyses Of Observational Studies in Epidemiology^10^ (Table S1) guidance and the Preferred Reporting Items for Systematic Reviews and Meta-Analyses (Table S2)^11^ statements and was registered on the international Prospective Register of Systematic Reviews (CRD420251046546).^12^

### Search strategy

The electronic databases MEDLINE, Embase, and Pubmed were comprehensively searched for English language papers from January 2005 through March 2025. The search syntax was designed for prospective and retrospective studies including patients treated with AAV gene replacement therapy and detailed in Table S3. Ongoing trial registry records were excluded. Any discrepancies in study selection were resolved through discussion and consensus between the two reviewers (N.M. and E.S.; Figure S1). A third reviewer (K.H.) was available for arbitration. If key data were missing from the published report, we planned to contact the corresponding authors for clarification. If these data remained unavailable after inquiry/no contact was possible, the study was included in analyses for which it provided sufficient data.

### Data extraction and quality assessment

Full text review and prespecified item extraction such as number of patients, disease treated, observation time, type of vector used, dose, immunosuppressive regimen, type, and clinical significance of the AE was performed independently at the study level by two independent reviewers (N.M. and E.S.). Quality assessment was performed using the Cochrane Risk of Bias tool for Clinical Trial and Observational Studies.^13^^,^^14^

### Outcomes

The primary outcome of interest was to assess the incidence of AAV gene replacement therapy immune-mediated AEs. Specifically, acute myocarditis was defined by the presence of cardiac symptoms (e.g., chest pain, dyspnea, palpitations, syncope), an elevated cardiac troponin (cTn) above the 99th percentile, and abnormal electrocardiographic and/or echocardiographic and/or Cardiac Magnetic Resonance, and/or histopathologic findings on biopsy or postmortem evaluation in the absence of flow-limiting coronary artery disease.^15^ Myocardial injury was defined as a condition defined by a cTn level above the 99th percentile upper reference limit.^15^ In patients with an increased baseline level of high-sensitivity troponin, an increase of at least 1.5 from baseline value was considered as significant.^16^ TMA was characterized by microangiopathic hemolytic anemia, thrombocytopenia, and microthrombi leading to ischemic tissue injury.^17^ AEs were assessed for clinical significance based on the need for hospitalization or end-organ functional damage. Resolution was determined if the complication was resolved at the end of the study period.

### Real-world database search

We performed an observational cross-sectional study focusing on the reporting of myocarditis, hepatitis, and TMA using two international pharmacovigilance databases, VigiBase^18^ and FAERS.^19^ Commercially available gene replacement therapy drugs were searched and two independent investigators (N.M. and E.S.) adjudicated the potential occurrence of study-defined myocarditis, hepatotoxicity, and TMA for each drug based on the information provided in the database reports (e.g., MedDRA terms, narrative summaries if available). The adjudication aimed to align pharmacovigilance reports with the clinical definitions used for the analysis. Disagreements in adjudication were resolved as detailed above.

### Statistical analysis

A random-effects meta-analysis models using restricted maximum likelihood estimation was fitted and the method of Hartung, Knapp, Sidik, and Jonkman adjustments was used to synthesize estimates and confidence intervals.^20^^,^^21^ Heterogeneity was quantified using the *I*^2^ statistic and *p* values were calculated from a χ^2^ test.^22^ Assessment of small-study effects, encompassing publication bias, outcome reporting bias, and clinical heterogeneity, was conducted statistically using the Egger test. Pooled estimates and their confidence bounds were back-transformed to the original proportion or rate scales via the inverse logit or exponential functions, and represented as percentages or events per 100 patient-years. To formally test whether AEs differed between clinical trials and observational studies, we performed a random-effects meta-regression on the logit-transformed incidence proportions. We also reconstructed an individual participant dataset (“pseudo-IPD”) by expanding each study’s published cross-tabulations (vector class × dose × immunosuppression strata event counts) into patient-level rows. We then fit one-stage population-average logistic regression models using generalized estimating equations with an exchangeable working correlation for study identifier, modeling the binary outcome of any AE. Predictor variables were AAV-vector immunogenicity (high, intermediate, or low, based on pre-existing vector immunogenicity data from the literature, i.e., the prevalence of pre-existing neutralizing antibodies for a specific serotype), viral load dose category (≤10^12^ vs. >10^12^ vg/kg) and immunosuppressive regimen. The latter were defined with *a priori* pragmatic categories as reactive corticosteroids only, pre- and post-corticosteroids, pre- and post-corticosteroids plus adjunctive immunosuppression. This grouping was chosen to preserve statistical power and to detect broad signals of effect, since these different agents act on distinct immunologic pathways. All analyses were performed in the statistical programming environment R version 4.3.1 (R Studio).

## Acknowledgments

I.O. has received grants from Bristol Meier Squibb, 10.13039/100014941Cytokinetics, 10.13039/100015362Amicus, 10.13039/100004329Genzyme, Shire, 10.13039/100004326Bayer, 10.13039/100008497Boston Scientific, Menarini International. E.A. received a grant from the 10.13039/501100003196Italian Ministry of Health (GR-2019-12368506; principal investigator of the investigator-driven MYTHS [Myocarditis Therapy with Steroids] trial) and a grant from the 10.13039/501100003196Italian Ministry of Health and 10.13039/100031478NextGenerationEU (PNRR-MAD-2022-12376225). E.S. is a research Fellow supported by 10.13039/100013915Sarnoff Cardiovascular Research Foundation. Q.B. has received a grant from the AHA (24CDA1272533). N.M. has received grants from Bristol Meier Squibb, 10.13039/100015362Amicus, Foundation CVCL, AICARM APS Onlus, Bangarter-Rhyner Foundation.

## Author contributions

N.M. was responsible for data collection, data analysis, drafting of the manuscript, critical revision. E.A. was involved in project ideation, drafting of the manuscript, and critical revision. E.S. contributed in data collection, data analysis, and drafting of the manuscript. K.H. and Q.B. were implicated in the drafting of the manuscript and critical revision. A.A. and I.O. provided critical revision of the manuscript. E.D.A. was involved in the project ideation, drafting of the manuscript, and critical revision.

## Declaration of interests

I.O. has received fees (honoraria or consulting) from Bristol Meier Squibb, Cytokinetics, Amicus, Genzyme, Shire, and Boston Scientific. E.D.A. is the Chief Scientific Officer: Lexeo Therapeutics and consultant for Kiniksa, serves in the advisory board and shareholder of Rocket Pharmaceuticals, scientific board of ResQue Therapeutics, and scientific Founder of Papillion Therapeutics. E.A. is a consultant for Kiniksa, Cytokinetics, and Lexeo Therapeutics. E.S. is a research Fellow supported by Sarnoff Cardiovascular Research Foundation. Q.B. has received fees (honoraria or consulting) from Papillon Therapeutics. N.M. has received fees (honoraria or consulting) from Bristol Meier Squibb and Academic CME and Atheneum Partners.

## Supplemental information

Document S1. Figures S1–S6 and Tables S1–S9

Document S2. Article plus supplemental information
