# Supplementary material for: Incidence, timing, and clinical significance of adverse immune events after gene replacement therapy: A systematic review and meta-analysis
Source: Mol Ther. 2026 Jan 10;34(3):1340–51. doi: 10.1016/j.ymthe.2026.01.004 (PMC12974193; doi:10.1016/j.ymthe.2026.01.004)
Supplement: Document S1. Figures S1–S6 and Tables S1–S9 [file mmc1.pdf]

YMTHE, Volume *34*

Supplemental Information

Incidence, timing, and clinical significance of adverse immune events after gene replacement therapy: A systematic review and meta-analysis

Niccolò Maurizi, Enrico Ammirati, Elizabeth Silver, Kimberly Hong, Quan Bui, Alessia Argirò, Iacopo Olivotto, and Eric D. Adler

Table S1. MOOSE (Meta-analyses Of Observational Studies in Epidemiology) Checklist.

Reporting Criteria	Reported (Yes/No)	Reported on Page No.
**Reporting of Background**		
Problem definition	Yes	1
Hypothesis statement	Yes	1
Description of Study Outcome(s)	Yes	5
Type of exposure or intervention used	Yes	4,5
Type of study design used	Yes	4,5
Study population	Yes	4,5
**Reporting of Search Strategy**		
Qualifications of searchers (eg, librarians and investigators)	Yes	4,5
Search strategy, including time period included in the synthesis and keywords	Yes	4
Effort to include all available studies, including contact with authors	Yes	4,5
Databases and registries searched	Yes	4
Search software used, name and version, including special features used (eg, explosion)	Yes	4
Use of hand searching (eg, reference lists of obtained articles)	Yes	6
List of citations located and those excluded, including justification	Yes	7
Method for addressing articles published in languages other than English	No	NA
Method of handling abstracts and unpublished studies	Yes	4
Description of any contact with authors	Yes	4
**Reporting of Methods**		
Description of relevance or appropriateness of studies assembled for assessing the hypothesis to be tested	Yes	5
Rationale for the selection and coding of data (eg, sound clinical principles or convenience)	Yes	5
Documentation of how data were classified and coded (eg, multiple raters, blinding, and interrater reliability)	Yes	5
Assessment of confounding (eg, comparability of cases and controls in studies where appropriate	Yes	5

Assessment of study quality, including blinding of quality assessors; stratification or regression on possible predictors of study results	Yes	5
Assessment of heterogeneity	Yes	5
Description of statistical methods (eg, complete description of fixed or random effects models, justification of whether the chosen models account for predictors of study results, dose-response models, or cumulative meta-analysis) in sufficient detail to be replicated	Yes	6
Provision of appropriate tables and graphics	Yes	6
**Reporting of Results**		
Table giving descriptive information for each study included	Yes	7
Results of sensitivity testing (eg, subgroup analysis)	Yes	7
Indication of statistical uncertainty of findings	Yes	7
**Reporting of Discussion**		
Quantitative assessment of bias (eg, publication bias)	Yes	7
Justification for exclusion (eg, exclusion of non–English-language citations)	No	NA
Assessment of quality of included studies	Yes	7
**Reporting of Conclusions**		
Consideration of alternative explanations for observed results	Yes	10
Generalization of the conclusions (ie, appropriate for the data presented and within the domain of the literature review)	Yes	12
Guidelines for future research	Yes	12
Disclosure of funding source	Yes	1

Table S2. Preferred Reporting Items for Systematic reviews and Meta-Analyses (PRISMA) checklist.

Section and Topic	Item #	Checklist item	Location where item is reported
**TITLE**			
Title	1	Identify the report as a systematic review.	P1
**ABSTRACT**			
Abstract	2	See the PRISMA 2020 for Abstracts checklist.	P2
**INTRODUCTION**			
Rationale	3	Describe the rationale for the review in the context of existing knowledge.	P4
Objectives	4	Provide an explicit statement of the objective(s) or question(s) the review addresses.	P4
**METHODS**			
Eligibility criteria	5	Specify the inclusion and exclusion criteria for the review and how studies were grouped for the syntheses.	P5-P6
Information sources	6	Specify all databases, registers, websites, organisations, reference lists and other sources searched or consulted to identify studies. Specify the date when each source was last searched or consulted.	P4-P5-P6
Search strategy	7	Present the full search strategies for all databases, registers and websites, including any filters and limits used.	P4-P5-P6
Selection process	8	Specify the methods used to decide whether a study met the inclusion criteria of the review, including how many reviewers screened each record and each report retrieved, whether they worked independently, and if applicable, details of automation tools used in the process.	P4-P5
Data collection process	9	Specify the methods used to collect data from reports, including how many reviewers collected data from each report, whether they worked independently, any processes for obtaining or confirming data from study investigators, and if applicable, details of automation tools used in the process.	P5
Data items	10a	List and define all outcomes for which data were sought. Specify whether all results that were compatible with each outcome domain in each study were sought (e.g. for all measures, time points, analyses), and if not, the methods used to decide which results to collect.	P5
	10b	List and define all other variables for which data were sought (e.g. participant and intervention characteristics, funding sources). Describe any assumptions made about any missing or unclear information.	P5
Study risk of bias assessment	11	Specify the methods used to assess risk of bias in the included studies, including details of the tool(s) used, how many reviewers assessed each study and whether they worked independently, and if applicable, details of automation tools used in the process.	P5
Effect measures	12	Specify for each outcome the effect measure(s) (e.g. risk ratio, mean difference) used in the synthesis or presentation of results.	P7
Synthesis methods	13a	Describe the processes used to decide which studies were eligible for each synthesis (e.g. tabulating the study intervention characteristics and comparing against the planned groups for each synthesis (item #5)).	P6-P7
	13b	Describe any methods required to prepare the data for presentation or synthesis, such as handling of missing summary statistics, or data conversions.	P5
	13c	Describe any methods used to tabulate or visually display results of individual studies and syntheses.	P5
	13d	Describe any methods used to synthesize results and provide a rationale for the choice(s). If meta-analysis was performed, describe the model(s), method(s) to identify the presence and extent of statistical heterogeneity, and software package(s) used.	P6
	13e	Describe any methods used to explore possible causes of heterogeneity among study results (e.g. subgroup analysis, meta-regression).	P6
	13f	Describe any sensitivity analyses conducted to assess robustness of the synthesized results.	P6
Reporting bias assessment	14	Describe any methods used to assess risk of bias due to missing results in a synthesis (arising from reporting biases).	P6
Certainty assessment	15	Describe any methods used to assess certainty (or confidence) in the body of evidence for an outcome.	P6

**RESULTS**			
Study selection	16a	Describe the results of the search and selection process, from the number of records identified in the search to the number of studies included in the review, ideally using a flow diagram.	P7
	16b	Cite studies that might appear to meet the inclusion criteria, but which were excluded, and explain why they were excluded.	P7
Study characteristics	17	Cite each included study and present its characteristics.	P7
Risk of bias in studies	18	Present assessments of risk of bias for each included study.	P7
Results of individual studies	19	For all outcomes, present, for each study: (a) summary statistics for each group (where appropriate) and (b) an effect estimate and its precision (e.g. confidence/credible interval), ideally using structured tables or plots.	P7
Results of syntheses	20a	For each synthesis, briefly summarise the characteristics and risk of bias among contributing studies.	P7
	20b	Present results of all statistical syntheses conducted. If meta-analysis was done, present for each the summary estimate and its precision (e.g. confidence/credible interval) and measures of statistical heterogeneity. If comparing groups, describe the direction of the effect.	P7-P8
	20c	Present results of all investigations of possible causes of heterogeneity among study results.	P7-P8
	20d	Present results of all sensitivity analyses conducted to assess the robustness of the synthesized results.	P7
Reporting biases	21	Present assessments of risk of bias due to missing results (arising from reporting biases) for each synthesis assessed.	P7
Certainty of evidence	22	Present assessments of certainty (or confidence) in the body of evidence for each outcome assessed.	P7
**DISCUSSION**			
Discussion	23a	Provide a general interpretation of the results in the context of other evidence.	P9-P10
	23b	Discuss any limitations of the evidence included in the review.	P12-P13
	23c	Discuss any limitations of the review processes used.	P12-P13
	23d	Discuss implications of the results for practice, policy, and future research.	P13
**OTHER INFORMATION**			
Registration and protocol	24a	Provide registration information for the review, including register name and registration number, or state that the review was not registered.	P4
	24b	Indicate where the review protocol can be accessed, or state that a protocol was not prepared.	P4
	24c	Describe and explain any amendments to information provided at registration or in the protocol.	NA
Support	25	Describe sources of financial or non-financial support for the review, and the role of the funders or sponsors in the review.	P1
Competing interests	26	Declare any competing interests of review authors.	P1
Availability of data, code and other materials	27	Report which of the following are publicly available and where they can be found: template data collection forms; data extracted from included studies; data used for all analyses; analytic code; any other materials used in the review.	P4

Table S3. Search criteria for each database and the query used for the search.

Database	Query	Results
Embase<br><br>Embase<br><br>Pubmed	(‘gene replacement therapy’/exp OR ‘gene replacement') AND (’gene therapy trial'/exp OR ‘gene therapy study' OR ‘AAV replacement therapy' OR ‘AAV replacement trial' OR ‘gene therapy trial'/exp OR ‘gene therapy adverse effect' OR ‘gene replacement adverse effect'/exp OR ‘AAV replacement adverse effects' OR ‘real world gene therapy adverse effects' OR ‘real world gene replacement therapy adverse effects'	802

Table S4 Overview of studies included in the systematic-review and meta-analysis.

Abbreviations: AAV: Adenovirus; DMD: Duchenne Muscolar Dystrophy; MD: Muscolar Dystrophy; SMA: Spinal Muscle Atrophy. Follow-up is presented in months and age in years or in months, when specifically indicated.

Study	Year	Diease	Follow-up	Age	Patients	AAV Delivery Vector	Total immune mediated adverse events	Myocarditis	Hepatotoxicity	Thrombotic microangiopathy	Pre-Delivery Immunosuppression	Post-therapy immunosuppression
Greenber et al.	2024[23]	Danon	24	8-20	7	AAV-9	1	0	0	1	Rituximab + Sirolimus	Prednisone, Tacrolimus/Sirolimus, Rituximab
Hughes et al.	2022[24]	Fabry	13	22-48	6	AAV-2	0	0	0	0	-	-
Mendell et al.	2024[25]	DMD	12	-	4	AAVrh74	0	0	0	3	Corticosteroids	Corticosteroids
Bonenemann et al.	2023[26]	DMD	0,3	2	1	AAV-8	1	1	1	0	Corticosteroids	Corticosteroids
Mendell et al.	2020[27]	DMD	12	4-6	4	AAVrh74	1	0	1	0	Prednisone	Prednisone
Zaidman et al.	2023[28]	DMD	12	4-8	20	AAVrh74	2	1	1	0	Corticosteroids	Corticosteroids
Mendell et al.	2023[29]	DMD	12	4-12	20	AAVrh74	0	0	0	0	Corticosteroids	Corticosteroids
Dreghici et al.	2022[30]	DMD	36	-	9	AAV-9	4	0	4	0	-	-
Laugel et al.	2024[31]	DMD	4	6-10	3	AAV2/8	1	0	1	0	Sirolimus	Sirolimus. Corticosteroids
Lek et al.	2023[32]	DMD	0.33	27	1	AAV-9	1	0	1	0	Rituximab, Corticosteroids	Sirolimus, Corticosteroids, Eculizumab, Rituximab, Toculizumab, IVIG
Mendell et al.	2025[33]	DMD	4	4-8	63	AAVrh74	6	1	5	0	Corticosteroids	Corticosteroids
Flaning et al.	2022[34]	DMD	12		2	rAAVrh74	0	0	0	0	Corticosteroids	Corticosteroids
Bowles et al.	2012[35]	DMD	4	15-24	6	AAV 2/5	0	0	0	0	Corticosteroids	Corticosteroids
Mendell et al.	2010[36]	Limb-Girdle MD 2B	6	11-43	6	rAVV-1	0	0	0	0	Corticosteroids	Corticosteroids
Mendell et al.	2024[37]	Limb-Girdle MD 2B	13	4-7	6	rAAV-rh74	1	0	1	0	Corticosteroids	Corticosteroids
Smith et al.	2023[38]	Pompe	13	52-71	3	AAV-8	0	0	0	0	No	Corticosteroids
Smith et al.	2013[39]	Pompe	6	-	5	rAAV (?)	0	0	0	0	No	Corticosteroids
Corti et al.	2018[40]	Pompe	6	2-18	9	AAV-1	0	0	0	0	Rituximab, Sirolimus	Corticosteroids
Strauss et al.	2022[41]	SMA	14	6 m	15	scAAV9	5	2	3	0	Corticosteroids	Corticosteroids
Strauss et al.	2022[42]	SMA	14	6 m	14	scAAV9	4	1	3	0	Corticosteroids	Corticosteroids
Mendell et al.	2017[43]	SMA	24	6 m	15	scAAV9	2	0	2	0	Corticosteroids	Corticosteroids
Day et al.	2021[44]	SMA	12	6 m	20	scAAV9	2	0	2	0	Corticosteroids	Corticosteroids
Mercuri et al.	2021[45]	SMA	12	6 m	33	scAAV9	9	0	9	0	Corticosteroids	Corticosteroids
Goilou et al.	2022[46]	SMA	1	4 m	1	scAAV9	1	0	0	1	Corticosteroids	Corticosteroids
Chand et al.	2021[47]	SMA	16	4-6 m	3	scAAV9	3	0	0	3	Corticosteroids	Corticosteroids
Finekel et al.	2023[48]	SMA	6	4-10 m	32	scAAV9	2	0	0	2	Corticosteroids	Corticosteroids
Gowda et al.	2022[49]	SMA	6	4-14 m	99	scAAV9	99	29	70	0	Corticosteroids	Corticosteroids
Servais et al.	2024[50]	SMA	5	4-14 m	168	scAAV9	67	18	49	1	Corticosteroids	Corticosteroids
Weib et al.	2022[51]	SMA	6	4-14 m	76	scAAV9	58	2	56	0	Corticosteroids	Corticosteroids
Bitetti et al.	2023[52]	SMA	3	4-14 m	9	scAAV9	6	1	5	0	Corticosteroids	Corticosteroids
Mendell et al.	2021[53]	SMA	60	4-14 m	10	scAAV9	0	0	0	0	Corticosteroids	Corticosteroids
Favia et al.	2024[54]	SMA		4-14 m	8	scAAV9	0	0	0	0	Corticosteroids	Corticosteroids
Waldrop et al.	2024[55]	SMA	/	4-14 m	46	scAAV9	18	0	18	0	Corticosteroids	Corticosteroids
Waldrop et al.	2020[56]	SMA	12	4-14 m	21	scAAV9	3	0	3	0	Corticosteroids	Corticosteroids
Chencheri et al.	2023[57]	SMA	6	4-14 m	25	scAAV9	1	0	1	0	Corticosteroids	Corticosteroids
Pane et al.	2023[58]	SMA	/	4-14 m	46	scAAV9	0	0	0	0	Corticosteroids	Corticosteroids
Tokaltly et al.	2023[59]	SMA	12	4-14 m	25	scAAV9	25	10	18	0	Corticosteroids	Corticosteroids
Stettner et al.	2023[60]	SMA	/	4-14 m	9	scAAV9	6	2	4	0	Corticosteroids	Corticosteroids
Matesanz et al.	2021[61]	SMA	/	4-14 m	7	scAAV9	6	0	6	0	Corticosteroids	Corticosteroids
Gaber et al.	2021[62]	SMA	3	4-14 m	9	scAAV9	7	0	7	0	Corticosteroids	Corticosteroids
D'Silva et al.	2022[63]	SMA	12	4-14 m	21	scAAV9	8	0	6	2	Corticosteroids	Corticosteroids
Friese et al.	2021[64]	SMA	6	4-14 m	9	scAAV9	2	0	2	0	Corticosteroids	Corticosteroids
Shieh et al.	2023[65]	X-Lynked Myotub Myo	12	2.5-5	26	AAV-8	9	4	7	0	Corticosteroids	Corticosteroids
Ozelo et al.	2022[66]	Hem A	13	19-43	134	AAV5	108	0	108	0	No	Reactive Corticosteroids
Ozelo et al.	2025[67]	Hem A	12	19-41	22	AAV-5	22	0	22	0	Corticosteroids	Corticosteroids
Manno et al.	200[73]	Hem B	2	-	7	rAVV-2	2	0	2	0	-	-
Pipe et al.	2023[74]	Hem B	13	19-75	54	AAV-5	11	0	11	0	No	Reactive Corticosteroids
Rangarajan et al.	2017[68]	Hem A	13	18-75	9	AAV-5	8	0	8	0	No	Corticosteroids
Mahlangu et al.	2024[69]	Hem A	52	18-47	132	AAV-5	108	0	108	0	No	Reactive Corticosteroids
Leavitt et al.	2024[70]	Hem A	26	19-47	11	rAAV-6	5	0	5	0	No	Corticosteroids
Chapint et al.	2017[71]	Hem A	36	18-45	4	AAV-8	4	0	4	0	No	Reactive Corticosteroids
George et al.	2017[72]	Hem B	12	18-53	10	rAAV-FIX	1	0	1	0	No	Reactive Corticosteroids
George et al.	2021[75]	Hem A	3	18-52	18	AAV-3	7	0	7	0	No	Reactive Corticosteroids
Nathwaani et al.	2011[76]	Hem B	3	31-64	6	scAAV2/8	2	0	2	0	No	Reactive Corticosteroids
Chowdary et al.	2022[77]	Hem B	12	29-67	10	AAV-3	8	0	8	0	No	Corticosteroids, Reactive Tacrolimus

Chowdary et al.	2022[77]	Hem B	12	29-67	10	AAV-3	8	0	8	0	No	Corticosteroids, Reactive Tacrolimus
Coppens et al.	2024[79]	Hem B	24	19-75	54	AAV-2	17	0	17	0	No	Reactive Corticosteroids
Cuker et al.	2024[78]	Hem B	15	18-62	45	AAV-5-FIX	28	0	28	0	No	Corticosteroids
Tai et al.	2022[80]	AADC Def	12	3-16	26	AAV-2	0	0	0	0	No	No
Lv et al.	2024[81]	Aut Rec Deaf	2	1-18	6	AAV-1	0	0	0	0	No	No
Sevignz et al.	2024[82]	Front Deme	12	78-86	19	AAV-9	0	0	0	0	Corticosteroids, Sirolimus, Rituximab	Corticosteroids, Sirolimus, Rituximab
D'Antigo et al.	2023[83]	Crigler Najar	4	18-27	5	AAV-8	4	0	4	0	Corticosteroids, Sirolimus	Corticosteroids, Sirolimus
Tardieu et al.	2014[85]	Mucopo lys	12	1-5	4	AAVrh10	0	0	0	0	Corticosteroids	Corticosteroids, Tacrolimus, Mycophenolate
Tardieu et al.	2017[86]	Mucopo lys	12	1-5	4	rAAV-2/8	0	0	0	0	Corticosteroids	Corticosteroids, Tacrolimus, Mycophenolate
Deiva et al.	2021[87]	Mucopo lys	50	1-5	4	rAAV-2/8	0	0	0	0	Corticosteroids	Corticosteroids, Tacrolimus, Mycophenolate
Brunetti-Pierri et al.	2022[84]	Mucopo lys	13	10-29	9	AAV-2/8	2	0	2	0	Corticosteroids	Corticosteroids
Mendell et al.	2015[88]	Becker MD	12	24-37	6	AAV-1	0	0	0	0	Corticosteroids	Corticosteroids
Lyon et al.	2020[89]	CHF	2	29-69	5	AAV-1	0	0	0	0	No	No
Hammond et al.	2016[90]	CHF	12	18-80	42	AAV-5	0	0	0	0	No	No
Stewart et al.	2006[96]	Angina	6	65-78	33	Ad5	0	0	0	0	No	No
Jasky et al.	2009[91]	CHF	12	-	9	AAV-1	0	0	0	0	No	No
Jessup et al.	2011[92]	CHF	12	-	39	AAV-1	0	0	0	0	No	No
Greenber et al.	2016[93]	CHF	12	18-80	123	AAV-1	0	0	0	0	No	No
Grines et al.	2002[94]	Stable Angina	10	35-67	60	AAV-5	2	0	2	0	No	No
Grines et al.	2003[95]	Stable Angina	19	-	52	AAV-5	10	0	10	0	No	No
Nakamura et al.	2024[96]	Stable Angina	12	39-80	32	AAV-5	0	0	0	0	No	No
D'Avola et al.	2016[97]	Porphyri a	12	1-13	8	AAV-2/5	0	0	0	0	No	No
Ferreira et al.	2014[98]	Lipase Def	3	-	5	AAV-1	0	0	0	0	Corticosteroids	Corticosteroids
Weinstein et al.	2025[99]	Gly Stor IA	13	19-57	12	AAV-2/8	12	0	12	0	No	Reactive and prophylactic Corticosteroids
Flotte et al.	2022[100]	Tay-Sachs	6	12-14 m	2	AAVrh8	0	0	0	0	Corticosteroids	Corticosteroids
Priddy et al.	2019[101]	HIV	12	18-45	21	AAV-1	0	0	0	0	-	-
Flotte et al.	2011[102]	AAT Deficency	6	-	9	rAAV	0	0	0	0	Corticosteroids	Corticosteroids

Table S5 Critical appraisal of eligible clinical trial and risk of bias using the Cochrane risk-of-bias tool for randomized trials (RoB 2.0).

Domains: D1: Bias arising from the randomization process. D2: Bias due to deviations from intended intervention. D3: Bias due to missing outcome data. D4: Bias in measurement of the outcome.

D5: Bias in selection of the reported result. Judgment: + = Low; - = Some concerns; ? = No information; X = high.

Low Risk

Some concerns

High Risk

No information

Citation	D 1 (randomisation)	D 2 (intended intervention)	D 3 (missing outcome)	D 4 (mesaurment of outcome)	D 5 (selection of results)	Risk of Bias
Greenberg et al. 2025	+	+	+	+	+	+
Hughes et al. 2022	+	+	-	-	-	-
Mendell et al. 2024	+	+	-	-	-	-
Mendell et al. 2020	+	+	+	+	+	+
Zaidman et al. 2023	+	+	+	+	+	+
Mendell et al. 2023	+	+	+	+	+	+
Dreghich et al. 2022	+	+	-	-	-	-
Laugel et al. 2024	+	+	+	+	+	+
Mendell et al. 2025	+	+	+	+	+	+
Flaningn et al. 2022	+	+	+	+	+	+
Bowles et al. 2012	+	+	+	+	+	+
Mendell et al. 2024	+	+	+	+	+	+
Mendell et al. 2010	+	+	+	+	+	+
Smith et al. 2023	+	+	+	+	+	+
Smith et al. 2013	+	+	+	+	+	+
Corti E. et al. 2018	+	+	+	+	+	+
Strauss et al. 2022	+	+	+	+	+	+
Strauss et al. 2022	+	+	+	+	+	+
Mendell et al. 2017	+	+	+	+	+	+
Day et al. 2021	+	+	+	+	+	+
Shieh et al. 2023	+	+	+	+	+	+
Ozelo et al. 2022	+	+	+	+	+	+
Ozelo et al. 2025	+	+	+	+	+	+
Manno et al. 2006	+	X	+	+	+	-
Pipe et al. 2023	+	+	+	+	+	+
Rangarajan et al. 2017	+	+	+	+	+	+
Mahlangu et al. 2024	+	+	+	+	+	+
Leavitt et al. 2024	+	+	+	+	+	+
Chapint. et al. 2017	+	+	+	+	+	+
George L. et al. 2017	+	+	+	+	+	+
George et al. 2021	+	+	+	+	+	+
Mendell et al. 2021	+	+	+	+	+	+

Citation	D 1 (randomisation)	D 2 (intended intervention)	D 3 (missing outcome)	D 4 (mesaurment of outcome)	D 5 (selection of results)	Risk of Bias
Nathwani et al. 2011	-	+	+	+	+	+
Chowdary et al. 2022	+	+	+	+	+	+
Cuker et al. 2024	+	+	+	+	+	+
Coppens et al. 2024	+	+	+	+	+	+
Tai et al. 2022	+	+	+	+	+	+
Lv et al. 2024	+	+	+	+	+	+
Sevignz et al. 2024	+	+	-	-	-	-
D'antigo et al. 2023	+	+	+	+	+	+
Brunetti-Pierri et al. 2022	-	+	+	+	+	-
Tardieu et al. 2014	+	+	+	+	-	-
Tardieu et al. 2017	+	+	+	+	-	-
Deiva et al. 2021	+	+	+	+	+	+
Mendell et al. 2015	+	+	+	+	+	+
Lyon et al. 2020	+	+	+	+	+	+
Hammond et al. 2016	+	+	+	+	+	+
Grines et al. 2002	+	+	+	+	+	+
Grines et al. 2003	+	+	+	+	+	+
Jasky et al. 2009	+	+	+	+	+	+
Jessup et al. 2011	+	+	+	+	+	+
Greenberg et al. 2016	+	+	+	+	+	+
D'Avola et al. 2016	+	+	+	+	+	+
Ferreira et al. 2014	+	?	+	+	+	-
Weinsten et al. 2025	+	+	+	+	+	+
Flotte et al. 2022	+	+	+	+	+	+
Priddy et al. 2019	+	+	+	+	+	+
Flotte et al. 2011	+	+	+	+	+	+
Nagamura et al. 2024	+	+	+	+	+	+

Table S6. Critical appraisal of eligible observational studies and risk of bias using the Cochrane risk-of-bias tool for randomized trials (RoB 1.0).

Domains: D1: Bias arising from cofounding. D2: Bias due to selection. D3: Bias due classification of interventions. D4: Bias due to deviation from intendended intervention.
D5: Bias due to missing data. D6: Bias due to mesaurements of outcome. D7: Bias due to selection of reported outcomes. Judgment: + = Low; - = Some concerns; ? = No information; X = high.

Citation	D 1 (Cofounding)	D 2 (Selection)	D 3 (Classificiaetion of interventions)	D 4 (Deviation from intendend interventions)	D 5 (missing data)	D 6 (measurements of outcome)	D 7 (Selection of reported outcome)	Risk of Bias
Bonnemann et al. 2023	+	-	+	+	+	+	+	-
Lek et al. 2023	-	-	+	+	+	+	+	-
Guilou et al. 2022	+	-	+	+	+	+	+	-
Chand et al. 2021	+	+	+	+	+	+	+	+
Finkel et al. 2023	+	+	+	+	+	+	+	+
Gowda et al. 2024	+	+	+	+	+	+	+	+
Servais et al. 2024	+	+	+	+	+	+	+	+
Weib et al. 2022	+	+	+	+	+	+	+	+
Bitetti et al. 2023	-	-	+	+	+	+	+	-
Favia et al. 2024	-	+	+	+	+	+	+	-
Waldrop et al. 2024	+	+	+	+	+	+	+	+
Waldrop et al. 2020	+	+	+	+	+	+	+	+
Chencheri et al. 2023	+	+	+	+	+	+	+	+
Waldrop et al. 2024	+	+	+	+	+	+	+	+
Waldrop et al. 2020	+	+	+	+	+	+	+	+
Chencheri et al. 2023	+	+	+	+	+	+	+	+
Pane et al. 2023	+	+	+	+	+	+	-	-
Tokatly Latzer et al.	+	+	+	+	+	+	+	+
Stettner et al.	+	+	+	+	+	+	+	+
Matesanz et al.	-	+	+	+	+	+	+	-
Gaber ali et al.	+	+	+	+	+	+	+	+
D'Silva et al	+	+	+	+	+	+	+	+
Friese et al.	+	+	+	+	+	+	+	+

Low Risk | Some concerns | High Risk | No information

Table S7. List of missing data per each study incliuded in the analysis.

Study	Year	Missing Adverse Event Description	Missing Vector Type	Missing Vector Dose	Missing Immunosuppressive Regimen	Missing Follow-up data
Greenber et al.	2024	0	0	0	0	0
Hughes et al.	2022	0	0	0	0	0
Nakamura et al.	2023	0	0	0	0	0
Mendell et al.	2023	0	0	0	0	0
Zaidman et al.	2023	0	0	0	0	0
Bonnemann et al.	2023	0	0	0	0	0
Dreghici et al.	2022	0	0	0	0	0
Laugel et al.	2024	0	0	0	0	0
Lek et al.	2023	0	0	0	0	0
Mendell et al.	2025	0	0	0	0	0
Flaning et al.	2022	0	0	0	0	0
Bowles et al.	2012	0	0	0	0	0
Mendell et al.	2010	0	0	0	0	0
Mendell et al.	2024	0	0	0	0	0
Smith et al.	2023	0	0	0	0	0
Smith et al.	2013	0	0	0	0	0
Corti et al.	2018	0	0	0	0	0
Strauss et al.	2022	0	0	0	0	0
Strauss et al.	2022	0	0	0	0	0
Mendell et al.	2017	0	0	0	0	0
Day et al.	2021	0	0	0	0	0
Merci et al.	2021	0	0	0	0	0
Goilou et al.	2022	0	0	0	0	1
Chand et al.	2021	0	0	0	0	0
Finekel et al.	2023	0	0	0	0	0
Gowda et al.	2022	0	0	0	0	0
Servais et al.	2024	0	0	0	0	0
Weib et al.	2022	0	0	0	0	1
Bitetti et al.	2023	0	0	0	0	0
Mendell et al.	2021	0	0	0	0	0
Favia et al.	2024	0	0	0	0	1
Waldrop et al.	2024	0	0	0	0	0
Waldrop et al.	2020	0	0	0	0	0
Chencheri et al.	2023	0	0	0	0	0
Pane et al.	2023	0	0	0	0	0
Tokaltly et al.	2023	0	0	0	0	1
Stettner et al.	2023	0	0	0	0	0
Matesanz et al.	2021	0	0	0	0	0
Gaber et al.	2021	0	0	0	0	0
D’Silva et al.	2022	0	0	0	0	0
Friese et al.	2021	0	0	0	0	0
Shieh et al.	2023	0	0	0	0	0
Ozelo et al.	2022	0	0	0	0	0
Ozelo et al.	2025	0	0	0	0	0
Manno et al.	2006	0	0	0	0	0
Pipe et al.	2023	0	0	0	0	0
Rangarajan et al.	2017	0	0	0	0	0
Mahlangu et al.	2024	0	0	0	0	0
Leavitt et al.	2024	0	0	0	0	0
Chapint et al.	2017	0	0	0	0	0
George et al.	2017	0	0	0	0	0
George et al.	2021	0	0	0	0	0
Nathwaani et al.	2011	0	0	0	0	0
Chowdary et al.	2022	0	0	0	0	0
Coppens et al.	2024	0	0	0	0	0
Cuker et al.	2024	0	0	0	0	0
Tai et al.	2022	0	0	0	0	0
Lv et al.	2024	0	0	0	0	0
Sevignz et al.	2024	0	0	0	0	0
D’Antigo et al.	2023	0	0	0	0	0
Tardieu et al.	2014	0	0	0	0	0
Tardieu et al.	2017	0	0	0	0	0
Deiva et al.	2021	0	0	0	0	0
Brunetti-Pierri et al.	2022	0	0	0	0	0
Mendell et al.	2015	0	0	0	0	0
Lyon et al.	2020	0	0	0	0	0
Hammond et al.	2016	0	0	0	0	0
Jasky et al.	2009	0	0	0	0	0
Jessup et al.	2011	0	0	0	0	0
Greenber et al.	2016	0	0	0	0	0
Grines et al.	2002	0	0	0	0	0
Grines et al.	2003	0	0	0	0	0
D’Avola et al.	2016	0	0	0	0	0
Ferreira et al.	2014	0	0	0	0	0
Weinstein et al.	2025	0	0	0	0	0
Flotte et al.	2022	0	0	0	0	0
Priddy et al.	2019	0	0	0	1	0
Flotte et al.	2011	0	0	0	0	0

Table S8. Characteristics of the 72 myocarditis episodes related to immune activation after gene replacement therapy.

Abbreviations: AAV: Adenovirus; DMD: Duchenne Muscolar Dystrophy; EF: Ejection Fraction; PVCs: Premature Ventricular Contractions; PLT: Platelets; ARDS: Acute Respiratory Distress Syndrome; ECMO: Extracorporeal Membrane Oxygenation; MOF: Multi Organ Failure; LDH: Lactate Dehydrogenase; AST: Aspartate Transaminase; ALT: Alaniine Transaminase; UNL: Upper Normal Limit; Peric Effusion: Pericardial Effusion.

Patients	Study	Age	Disease	AAV Delivery Vector	Dose	Pre-Delivery Immunosuppression	Post-therapy immunosuppression	Pre-existing Conditions	Clinical Course	Echocardiogram	Explanation	Outcome
Case 1	Zaidman et al. 2023[28]	7	DMD	AAVrh74 + MHCK7 promoter	IV 1.3 x 10^{14} vg/kg	Corticosteroids	Corticosteroids (p.o. and I.V.)	Large deletion from exon 8 to 21 in dystrophion gene	3 weeks: severe diffuse muscular weakness. Myositis. ↑ TnnI, focal wall motion abnormalities decreased EF, ↑ T2 at Cardiac MR (CMR)	focal wall motion abnormalities decreased EF	Microdystrophin construct with hing 1 and the beginning of the spectrin like domain, absent in the patients and acting as 'non-self' epitope.	Resolved during follow-up
Case 2	Bonneman et al. 2023[26]	9	DMD	AAV-8	IV 1.3 x 10^{14} vg/kg	Corticosteroids	Corticosteroids (p.o. and I.V.)	Large deletion from exon 8 to 21 in dystrophion gene	3 weeks: severe diffuse muscular weakness. Myositis. ↑ TnnI, focal wall motion abnormalities decreased EF, ↑ T2 at Cardiac MR (CMR)	focal wall motion abnormalities decreased EF	Microdystrophin construct with hing 1 and the beginning of the spectrin like domain, absent in the patients and acting as 'non-self' epitope.	Resolved during follow-up
Case 3	Mendell et al. 2025[35]	-	DMD	rAAVrh74	IV 1.3 x 10^{14} vg/kg	Corticosteroids	Corticosteroids (p.o.)	-	Day 0: ↑ Tnn, vomiting, fever. Transient spontaneuous hypotension. No significant change in the echocardiogram.	No changes	Probable immune response of to the vecto caspid	Resolved during follow-up
Case 4	Lek et al. 2023[32]	27	DMD	rAAV-9 dSaCas9	IV 1 x 10^{14} vg/kg	Corticosteroids	Day 0: Corticosteroids<br>Day 1: Eculizumab<br>Day 6: Eculizumab, Tocilizumab, Anakinra	Lean muscle mass 45%<br>Restrictive Pulmonary Defect<br>Mild DMD Cardiomyopathy (EF 55%)	Day 1: PVCs, ↓ PLT<br>Day 5: ↓ EF (45-50%), ↑ TnnI, peric effusion. Day 6: ARDS ; Day 8: ECMO and death from MOF and hypoxic neurologic injury	Decreased EF (45-50%)	Cytokine-mediated Capillary Leak Syndrome due to treatment acute toxic effect	Death<br>(at autopsy severe DMD cardiomyopathy)
Case 5 - 7	Strauss et al. 2022[42]	6 m	SMA	scAAV-9-FL-SMNcDNA	IV 1.1 x 10^{14} vg/kg	Corticosteroids	Corticosteroids	-	Week 1: ↑ Tnn and Ck-MB	-	Probable immune response of to the vecto caspid	Resolved during follow-up
Case 8 to 36	Gowda et al. 2023[49]	6-10 m	SMA	scAAV-9-FL-SMNcDNA	IV 1.1 x 10^{14} vg/kg	Corticosteroids	Corticosteroids	-	Week 2: ↑ Tnn	Normal	Probable immune response of to the vecto caspid	Resolved during follow-up
Case 37 to 54	Servais et al. 2024[50]	4-14 m	SMA	scAAV-9-FL-SMNcDNA	IV 1.1 x 10^{14} vg/kg	Corticosteroids	Corticosteroids	-	Week 2: ↑ Tnn	Normal	Probable immune response of to the vecto caspid	Resolved during follow-up
Case 55 - 56	Weib et al. 2022[51]	12-16 m	SMA	scAAV-9-FL-SMNcDNA	1.1 x 10^{14} vg/kg	Corticosteroids	Corticosteroids	-	Week 1: ↑ Tnn	Normal	Probable immune response of to the vecto caspid	Resolved during follow-up
Case 57	Bitetti et al. 2023[52]	12-16 m	SMA	scAAV-9-FL-SMNcDNA	IV 1.1 x 10^{14} vg/kg	Corticosteroids	Corticosteroids	-	Week 2: ↑ Tnn	Normal	Probable immune response of to the vecto caspid	Resolved during follow-up
Case 58 to 67	Tokaty et al. 2023[59]	6 m -18 m	SMA	scAAV-9-FL-SMNcDNA	IV 1.1 x 10^{14} vg/kg	Corticosteroids	Corticosteroids	-	Week 2: ↑ Tnn	Normal	Probable immune response of to the vecto caspid	Resolved during follow-up
Case 68 - 69	Stettner et al. 2023[60]	6 m	SMA	scAAV-9-FL-SMNcDNA	IV 1.1 x 10^{14} vg/kg	Corticosteroids	Corticosteroids	-	Week 2: ↑ Tnn	Normal	Probable immune response of to the vecto caspid	Resolved during follow-up
Case 70 - 71	Shieh et al. 2023[65]		X-Lynk Myotub Myop	AAV-8	IV 3.5 x 10^{14} vg/kg	Corticosteroids	Corticosteroids + Sirolimus + Mycophenolate Mofetil	-	Within week 3: ↑ Tnn	Normal	Probable immune response of to the vecto caspid	Resolved during follow-up

Table S9. Characteristics of the 6 death cases related to immune activation after gene replacement therapy.

Abbreviations: AAV: Adenovirus; DMD: Duchenne Muscolar Dystrophy; EF: Ejection Fraction; PVCs: Premature Ventricular Contractions; PLT: Platelets; ARDS: Acute Respiratory Distress Syndrome; ECMO: Extracorporeal Membrane Oxygenation; MOF: Multi Organ Failure; LDH: Lactate Dehydrogenase; AST: Aspartate Transaminase; ALT: Alaniine Transaminase; UNL: Upper Normal Limit

Patients	Study	Age	Disease	AAV Delivery Vector	Dose	Pre-Delivery Immunosuppression	Post-therapy immunosuppression	Pre-existing Conditions	Clinical Course	Cause of death
Case 1	Lek et al. 2023[32]	27	DMD	rAAV-9 dSaCas9	IV 1 x 10^{14} vg/kg	Corticosteroids	Day 0: Corticosteroids<br>Day 1: Eculizumab<br>Day 6: Eculizumab, Tocilizumab, Anakinra	Lean muscle mass 45%<br>Restrictive Pulmonary Defect<br>Compensated Cardiomyopathy (EF 55%)	Day 1: PVCs, ↓ PLT<br>Day 5: Decreased EF (45-50%), ↑ TnnI, pericardial effusion.<br>Day 6: ARDS<br>Day 8: ECMO and death from MOF and hypoxic neurologic injury	Cytokine-mediated Capillary Leak Syndrome with cardiac dysfunction due to treatment acute toxic effect<br>(at autopsy presence of severe DMD cardiomyopathy without evidence of active inflammation)
Case 2	Guillou et al. 2022[46]	4 m	SMA	scAAV9	IV 1.1 x 10^{14} vg/kg	Corticosteroids	Corticosteroids<br>Day 12: Eculizumab	VUS in Complement Factor 1 Gene	Day 8: Vomiting, ↓ PLT, ↑ LDH, ↑ AST/ALT<br>Day 12: Acute Renal Failure and Haemolytic Anemia<br>Day 30: Cardiac Arrest	MOF with severe dysautonomia, hypovolemia, sepsis in a context of TMA
Case 3	Shieh et al. 2023[65]	5.6	X-Lynked Myotubular Myopathy	AAV-8	IV 1.3 x 10^{14} vg/kg	Corticosteroids	Corticosteroids, Anakinra, Tocilizumab, Ruxolitinib	pre-existing hepatobiliary vulnerability<br>Absence of liver peliosis	Within 1-4 weeks:<br>Hepatopathy, Severe Immune dysfunction, Ascites, Cholestatic Liver Failure with AST and ALT > 5 UNL	Sepis in a context of liver failure
Case 4	Shieh et al. 2023[65]	4.8	X-Lynked Myotubular Myopathy	AAV-8	IV 3.5 x 10^{14} vg/kg	Corticosteroids	Corticosteroids, Anakinra, Tocilizumab, Ruxolitinib	pre-existing hepatobiliary vulnerability<br>Absence of liver peliosis	Within 1-4 weeks:<br>Hepatopathy, Severe Immune dysfunction, Ascites, Cholestatic Liver Failure with AST and ALT > 5 UNL	Psudomonas Sepsis in a context of liver failure
Case 5	Shieh et al. 2023[65]	6.1	X-Lynked Myotubular Myopathy	AAV-8	IV 3.5 x 10^{14} vg/kg	Corticosteroids	Corticosteroids, Anakinra, Tocilizumab, Ruxolitinib	pre-existing hepatobiliary vulnerability<br>Absence of liver peliosis	Within 1-4 weeks:<br>Hepatopathy, Severe Immune dysfunction, Ascites, Cholestatic Liver Failure with AST and ALT > 5 UNL	Circulatory Collapse due to Gastrointestinal Bleeding in a context of liver failure
Case 6	Shieh et al. 2023[65]	2.5	X-Lynked Myotubular Myopathy	AAV-8	IV 3.5 x 10^{14} vg/kg	Corticosteroids	Corticosteroids, Anakinra, Tocilizumab, Ruxolitinib	pre-existing hepatobiliary vulnerability<br>Absence of liver peliosis	Within 1-4 weeks:<br>Hepatopathy, Severe Immune dysfunction, Ascites, Cholestatic Liver Failure with AST and ALT > 5 UNL	Septic Shock in a context of liver failure

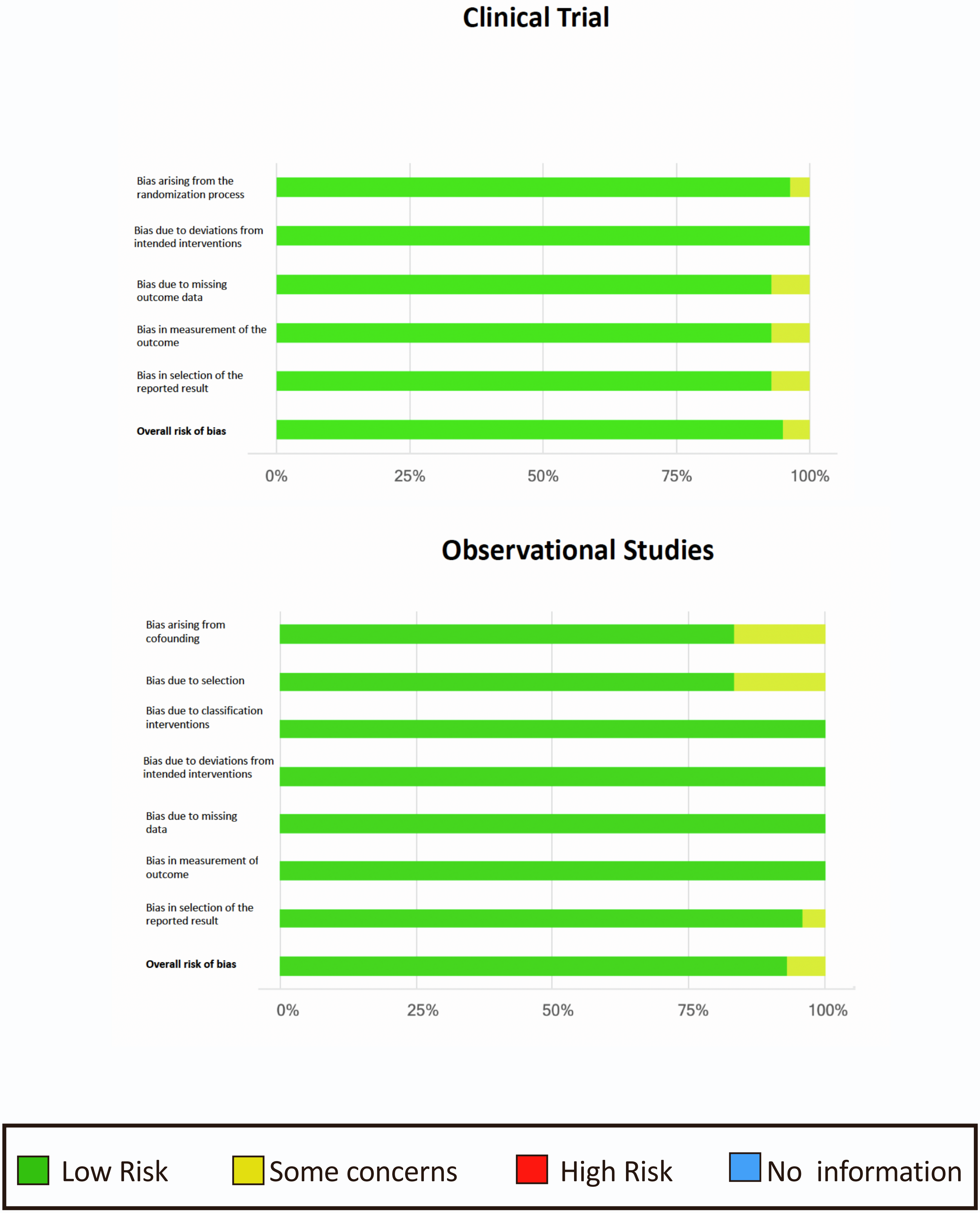

Figure S1. Synthesis of eligible clinical trial and of observational studies of risk of bias using the Cochrane risk-of-bias tool for randomized trials (RoB 1.0 and 2.0).

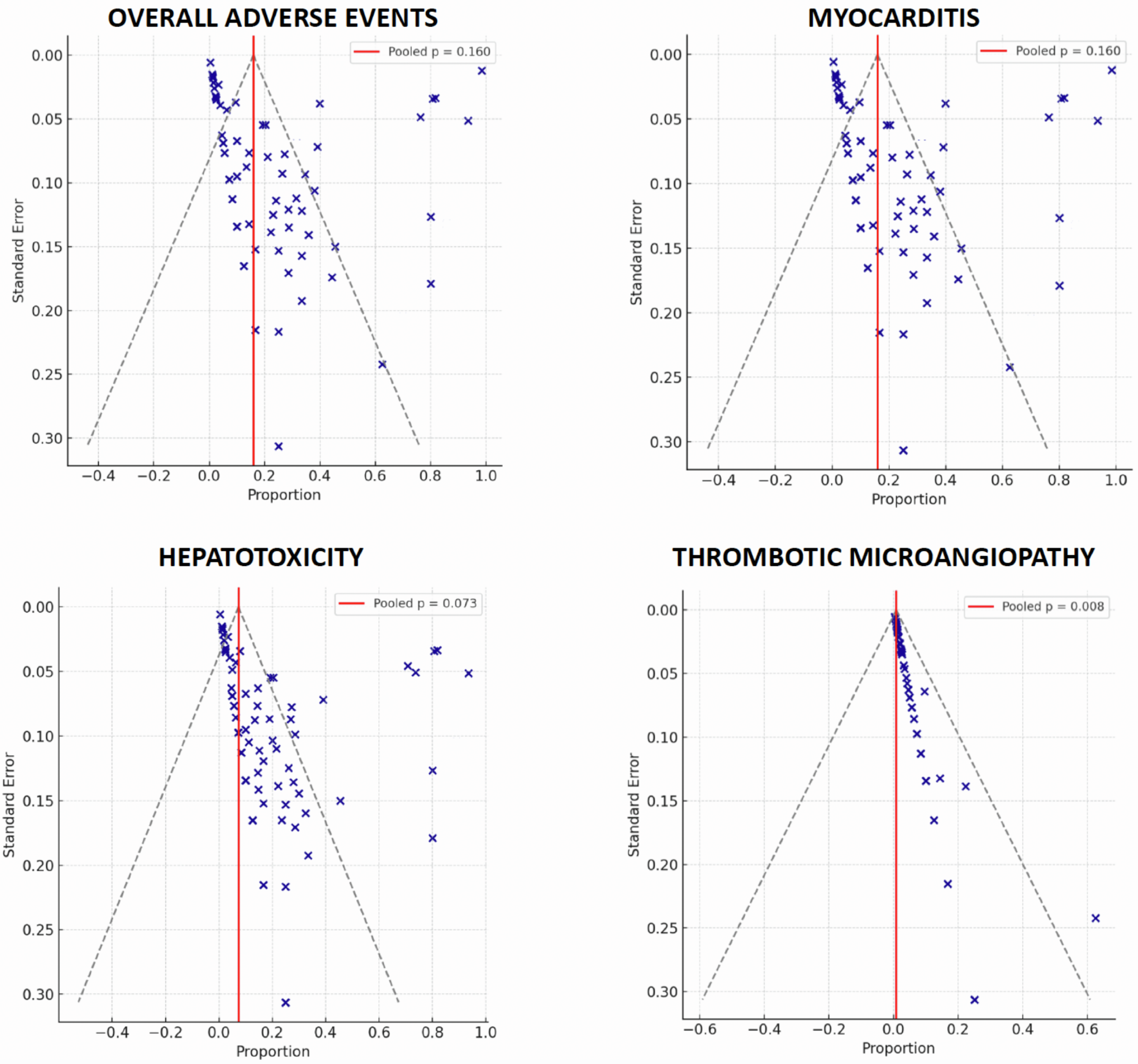

Figure S2 Funnel Plots for visual assessement of publication bias for the pooled incidence of Overall immune mediated adverse events (Top Left), Myocarditis (Top Right), Hepatotoxicity (Bottom Left) and Thrombotic Microangiopathy (Bottom Right).

Study	Year	Adverse Events	Patients	Incidence, %	95% CI, %	W, % (random)
Greenber et al.	2024	1	7	14.2	[2.5-51]	1.2
Hughes et al.	2022	0	6	0	[0-39]	1.1
Nakamura et al.	2023	0	32	0	[0-9.5]	1.3
Mendell et al.	2023	1	4	23	[12.4-35.7]	1
Zaidman et al.	2023	2	20	10.2	[2.8-30.1]	1.8
Belluscio et al.	2023	3	19	15.7	[2.7-64.3]	2.2
Dreghici et al.	2022	4	9	33.3	[12.1-64.5]	2.3
Laugel et al.	2024	1	3	33.3	[14.2-67.2]	1.2
Lek et al.	2023	1	1	100.0	[20.1-100]	0.1
Mendell et al.	2025	6	63	9.5	[4.4-19.2]	2.6
Flaning et al.	2022	0	2	0	[0-45.3]	1.1
Bowles et al.	2012	0	6	0	[0-42.1]	1.9
Mendell et al.	2010	0	6	0	[0-43.4]	1.9
Mendell et al.	2024	1	6	16.7	[3.5-56.5]	1.7
Smith et al.	2023	0	3	0	[0-46.3]	1.2
Smith et al.	2013	0	5	0	[0-44.1]	1.2
Corti et al.	2018	0	9	0	[0-38.4]	1.2
Strauss et al.	2022	5	15	33.3	[15.2-58.3]	2.5
Strauss et al.	2022	4	14	28.6	[11.8-54.7]	2.4
Mendell et al.	2017	2	15	13.3	[3.8-37.9]	2.2
Day et al.	2021	2	20	10	[2.8-30.1]	2.3
Merci et al.	2021	9	33	27.3	[15.1-44.2]	2.1
Goilou et al.	2022	1	1	100	[20.1-100]	1.1
Chand et al.	2021	3	3	100	[43-100]	1.2
Finekel et al.	2023	2	32	6.25	[1.7-20.1]	2.3
Gowda et al.	2022	99	99	100	[65-4-100]	1.3
Servais et al.	2024	67	168	39.8	[31.1-52.4]	2.1
Weib et al.	2022	58	76	76.7	[65.6-84.4]	2.2
Bitetti et al.	2023	6	9	66.7	[35.6-87.9]	2.1
Mendell et al.	2021	0	10	0	[0-38]	2.1
Favia et al.	2024	0	8	0	[0-32]	2.1
Waldrop et al.	2024	18	46	39.1	[26.4-53.5]	1.2
Waldrop et al.	2020	3	21	14.3	[4.9-34.6]	1.2
Chencheri et al.	2023	1	25	4	[0.7-19.5]	1.1
Pane et al.	2023	0	46	0	[0-19.4]	1.2
Tokaltly et al.	2023	25	25	100	[65.4-100]	3.1
Stettner et al.	2023	6	9	66.7	[35.4-87.9]	2.3
Matesanz et al.	2021	6	7	85.7	[48.7-97.4]	1.9
Gaber et al.	2021	7	9	77.8	[45.2-93.7]	2.2
D'Silva et al.	2022	8	21	38.1	[20.1-50.1]	2.1
Friese et al.	2021	2	9	25	[7.1-59.1]	2.1
Shieh et al.	2023	9	26	33.3	[18.6-52.1]	2.1
Ozelo et al.	2022	108	134	80	[72.5-85.1]	2.2
Ozelo et al.	2025	22	22	100	[51.4-100]	1.2
Manno et al.	2006	2	7	28.6	[28.2-61.1]	2.1
Pipe et al.	2023	11	54	20.3	[11.7-32.9]	2.1
Rangarajan et al.	2017	8	9	88.9	[56.5-98]	1.7
Mahlangu et al.	2024	108	132	80	[72.5-85.9]	1.4
Leavitt et al.	2024	5	11	45.5	[21.3-71.2]	1.3
Chapint et al.	2017	4	4	100	[65.3-100]	1.5
George et al.	2017	1	10	10	[1.8-40.4]	2.1
George et al.	2021	7	18	38.8	[23.4-56.7]	1.2
Nathwaani et al.	2011	2	6	33.3	[9.7-70]	1.2
Chowdary et al.	2022	8	10	80	[49.1-94.3]	1.2
Coppens et al.	2024	17	54	31.5	[23.2-51.3]	1.2
Cuker et al.	2024	28	45	62.2	[47.6-74.9]	1.2
Tai et al.	2022	0	26	0	[0-12.8]	1.2
Lv et al.	2024	0	6	0	[0-39.9]	1.2
Sevignz et al.	2024	0	19	0	[0-16.8]	1.2
D'Antigo et al.	2023	4	5	80	[47.5-96.3]	1.5
Tardieu et al.	2014	0	4	0	[9-48.9]	1.2
Tardieu et al.	2017	0	4	0	[0-48.9]	1.2
Deiva et al.	2021	0	4	0	[0-48.9]	1.2
Brunetti-Pierri et al.	2022	2	9	22.2	[6.3-54.7]	1.9
Mendell et al.	2015	0	6	0	[0-39.9]	1.2
Lyon et al.	2020	0	5	0	[0-25]	0.8
Hammond et al.	2016	0	42	0	[0-7.5]	0.8
Jasky et al.	2009	0	9	0	[0-21.3]	0.8
Jessup et al.	2011	0	39	0	[0-13.4]	0.8
Greenber et al.	2016	0	123	0	[0-6.7]	0.8
Grines et al.	2002	2	60	3.3	[0.9-11.4]	0.8
Grines et al.	2003	10	52	19.2	[10.8-31.9]	0.8
D'Avola et al.	2016	0	8	0	[0-16.6]	0.8
Ferreira et al.	2014	0	5	0	[0-25.5]	0.8
Weinstein et al.	2025	12	12	100	[67.8-100]	0.8
Flotte et al.	2022	0	2	0	[0-47.8]	0.8
Priddy et al.	2019	0	21	0	[0-6.8]	0.7
Flotte et al.	2011	0	9	0	[0-13.3]	0.7
		**Random Effects Model**	**1 908**	**30**	**[22.5-38.9]**	**100**
Heteogeneity: I^2= 83.1		τ^2 = 1.69	p<0.001			

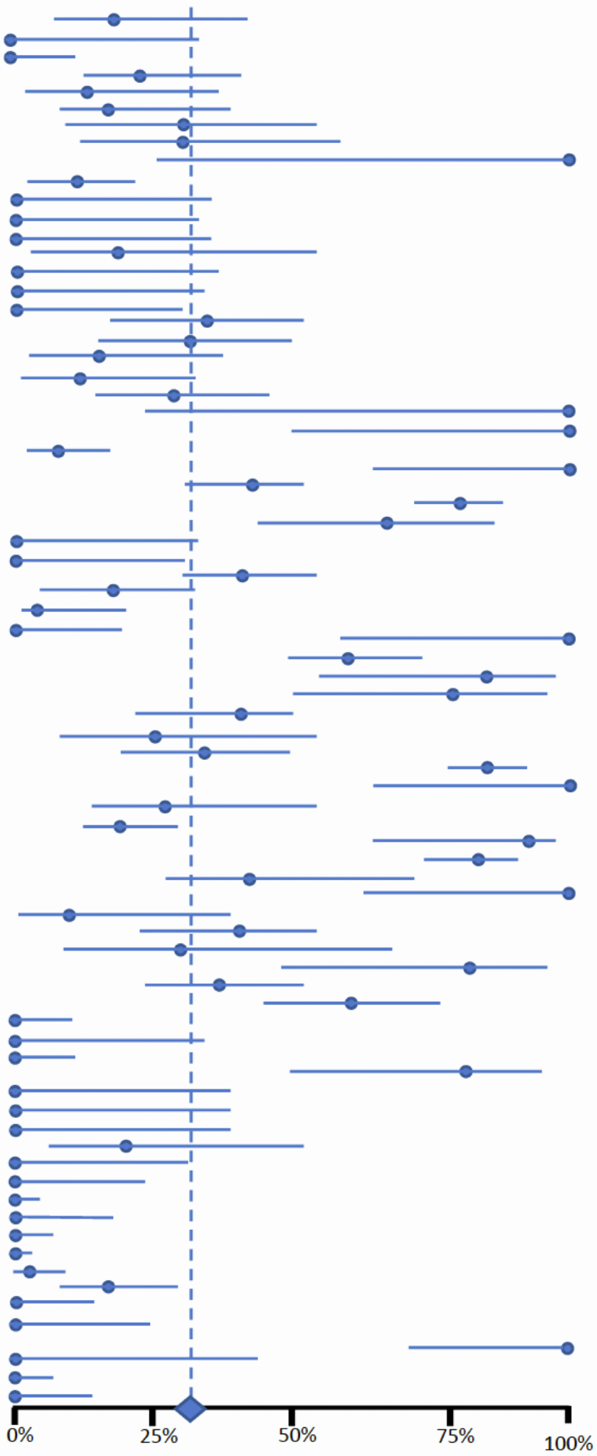

Figure S3 Forest Plot of incidence of immune mediated adverse events in AAV gene replacement therapies studies

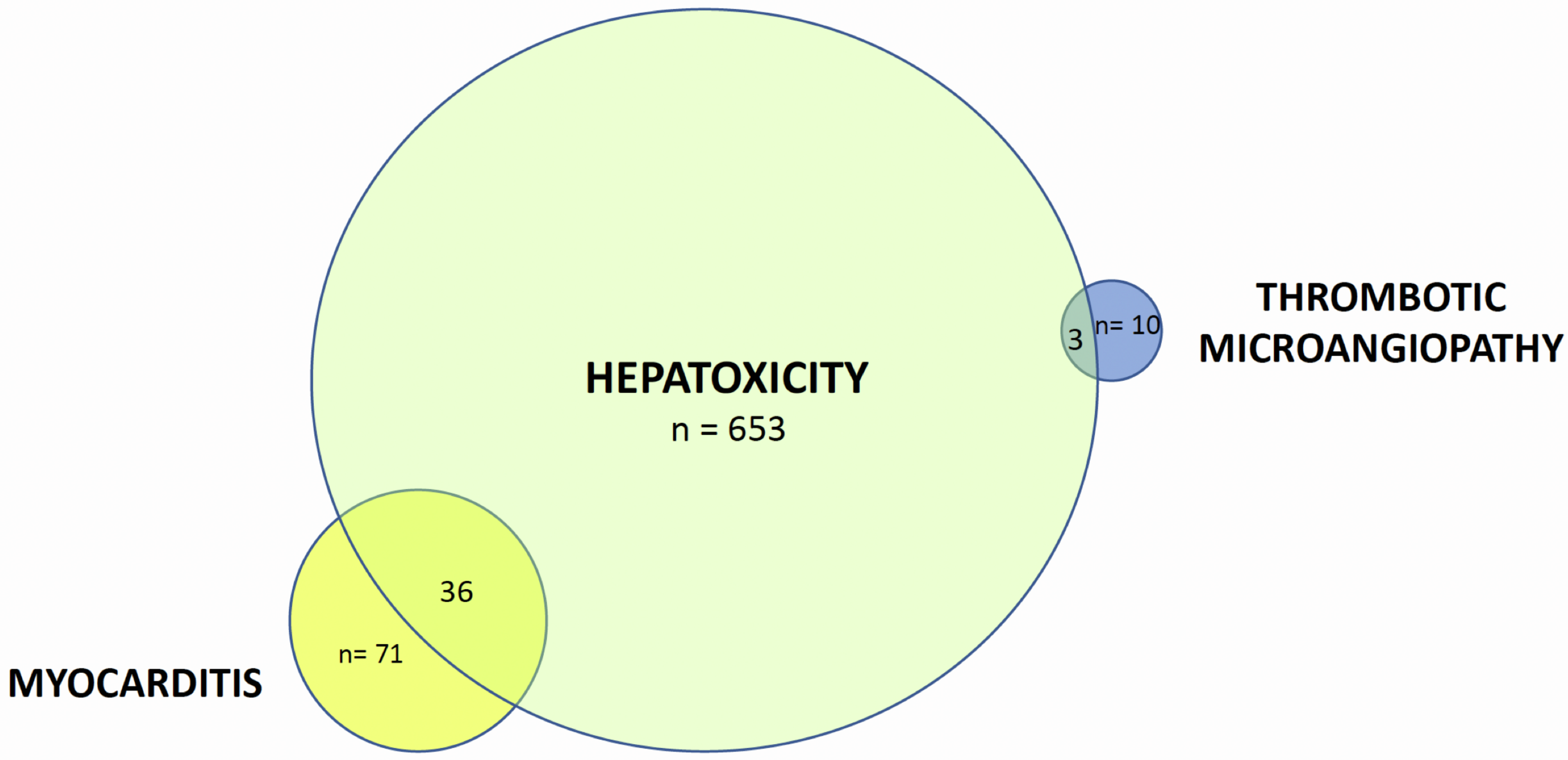

Figure S4 Overall occurrence and overlap of the 734 immune mediated AAV adverse events.

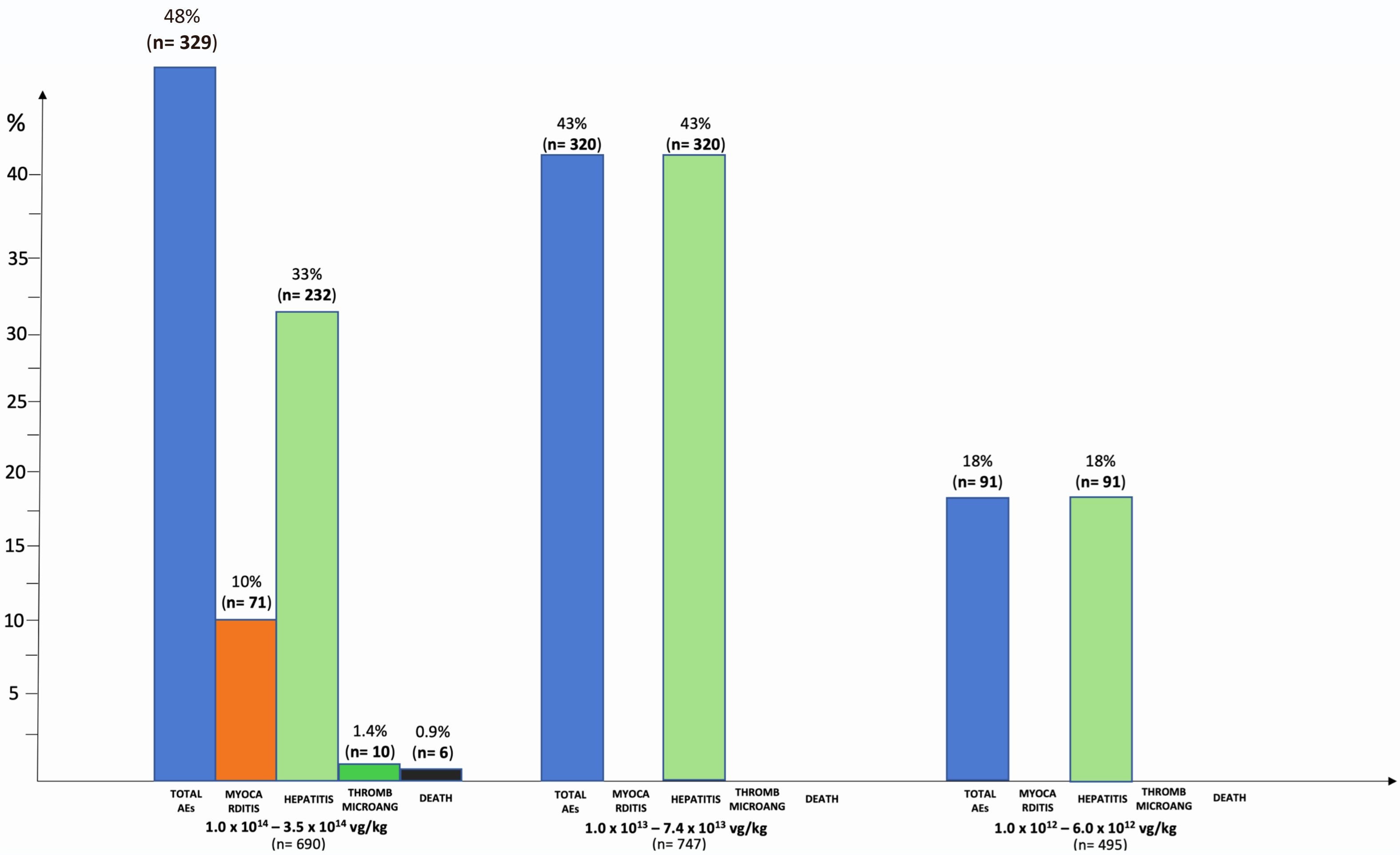

Figure S5 Prevalence of adverse events based on the infused dose.

Abbreviations: AEs: Adverse Events; THROM MICROANG: Thrombotic microangiopathy

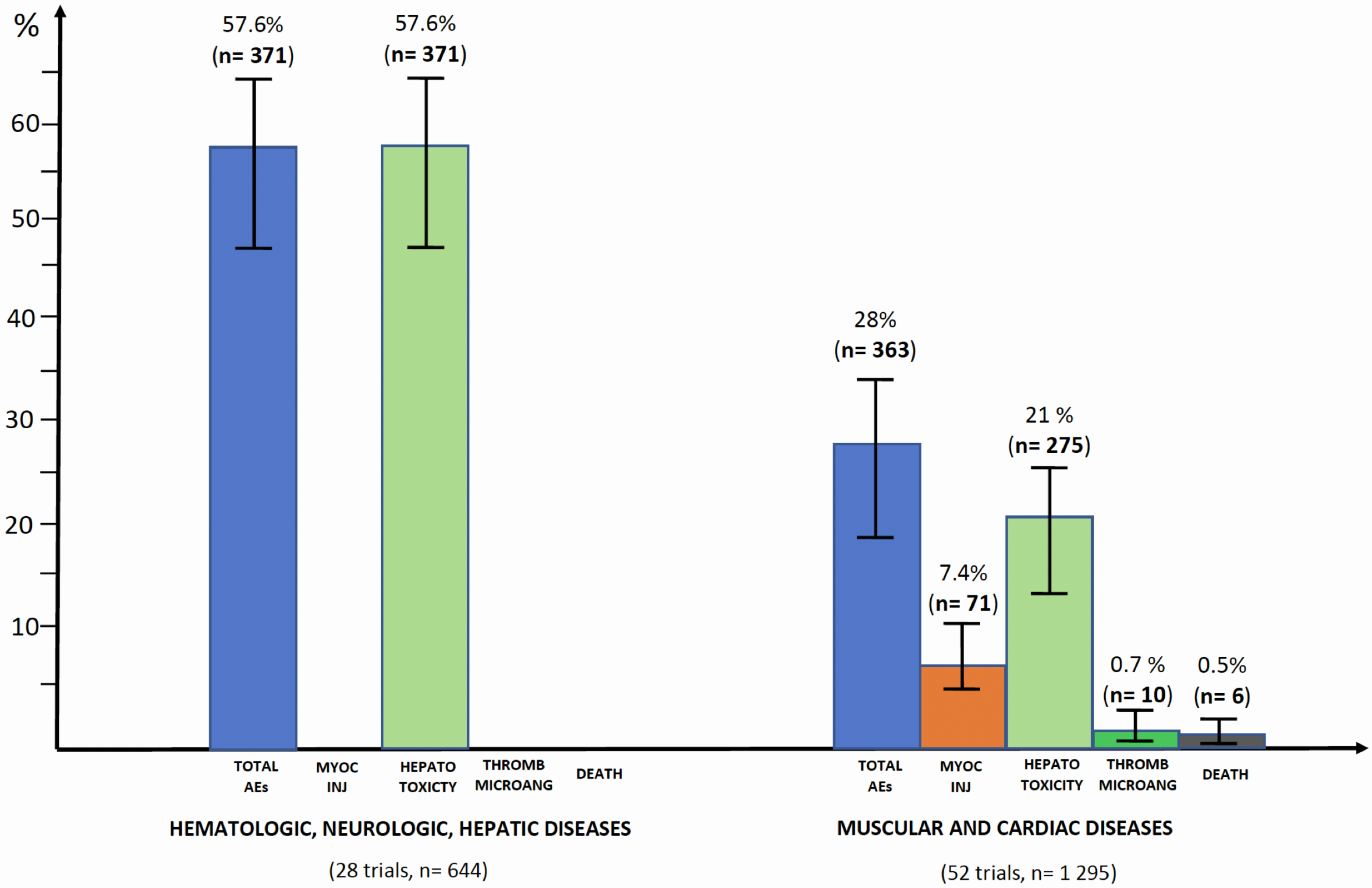

Figure S6 Prevalence of adverse events based on the type of treated disease.

Abbreviations: Aes: Adverse events; THROM MICROANG: Thrombotic microangiopathy
