# Supplementary material for: Incidence, timing, and clinical significance of adverse immune events after gene replacement therapy: A systematic review and meta-analysis
Source: Mol Ther. 2026 Jan 10;34(3):1340–51. doi: 10.1016/j.ymthe.2026.01.004 (PMC12974193; doi:10.1016/j.ymthe.2026.01.004)
Supplement: Document S2. Article plus supplemental information [file mmc2.pdf]

Molecular Therapy
Review

Incidence, timing, and clinical significance of adverse immune events after gene replacement therapy: A systematic review and meta-analysis

Niccolò Maurizi,[1,2] Enrico Ammirati,[3] Elizabeth Silver,[4] Kimberly Hong,[4] Quan Bui,[4] Alessia Argirò,[1] Iacopo Olivotto,[1,5] and Eric D. Adler[4]

[1]Cardiomyopathy Unit, Careggi University Hospital, Florence, Italy; [2]Service of Cardiology, University Hospital of Lausanne (CHUV) and University of Lausanne (Unil), Lausanne, Switzerland; [3]De Gasperis Cardio Center, Transplant Center, Niguarda Hospital, Milano, Italy; [4]Division of Cardiovascular Medicine, Department of Medicine, University of California, San Diego, San Diego, CA, USA; [5]Cardiology Unit, IRCCS Meyer's Children Hospital, Florence, Italy

Adeno-associated virus (AAV)-based gene replacement has emerged as a transformative platform for severe genetic disorders, yet immune-mediated adverse events (AEs) pose significant barriers to widespread clinical adoption. We performed a systematic review and meta-analysis of prospective and retrospective studies of AAV gene therapy published between January 2005 and March 2025 (PROSPERO CRD420251046546). Data from 801 studies encompassing 1,972 patients and 2,142 patient-years were pooled to estimate the incidence and clinical impact of immunotoxicity. Random-effects meta-analysis yielded a 30.0% (95% CI, 22.5–38.9; I^2 = 83.1%) overall AE rate, including hepatotoxicity in 23.8% (17.4–31.7; I^2 = 81.7%), myocarditis in 6.2% (4.6–8.1; I^2 = 46%), thrombotic microangiopathy (TMA) in 4.7% (4.4–6.5; I^2 = 18.5%), and treatment-related death in 4.7% (3.0–5.3; I^2 = 46.1%). Hepatotoxicity and myocarditis were generally mild (97% and 96% non-serious), whereas all TMA episodes carried substantial morbidity. Time course analyses revealed TMA clustered in week 1, myocarditis at week 2, and hepatotoxicity up to 6 months post-infusion. In individual-patient analyses, vector serotype and doses >1 × 10^{12} vg/kg significantly increased AE risk (OR = 5.59 [1.35–12.2], p = 0.018; OR = 2.31 [1.04–5.53], p = 0.041), whereas combined corticosteroid, anti-CD20, mTOR- and calcineurin inhibitor regimens were protective (OR = 0.67 [0.47–0.96], p = 0.040). At least five cases of TMA, one of myocarditis, and three deaths could not be included in the present analysis because these events were described in company statements. These findings underscore that one-third of AAV recipients experience immunotoxicity, predominantly early and mild, and support proactive immunosuppression and vector optimization to enhance safety.

INTRODUCTION

Gene therapy using adeno-associated viral (AAV) vectors has emerged as a transformative approach for treating a range of severe genetic disorders.[1–3] However, the prospect of widespread clinical adoption has been tempered by concerns over immune activation and related adverse events (AEs) such as hepatotoxicity, myocarditis, and thrombotic microangiopathy.[4–7] To date, our understanding of these complications has been derived exclusively from individual trial reports and small case-series or industry communications, leaving the incidence and clinical impact of immune-mediated AEs largely undefined.[8] Furthermore, because genetic therapies are often trialed in small numbers of patients, AEs at low or even medium frequencies may not be detected until more patients are treated. Characterizing the immunological responses associated with various gene therapy treatments would be crucial for optimizing treatment protocols, ensuring better safety and efficacy. Despite multiple studies documenting isolated incidents of AAV-associated immunotoxicity,[9] there has not been a comprehensive analysis that quantifies the overall incidence, characterizes clinical significance, or identifies factors associated with AEs. Therefore, this systematic review and meta-analysis aims to address this critical knowledge gap by synthetizing and characterizing AAV-based gene therapy AEs described in published studies and records from the International Pharmacovigilance Databases.

RESULTS

A total of 756 abstracts were screened and 182 full texts have been reviewed, resulting in 81 studies comprising 1,972 patients[23–102] (Figure 1). Details about included studies, treated disease, type of vector used, dose, and immunosuppressive regimen can be found in Tables 1 and S4. The majority of included studies were clinical trials (61, 76%), whereas 19 (24%) were observational studies. Most of the studies included <50 patients (70 [88%] studies) and dose ranged from 3.3 × 10^8 to 1.1 × 10^{14} vg/kg. Three types of immunosuppressive regimen were used: reactive corticosteroids (investigator administration of corticosteroids following a possible AE) (28 [35%] studies, 915 patients), pre- and post-injection corticosteroid therapy (42 [53%] studies, 968 patients), and pre- and post-injection corticosteroid therapy and/or mTOR inhibitors and/or calcineurin inhibitor and/or anti-CD-20 monoclonal antibodies (9 [12%] studies, 56

https://doi.org/10.1016/j.ymthe.2026.01.004.

Correspondence: Niccolò Maurizi, MD, Service of Cardiology, University Hospital of Lausanne (CHUV) and University of Lausanne (Unil), Lausanne, Switzerland.
E-mail: niccolo.maurizi@chuv.ch

 © 2026 The Author(s).
Published by Elsevier Inc. on behalf of The American Society of Gene and Cell Therapy.
This is an open access article under the CC BY license (http://creativecommons.org/licenses/by/4.0/).

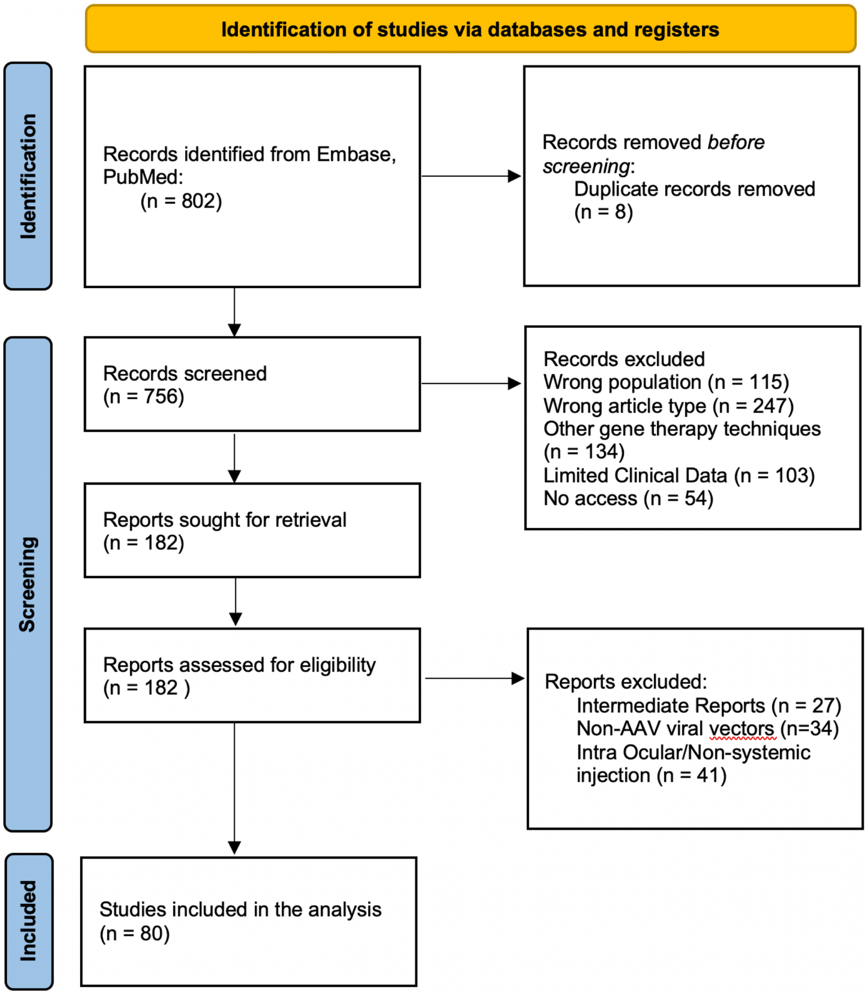

Figure 1. Preferred reporting items for systematic reviews and meta-analyses selection and analysis process of the reports included in the systematic review and meta-analysis

patients) (Table 1). One study did not report the immunosuppression regimen.[24] A total of 35/81 studies involved patients treated for spinal muscular atrophy (SMA) and Duchenne muscular dystrophy (DMD).

The risk for bias assessments showed that, for clinical trials, four (5%) were at moderate risk of bias for outcome measure, four (5%) at unclear risk for selective reporting, with an overall risk of moderate bias of 3%. For observational studies, four (17%) presented some concerns in the patient selection, four (17%) in confounding, with an overall risk of bias of 8% (Tables S5 and S6; Figure S1); funnel plots are presented in Figure S2. Missing data per study are reported in Table S7. A random-effects meta-regression on the logit-transformed incidence proportions formally tested that the incidence of AEs did not differ between clinical trials and observational studies ($\beta = -0.580$, OR = 0.51 [0.24–1.12], $p = 0.081$) (Table S5).

Pooled incidence of immune-mediated AEs

A total of 734 AEs were reported over 2,152 patient-years of pooled observation. Hepatotoxicity occurred in 653 patients, followed by myocarditis in 71 and TMA in 10. Six treatment-related deaths have been reported. A total of 31 (39%) studies did not report any AE, whereas 9 (11%) presented an AE in more than 80% of the patients included (Figure S3).

Pooled incidences were, respectively: 30% (95% CI, 22.5%–38.9%; $I^2 = 83.1\%$, $p < 0.01$) for all AEs, 23.8% (95% CI, 17.4%–31.7%; $I^2 = 81.7\%$, $p < 0.01$) for the occurrence of hepatotoxicity, 6.2% (95% CI, 4.6%–8.1%; $I^2 = 46\%$, $p = 0.07$) for myocardial injury/myocarditis, 4.73% (95% CI, 4.4%–6.5%; $I^2 = 18.5\%$, $p = 0.083$) for TMA and 4.7% (95% CI, 3%–5.3%; $I^2 = 46.1\%$, $p = 0.809$) for treatment-related death (Figure 2). Pooled incidence rates per 100 patient-years were: 33.8 (95% CI, 23.4–43.1%; $I^2 = 46.1\%$, $p < 0.01$) for all AEs, 28.6% (95% CI, 19.9%–36.7%; $I^2 = 82.6\%$, $p < 0.01$) for hepatotoxicity, 8.6% (95% CI, 5.8%–10.7%; $I^2 = 63.2\%$, $p = 0.13$) for myocardial injury/myocarditis, and 7.1% (95% CI, 4.7%–10.7%; $I^2 = 52.8\%$, $p = 0.065$) for TMA (Figure 2).

Timing and clinical significance of immune-mediated Aes

TMA occurred mostly at week 1 after treatment (60.2% [95% CI, 57.2%–62.9%; $I^2 = 47.5\%$]), whereas myocardial injury/myocarditis peaked at week 2 (57.9% [95% CI, 55.3%–62.4%; $I^2 = 16.1\%$]). No cases of myocardial injury/myocarditis or TMA were reported after the first month post-injection (Figure 3A). Hepatotoxicity had a median time of onset of 38 days (IQR = 19–67 days) and occurred up to 6 months after injection (24.2% [95% CI, 22.1%–28.8%; $I^2 = 74.1\%$]).

Of the 71 cases of myocarditis, in 36 (51%) patients it presented with concomitant hepatotoxicity[26,28,32,35,42,49–52,59,60,65] (Figure S4). The clinical picture involved mild elevation of cardiac enzymes without echocardiographic changes (68/71, 94%) (Table S8). In the four cases who had clinical consequences, all but one resolved completely at the end of the study period (Figure 3B).[26,28,35] Hepatotoxicity was the most common AE, reported in 653 patients, consisting in an asymptomatic elevation of liver function tests in 633 (97%). In 16 (4%) patients, it progressed to transient hepatic failure. TMA was rare, occurring in 10 patients, but all had serious clinical consequences requiring hospitalization (Figure 2B).

Table 1. Overview of the studies included by the target disease

Disease treated	No. of studies	Sample size <10	10–50	>50	Vector used	Doses	Immunosuppressive regimen: Reactive corticosteroids	Pre- + post-treatment corticosteroids	Pre- + post-treatment corticosteroids + mTOR inhib and/or calcineurin inhib and/or anti-CD-20
Danon[23]	1	1	0	0	AAV-9	6.7×10^{13} to 1.1×10^{14} vg/kg	0	0	1
Anderson-Fabry[24]	1	1	0	0	AAV-2	1×10^{13} to 5×10^{13} vg/kg	0	1	0
Duchenne muscular dystrophy[25–35]	11	8	2	1	AAV-8, AAV-9, rAAVrh74	1×10^{12} to 1×10^{14} vg/kg	1	6	2
Limb-girdle muscular dystrophy 2B[36,37]	2	2	0	0	rAAV-1, rAAVrh74	1×10^{11} to 7.4×10^{13} vg/kg	1	1	0
Pompe[38–40]	3	3	0	0	rAAV, AAV-8, AAV-1	1×10^{12} to 5×10^{12} vg/kg	1	1	1
Spinal muscular atrophy[41–64]	24	8	13	3	scAAV9-FL-SMNcDNA	6.7×10^{13} to 1.1×10^{14} vg/kg	0	24	0
X-linked myotubular myopathy[65]	1	0	1	0	AAV-8	1.3×10^{14} to 3.5×10^{14} vg/kg	0	1	0
Hemophilia A[66–72]	7	1	4	2	AAV-3, AAV-5, rAAV-6	9×10^{11} to 6×10^{13} vg/kg	5	2	0
Hemophilia B[73–79]	7	2	3	2	rAAV-2, AAV-5, AAV-8, AAV-2, AAVs3	8×10^{10} to 2×10^{13} vg/kg	5	2	0
AADC deficiency[80]	1	0	1	0	AAV-2	1.8×10^{11} to 2.4×10^{13} vg/kg	1	0	0
Autosomal recessive deafness[81]	1	1	0	0	AAV-1	9×10^{11} to 1.5×10^{12} vg/kg	1	0	0
Frontotemporal dementia[82]	1	0	1	0	AAV-9	2.1×10^{13} to 4.2×10^{13} vg/kg	0	0	1
Crigler-Najar[83]	1	1	0	0	AAV-8	2×10^{12} to 5×10^{12} vg/kg	0	0	1
Mucoplysaccharidosis type IIIA/IIB/IV[84–87]	4	4	0	0	AAV-2/5, AAV-8, rAAV	6×10^{11} to 6×10^{12} vg/kg	0	1	3
Becker muscular dystrophy[88]	1	1	0	0	AAV-1	3×10^{11} to 6×10^{13} vg/kg	0	1	0
Chronic heart failure[89–93]	5	2	2	1	AAV-1, Ad5-FDG	1.4×10^{11} to 1×10^{13} vg/kg	5	0	0
Stable angina pectoris[94–96]	4	0	4	0	Ad5-FDG	3.3×10^{8} to 1×10^{11} vg/kg	4	0	0
Acute intermittent porphyria[97]	1	1	0	0	rAAV-2/5	5×10^{11} to 1.8×10^{13} vg/kg	1	0	0
Lipoprotein lipase deficiency[98]	1	1	0	0	AAV-1	1×10^{12} to 5×10^{12} vg/kg	0	1	0
Glycogen storage disease type Ia[99]	1	0	1	0	AAV-2	2×10^{12} to 6×10^{13} vg/kg	1	1	0
Tay-Sachs[100]	1	1	0	0	rAAVrh8	1×10^{13} vg/Kg	0	1	0
HIV[101]	1	0	1	0	AVV-1	1×10^{12} to 1×10^{14} vg/kg	1	0	0
AAT deficiency[102]	1	0	1	0	rAAV, AAV-2	0.5×10^{13} to 5×10^{13} vg/kg	0	1	0

AAV, adenovirus; AAT, α-1 antitrypsin.

A total of six deaths have been reported.[32,46,65] Case 1[32] was a 27-year-old male with DMD treated with rAAV-9 using a dead *Staphylococcus aureus* Cas9 at high dose. The patient had restrictive pulmonary defect, severe reduced muscle mass with severe muscle wasting, and a compensated cardiomyopathy. He experienced a cytokine-mediated capillary leak syndrome with consequent cardiac dysfunction, related to an acute effect of the AAV gene therapy. Concomitant myocarditis was possible, but was likely not the

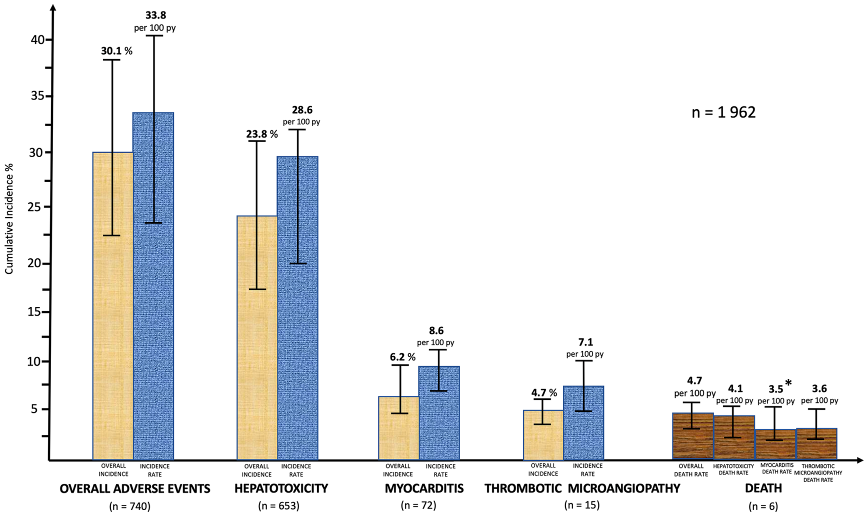

Figure 2. Pooled and annual incidence of immune adverse events and death after AAV gene replacement therapy

Pooled incidence of adverse events, hepatotoxicity, myocarditis, and thrombotic microangiopathy cases are presented with their 95% confidence intervals.
py, patient-years. *Episode of death occurred in the context of a cytokine-mediated capillary leak syndrome with an associated cardiac dysfunction due to treatment acute toxic effect on a background of a pre-existing cardiomyopathy. Concomitant myocarditis was possible, but did not probably represented the direct cause of death..

primary cause of death. Case 2[46] occurred in a 4-month-old patient affected by SMA, treated with high dose scAAV-9, carrying a variant in the complement factor 1 gene (probably partially responsible for the severity of the reaction) who developed the first week after injection a severe clinical picture of TMA leading to multi-organ failure day 30 after injection. The remaining four cases[65] were reported in patients from 2 to 6 years old treated with AAV-8 gene replacement for X-linked myotubular myopathy (XLMTM) due to liver failure. Although baseline transaminases and bilirubin were within protocol limits, subsequent clinical and histopathologic evaluations in several XLMTM patients revealed pre-existing hepatobiliary structural abnormalities that likely increased susceptibility to AAV-associated hepatotoxicity[65] (Table S9).

Factors associated with occurrence of immune-mediated AEs

Hepatotoxicity occurred only in patients with hematologic, neurologic, or hepatic diseases, whereas myocardial injury/myocarditis and TMA were reported only in patients with muscular and cardiac diseases (Figure S6).

AAV-5, AAV-6, AVV-2/8 and AAV-8, AAV-9, and recombinant AAVs increased the risk of AEs (OR = 5.59 [1.35–12.2], p = 0.018 and OR = 3.96 [1.01–12.2], p = 0.04), as well as a dose $>10^{12}$ vg/kg (OR = 2.31 [1.04–5.53], p = 0.04) (Table 2). Aggressive immunosuppressive regimen (pre- or post-treatment corticosteroids with mTOR inhibitor and/or calcineurin inhibitors and/or anti-CD-20) showed protection against AEs (OR = 0.67 [0.07–0.96], p = 0.04) (Figure 4).

Global pharmacovigilance analysis

A total of 2,134 and 2,197 AEs have been reported in VigiAccess and FDA Adverse Event Reporting System (FAERS) Databases, respectively, for 5 commercially available gene replacement drugs (Table 3). Immune-mediated AEs were 262/2,134 (12%) and 448/2,197 (20%). Hepatotoxicity was the most common reported AEs for all four drugs in all databases. Myocarditis occurred in relation to delandistrogene moxeparvovec (1/16 [6%] in VigiAccess, 11/29 [38%] in FAERDS) and onasemnogene abeparvovec (14/217 [6%] in VigiAccess, 118/395 [30%] in FAERDS, although for the latter it is not known whether a baseline increase of the troponin was present).

TMA cases (43/217, 20%) were reported only in VigiAccess in relation to onasemnogene abeparvovec. No AEs were present for eldocagene exuparvovec (Table 3).

DISCUSSION

The widespread clinical adoption of gene replacement therapy has been tempered by concerns over immune activation.[5–7] To date, the understanding of these complications has been limited and derived exclusively from individual trial reports, small case-series, or company communications.[8] We report here the first comprehensive analysis to our knowledge of the incidence and clinical significance of immune-mediated AEs after gene replacement therapy. We analyzed 80 studies encompassing 1,939 patients treated over a total of 2,122 patient-years. Overall, one-third of patients experienced at least one immune-mediated AE (pooled incidence 30%; 95% CI, 22.5%–38.9%; I^2 = 83.1%). Hepatotoxicity was the most prevalent complication (pooled incidence 23.8%), followed by myocarditis (6.2%) and TMA (pooled incidence 4.7%). Time-to-onset differed by AE type: TMA generally emerged within the first week, myocarditis peaked in week 2, and hepatotoxicity could manifest up to 6 months post-infusion. While AAV-mediated immunotoxicity is relatively common, most events cluster in the early post-treatment period and are transient or mild in severity.[103]

Timing and clinical significance of immune-mediated AEs

Clinically, the majority of AEs were mild and required minimal intervention. Of 653 hepatotoxicity events, 97% were asymptomatic liver function test elevations; only 16 cases (4%) progressed to transient hepatic failure, resolved by study end. Importantly, several of the most severe hepatobiliary events occurred in the context of pre-existing hepatobiliary vulnerability (histopathologic or developmental biliary anomalies), not always apparent on routine baseline transaminase testing.[64] Thus, while transaminase elevations were

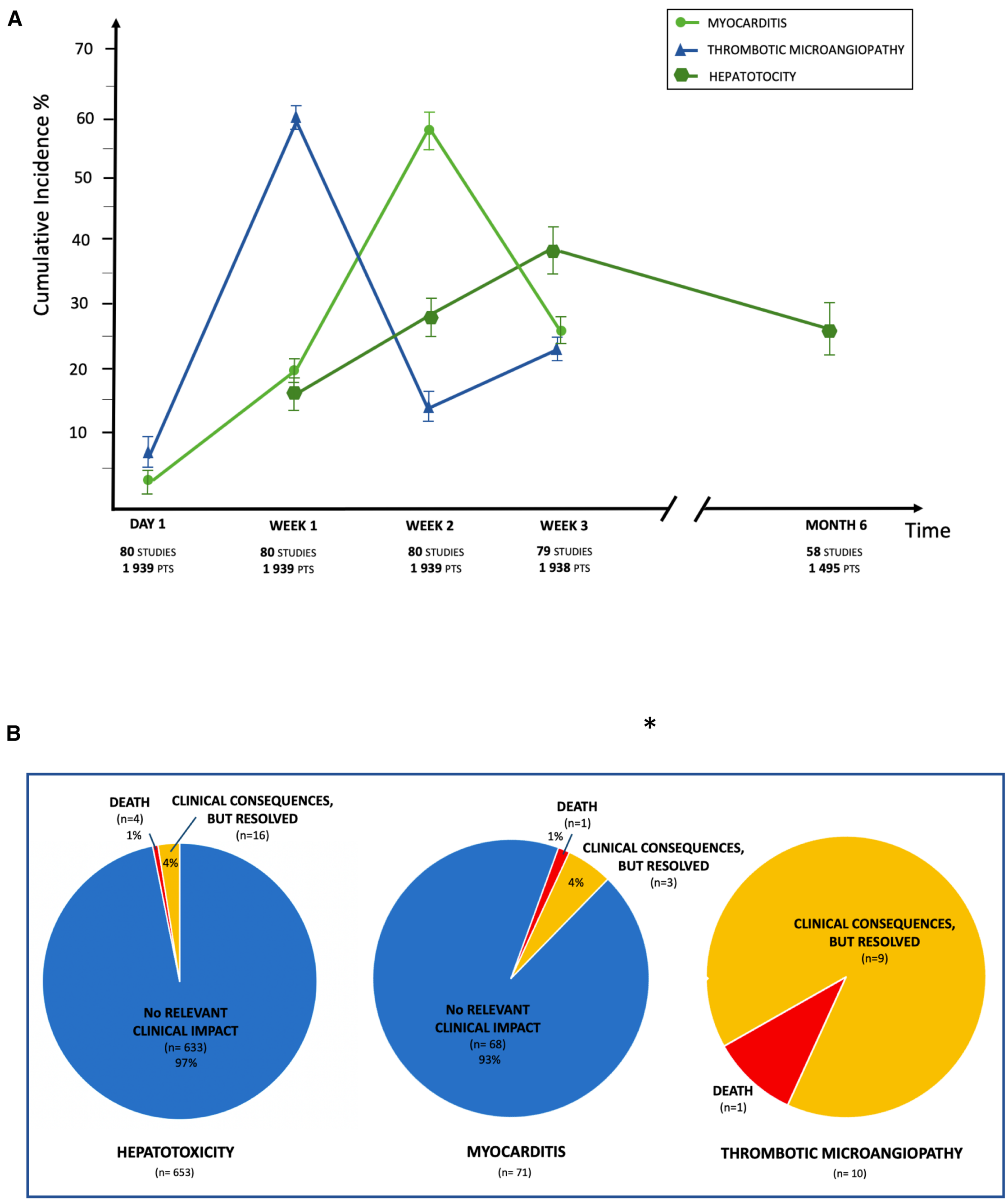

Figure 3. Timing of onset and clinical impact of gene replacement-related myocarditis, thrombotic microangiopathy, and hepatitis
(A) The timing of onset of myocarditis, hepatitis, and thrombotic microangiopathy is presented. For each time point, the cumulative incidence is displayed, with the 95% confidence intervals. (B) The clinical significance for adverse events is shown: from no relevant clinical impact, with clinical consequences but resolved in the follow-up or if it resulted in death. *Episode of death occurred in the context of a cytokine-mediated capillary leak syndrome with an associated cardiac dysfunction due to treatment acute toxic effect on a background of a pre-existing cardiomyopathy. Concomitant myocarditis was possible, but did not probably represent the direct cause of death.

common, they were not uniformly predictive of liver failure. A major limitation of the current literature is inconsistent reporting of liver-synthetic and cholestatic indices. These parameters were not available in a standardized fashion across studies and therefore could not be systematically analyzed. Finally, although the majority of hepatotoxic events clustered within the first 4–12 weeks, a minority of reports described new liver abnormalities up to 6 months after dosing. Potential explanations for delayed presentations include

Table 2. Predictors of adverse to immune activation after gene replacement therapy

Variable	Category	Immune-mediated adverse events (n = 734)	OR (95% CI)	p values
Vector class	high pre-existing population immunogenicity (Ad5-FGF5; AAV-1; AAV-2/5)	14/367 (4%)	–	–
	intermediate pre-existing population immunogenicity (AAV-5; AAV-6)	361/678 (53%)	5.59 [1.35–12.2]	0.018
	low pre-existing population immunogenicity (AAV-8; AAV-9, recombinants AAV)	373/894 (42%)	3.96 [1.01–9.7]	0.04
Dose	≤10 (12) vg/kg	91/495 (18%)	–	–
	10 (13) to 10 (14) vg/kg	648/1 444 (45%)	2.31 (1.04–5.53)	0.041
Immunosuppression	reactive corticosteroids only	292/915 (32%)	–	–
	pre- + post-treatment corticosteroids	437/968 (45%)	0.8 (0.6–1.49)	0.164
	pre- + post-treatment corticosteroids + mTOR inhib and/or calcineurin inhib and/or anti-cd-20	6/56 (14%)	0.67 (0.07–0.96)	0.04

For each variable, a clinically relevant comparison group have been chosen. For vector class, for doses the odds ratio of 10^{13} to 10^{14} vg/kg with respect to $\leq 10^{12}$ vg/kg are provided. For immunosuppression regimen, reactive corticosteroids are compared with more intense immunosuppressive regimens and the odds ratios are presented.
AAV, adenovirus; OR, odds ratio; mTOR Inhib, mammalian target of rapamycin inhibitors.

protracted immune activation, evolving cholestatic processes in predisposed individuals, drug interactions unmasking subclinical disease, or delayed recovery from earlier subclinical injury.[7]

Myocarditis and myocardial injury was reported in 71 patients, mostly (96%) presenting with mild troponin elevations without left ventricular dysfunction or wall motion abnormalities.[42,49–52,59,60,65] Four required brief hospitalization and all but one fully recovered.[32] Early (week 1–2) troponin elevations, often with systemic TMA or complement features are compatible with innate and complement-driven endothelial injury (C3a/C5a-mediated activation), whereas later events are more consistent with adaptive, cytotoxic T cell responses against the capsid or transgene product. Complement cleavage products (C3a, C5a), which are potent endothelial activators and implicated in AAV-associated TMA, might be useful in monitoring algorithms to differentiate early innate events from delayed cellular myocarditis.[4] No cases of myocarditis/myocardial injury or TMA were reported beyond 1 month post-infusion. In contrast, TMA, although infrequent (n = 10), was uniformly severe, necessitating hospitalization and advanced supporting care. Six deaths related to AAV therapy were documented. These fatal cases were characterized by high vector doses, underlying organ-specific vulnerability (e.g., pre-existing liver disease), and early onset (<8 weeks), suggesting that patient selection (e.g., baseline organ function) and more conservative dosing may be crucial in selected cases for preventing lethal outcomes.

We recommend systematic reporting of AE with rigorous scientific studies. At least five cases of TMA, one of myocarditis, and three deaths could not be included in the present analysis because these events were described in company statements.[104–108] The impact on the pooled incidence would have not been substantial, but the absence of transparent and scientifically rigorous reporting are missed opportunities to improve the understanding of the underlying pathophysiologic mechanisms and their prevention.

Factors associated with immune-mediated AEs

Vector serotype and dose were associated with AEs. Specifically, certain serotypes conferred a more than 5-fold increase in AE risk and higher doses (>1×10^{12} vg/kg) doubled the risk (Figure S5), underscoring the need to balance effective transgene expression with immunological safety. Conversely, regimens incorporating both pre- and post-infusion corticosteroids and mTOR inhibitors and/or calcineurin inhibitors and/or anti-CD-20 monoclonal antibodies reduced AE risk by 33% (despite use in fewer studies, resulting in a small sample size). However, because these agents differ fundamentally in their targets, the pooled estimates should be interpreted as hypothesis-generating only. Yet, the underlying immunopathogenic pathways remain incompletely elucidated.[4] For instance, TMA appears to be mediated by complement activation in the presence of high anti-capsid antibody titers,[6] yet the precise sequence of endothelial injury and platelet consumption is poorly characterized. There is a critical need for mechanistic studies, both in preclinical models and in early-phase trials, to systematically evaluate cytokine profiles, complement activation markers, endothelial injury assays, and histological characterization, alongside pharmacokinetic and pharmacodynamic parameters.[4,109] Such investigations should also test whether tailored immunosuppressive regimens (e.g., complement inhibitors, transient B cell depletion) can interrupt these pathways without blunting transgene expression or excessively immunosuppressing the patient.

Pharmacovigilance analysis

Our analysis of two major pharmacovigilance databases corroborates the toxicity profile observed in clinical trials. Immune-mediated events comprised 12%–20% of AEs reported for the five commercially approved AAV therapies. Hepatotoxicity remained the most common AE, while myocarditis was reported in association with

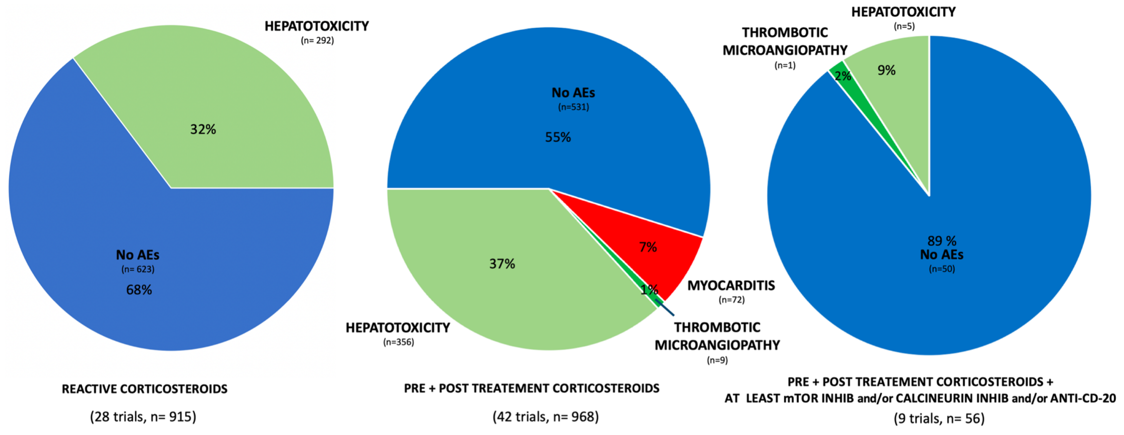

Figure 4. Incidence of adverse events based on the immunosuppressive regimen
Inhib, inhibitors.

delandistrogene moxeparvovec (6% in VigiBase; 38% in FAERS) and onasemnogene abeparvovec (6% in VigiBase; 30% in FAERS), although baseline troponin data were inconsistently documented in the latter. TMA represented 20% of all AEs for onasemnogene abeparvovec in VigiBase but was not captured in FAERS, likely reflecting underreporting or differences in case-definition adjudication. The alignment of real-world pharmacovigilance data with our pooled trial results reinforces the external validity of our findings and highlights that, even in less-controlled settings, immune-mediated AEs follow a similar distribution by organ system and severity.

Limitations of the study

This analysis is limited by underlying study quality. Considerable proportions of studies had limited patient numbers and moderate risks for bias. By design, substantial interstudy heterogeneity exists regarding the inclusion criteria and more than 40% of studies available were analyzing patients treated for DMD and SMA. Accordingly, the analysis employed random-effects meta-analyses applying the method of Hartung, Knapp, Sidik, and Jonkman to better account for interstudy variance and conducted meta-regression to explore the heterogeneity in the magnitude of associations. Small-study effects, including publication bias, may have resulted in the overestimation of the pooled incidences, particularly for total number of AEs and hepatotoxicity. Our reliance on published aggregate data and the construction of a pseudo-individual participant dataset precludes adjustment for key patient-level confounders such as baseline disease severity and concomitant medications and may not reflect causal relationship. Moreover, variability in follow-up duration and AE surveillance windows across studies may have led to underestimation of late-onset or subclinical immunotoxicity. Pharmacovigilance database analyses are limited by passive reporting and lack of detailed clinical adjudication, which limit conclusions about real-world immunotoxicity profiles. Lastly, we acknowledge that, while drafting this analysis, two deaths occurred. The first patient, participating in the phase 2 trial of RP-A501[107] complications related to a capillary leak syndrome and the concomitant use of a novel immune suppression agent in the pre-treatment regimen, implemented to mitigate complement activation observed in a phase I study. On May 23, 2025, the FDA placed a clinical hold on the trial.[108] The second death occurred in a patient with DMD treated with delandistrogene moxeparvovec-rokl following complications from acute liver failure, treated with pre- and post-injection corticosteroids.[108]

Conclusions

AAV gene therapy is associated with a 30% pooled incidence of immune-mediated AE, mostly occurring early and clinically mild and transient. Hepatotoxicity is the most commonly reported, while myocarditis and TMA remain less frequent but warrant vigilant monitoring due to potential severity. Vector serotype and high dose are associated with AEs, whereas robust peri-infusion immunosuppression might mitigate this risk. Fatalities, although rare, usually occur in the context of high vector burden and pre-existing organ compromise, underscoring the importance of careful patient selection and dosing. Future efforts should focus on elucidating the immune pathophysiology of these events and on prospectively evaluating targeted immunomodulatory strategies. Overall, our data demonstrate the safety of gene therapy for the majority of patients enrolled in published trials, but that further innovation is required to fulfill its promise. Real-world pharmacovigilance data paralleled that of trial-derived safety signals.

MATERIALS AND METHODS

This meta-analysis is reported in line with the Meta-analyses Of Observational Studies in Epidemiology[10] (Table S1) guidance and the Preferred Reporting Items for Systematic Reviews and Meta-Analyses (Table S2)[11] statements and was registered on the international Prospective Register of Systematic Reviews (CRD420251046546).[12]

Search strategy

The electronic databases MEDLINE, Embase, and Pubmed were comprehensively searched for English language papers from January

Table 3. Real-world immune adverse events as reported in the VigiAccess and FAERS Database in commercially available gene replacement treatments

		Delandistrogene moxeparvovec	Onasemnogene abeparvovec	Etranacogene dezaparvovec	Valoctocogene roxaparvovec	Eladocagene exuparvovec
Vector		rAAVeh74	AAV-9	AAV-5	AAV-5	AAV2-hRPE65v2
Disease		DMD	SMA	Hem B	Hem A	AADC deficiency
Patients treated		≅ 800	≅ 4,000	N/A	N/A	N/A
Overall reported AEs	VigiAccess	101	1,969	23	20	21
	FAERS	122	2,012	22	34	6
Total immune-mediated AEs	VigiAccess	16/101 (16%)	217/1,969 (11%)	18/23 (78%)	11/20 (55%)	0
	FAERS	29/122 (24%)	395/2,012 (20%)	5/22 (23%)	19/34 (56%)	0
Hepatotoxicity	VigiAccess	15/16 (94%)	160/217 (74%)	18 (100%)	11 (100%)	0
	FAERS	18/29 (62%)	277/395 (70%)	5 (100%)	19 (100%)	0
Myocarditis	VigiAccess	1/16 (6%)	14/217 (6%)	0	0	0
	FAERS	11/29 (38%)	118/395 (30%)	0	0	0
Thrombotic microangiopathy	VigiAccess	0	43/217 (21%)	0	0	0
	FAERS	0	0	0	0	0

AEs, adverse events; DMD, Duchenne muscular dystrophy; FAERS, FDA Adverse Event Reporting System.

2005 through March 2025. The search syntax was designed for prospective and retrospective studies including patients treated with AAV gene replacement therapy and detailed in Table S3. Ongoing trial registry records were excluded. Any discrepancies in study selection were resolved through discussion and consensus between the two reviewers (N.M. and E.S.; Figure S1). A third reviewer (K.H.) was available for arbitration. If key data were missing from the published report, we planned to contact the corresponding authors for clarification. If these data remained unavailable after inquiry/no contact was possible, the study was included in analyses for which it provided sufficient data.

Data extraction and quality assessment

Full text review and prespecified item extraction such as number of patients, disease treated, observation time, type of vector used, dose, immunosuppressive regimen, type, and clinical significance of the AE was performed independently at the study level by two independent reviewers (N.M. and E.S.). Quality assessment was performed using the Cochrane Risk of Bias tool for Clinical Trial and Observational Studies.[13,14]

Outcomes

The primary outcome of interest was to assess the incidence of AAV gene replacement therapy immune-mediated AEs. Specifically, acute myocarditis was defined by the presence of cardiac symptoms (e.g., chest pain, dyspnea, palpitations, syncope), an elevated cardiac troponin (cTn) above the 99th percentile, and abnormal electrocardiographic and/or echocardiographic and/or Cardiac Magnetic Resonance, and/or histopathologic findings on biopsy or postmortem evaluation in the absence of flow-limiting coronary artery disease.[15] Myocardial injury was defined as a condition defined by a cTn level above the 99th percentile upper reference limit.[15] In patients with an increased baseline level of high-sensitivity troponin, an increase of at least 1.5 from baseline value was considered as significant.[16] TMA was characterized by microangiopathic hemolytic anemia, thrombocytopenia, and microthrombi leading to ischemic tissue injury.[17] AEs were assessed for clinical significance based on the need for hospitalization or end-organ functional damage. Resolution was determined if the complication was resolved at the end of the study period.

Real-world database search

We performed an observational cross-sectional study focusing on the reporting of myocarditis, hepatitis, and TMA using two international pharmacovigilance databases, VigiBase[18] and FAERS.[19] Commercially available gene replacement therapy drugs were searched and two independent investigators (N.M. and E.S.) adjudicated the potential occurrence of study-defined myocarditis, hepatotoxicity, and TMA for each drug based on the information provided in the database reports (e.g., MedDRA terms, narrative summaries if available). The adjudication aimed to align pharmacovigilance reports with the clinical definitions used for the analysis. Disagreements in adjudication were resolved as detailed above.

Statistical analysis

A random-effects meta-analysis models using restricted maximum likelihood estimation was fitted and the method of Hartung, Knapp, Sidik, and Jonkman adjustments was used to synthesize estimates and confidence intervals.[20,21] Heterogeneity was quantified using the I^2 statistic and p values were calculated from a χ^2 test.[22] Assessment of small-study effects, encompassing publication bias, outcome reporting bias, and clinical heterogeneity, was conducted statistically using the Egger test. Pooled estimates and their confidence bounds were back-transformed to the original proportion or rate scales via the inverse logit or exponential functions, and represented as percentages or events per 100 patient-years. To formally test whether

AEs differed between clinical trials and observational studies, we performed a random-effects meta-regression on the logit-transformed incidence proportions. We also reconstructed an individual participant dataset ("pseudo-IPD") by expanding each study's published cross-tabulations (vector class × dose × immunosuppression strata event counts) into patient-level rows. We then fit one-stage population-average logistic regression models using generalized estimating equations with an exchangeable working correlation for study identifier, modeling the binary outcome of any AE. Predictor variables were AAV-vector immunogenicity (high, intermediate, or low, based on pre-existing vector immunogenicity data from the literature, i.e., the prevalence of pre-existing neutralizing antibodies for a specific serotype), viral load dose category ($\leq 10^{12}$ vs. $>10^{12}$ vg/kg) and immunosuppressive regimen. The latter were defined with *a priori* pragmatic categories as reactive corticosteroids only, pre- and post-corticosteroids, pre- and post-corticosteroids plus adjunctive immunosuppression. This grouping was chosen to preserve statistical power and to detect broad signals of effect, since these different agents act on distinct immunologic pathways. All analyses were performed in the statistical programming environment R version 4.3.1 (R Studio).

ACKNOWLEDGMENTS

I.O. has received grants from Bristol Meier Squibb, Cytokinetics, Amicus, Genzyme, Shire, Bayer, Boston Scientific, Menarini International. E.A. received a grant from the Italian Ministry of Health (GR-2019-12368506; principal investigator of the investigator-driven MYTHS [Myocarditis Therapy with Steroids] trial) and a grant from the Italian Ministry of Health and NextGenerationEU (PNRR-MAD-2022-12376225). E.S. is a research Fellow supported by Sarnoff Cardiovascular Research Foundation. Q.B. has received a grant from the AHA (24CDA1272533). N.M. has received grants from Bristol Meier Squibb, Amicus, Foundation CVCL, AICARM APS Onlus, Bangarter-Rhyner Foundation.

AUTHOR CONTRIBUTIONS

N.M. was responsible for data collection, data analysis, drafting of the manuscript, critical revision. E.A. was involved in project ideation, drafting of the manuscript, and critical revision. E.S. contributed in data collection, data analysis, and drafting of the manuscript. K.H. and Q.B. were implicated in the drafting of the manuscript and critical revision. A.A. and I.O. provided critical revision of the manuscript. E.D.A. was involved in the project ideation, drafting of the manuscript, and critical revision.

DECLARATION OF INTERESTS

I.O. has received fees (honoraria or consulting) from Bristol Meier Squibb, Cytokinetics, Amicus, Genzyme, Shire, and Boston Scientific. E.D.A. is the Chief Scientific Officer: Lexeo Therapeutics and consultant for Kiniksa, serves in the advisory board and shareholder of Rocket Pharmaceuticals, scientific board of ResQue Therapeutics, and scientific Founder of Papillion Therapeutics. E.A. is a consultant for Kiniksa, Cytokinetics, and Lexeo Therapeutics. E.S. is a research Fellow supported by Sarnoff Cardiovascular Research Foundation. Q.B. has received fees (honoraria or consulting) from Papillon Therapeutics. N.M. has received fees (honoraria or consulting) from Bristol Meier Squibb and Academic CME and Atheneum Partners.

SUPPLEMENTAL INFORMATION

Supplemental information can be found online at https://doi.org/10.1016/j.ymthe.2026.01.004.

REFERENCES

1. Wang, J.H., Gessler, D.J., Zhan, W., Gallagher, T.L., and Gao, G. (2024). Adeno-associated virus as a delivery vector for gene therapy of human diseases. Signal Transduct. Target. Ther. *9*, 78.
2. Costa Verdera, H., Kuranda, K., and Mingozzi, F. (2020). AAV Vector Immunogenicity in Humans: A Long Journey to Successful Gene Transfer. Mol. Ther. *28*, 723–746.
3. Argiro, A., Bui, Q., Hong, K.N., Ammirati, E., Olivotto, I., and Adler, E. (2024). Applications of Gene Therapy in Cardiomyopathies. JACC. Heart Fail. *12*, 248–260.
4. Byrne, B.J., Corti, M., and Muntoni, F. (2021). Considerations for Systemic Use of Gene Therapy. Mol. Ther. *29*, 422–423.
5. Silver, E., Argiro, A., Hong, K., and Adler, E. (2024). Gene therapy vector-related myocarditis. Int. J. Cardiol. *398*, 131617.
6. Salabarria, S.M., Corti, M., Coleman, K.E., Wichman, M.B., Berthy, J.A., D'Souza, P., Tifft, C.J., Herzog, R.W., Elder, M.E., Shoemaker, L.R., et al. (2024). Thrombotic microangiopathy following systemic AAV administration is dependent on anti-capsid antibodies. J. Clin. Invest. *134*, e173510.
7. Chand, D., Mohr, F., McMillan, H., Tukov, F.F., Montgomery, K., Kleyn, A., Sun, R., Tauscher-Wisniewski, S., Kaufmann, P., and Kullak-Ublick, G. (2021). Hepatotoxicity following administration of onasemnogene abeparvovec (AVXS-101) for the treatment of spinal muscular atrophy. J. Hepatol. *74*, 560–566.
8. Servais, L., Horton, R., Saade, D., Bonnemann, C., and Muntoni, F.; 261st ENMC workshop study group (2023). 261st ENMC International Workshop: Management of safety issues arising following AAV gene therapy. 17th-19th June 2022, Hoofddorp, The Netherlands. Neuromuscul. Disord. *11*, 884–896.
9. Bonkowsky, J.L., Rajan, D.S., and Eichler, F. (2025). An Imperative for Public Sharing of Adverse Events of Gene Therapy Trials. JAMA Neurol. *82*, 429–430.
10. Stroup, D.F., Berlin, J.A., Morton, S.C., Olkin, I., Williamson, G.D., Rennie, D., Moher, D., Becker, B.J., Sipe, T.A., and Thacker, S.B. (2000). Meta-analysis of observational studies in epidemiology: a proposal for reporting. Meta-analysis Of Observational Studies in Epidemiology (MOOSE) group. JAMA *15*, 2008–2012.
11. Page, M.J., McKenzie, J.E., Bossuyt, P.M., Boutron, I., Hoffmann, T.C., Mulrow, C.D., Shamseer, L., Tetzlaff, J.M., Akl, E.A., Brennan, S.E., et al. (2021). The PRISMA 2020 statement: an updated guideline for reporting systematic reviews. BMJ *372*, N71.
12. https://www.crd.york.ac.uk/PROSPERO/view/CRD420251046546.
13. Sterne, J.A.C., Savović, J., Page, M.J., Elbers, R.G., Blencowe, N.S., Boutron, I., Cates, C.J., Cheng, H.Y., Corbett, M.S., Eldridge, S.M., et al. (2019). RoB 2: a revised tool for assessing risk of bias in randomised trials. BMJ *366*, l4898.
14. Higgins, J.P.T., Altman, D.G., Gøtzsche, P.C., Jüni, P., Moher, D., Oxman, A.D., Savovic, J., Schulz, K.F., Weeks, L., and Sterne, J.A.C.; Cochrane Statistical Methods Group (2011). The Cochrane Collaboration's tool for assessing risk of bias in randomised trials. BMJ *343*, d5928.
15. Ammirati, E., and Moslehi, J.J. (2023). Diagnosis and Treatment of Acute Myocarditis: A Review. JAMA *329*, 1098–1113.
16. Chand, D., Mohr, F., McMillan, H., Tukov, F.F., Montgomery, K., Kleyn, A., Sun, R., Tauscher-Wisniewski, S., Kaufmann, P., and Kullak-Ublick, G. (2021). Hepatotoxicity following administration of onasemnogene abeparvovec (AVXS-101) for the treatment of spinal muscular atrophy. J. Hepatol. *74*, 560–566.
17. Salabarria, S.M., Corti, M., Coleman, K.E., Wichman, M.B., Berthy, J.A., D'Souza, P., Tifft, C.J., Herzog, R.W., Elder, M.E., Shoemaker, L.R., et al. (2024). Thrombotic microangiopathy following systemic AAV administration is dependent on anti-capsid antibodies. J. Clin. Invest. *134*, e173510.
18. IntHout, J., Ioannidis, J.P.A., and Borm, G.F. (2014). The Hartung-Knapp-Sidik-Jonkman method for random effects meta-analysis is straightforward and considerably outperforms the standard DerSimonian-Laird method. BMC Med. Res. Methodol. *14*, 25.
19. Röver, C., Knapp, G., and Friede, T. (2015). Hartung-Knapp-Sidik-Jonkman approach and its modification for random-effects meta-analysis with few studies. BMC Med. Res. Methodol. *15*, 99.
20. Langan, D., Bowden, J., Veroniki, A.A., Kontopantelis, E., Viechtbauer, W., Simmonds, M., Higgins, J.P.T., and Jackson, D. (2019). A comparison of heterogeneity variance estimators in simulated random-effects meta-analyses. Res. Synth. Methods *10*, 83–98.
21. https://www.vigiaccess.org, accessed online on the 24th of April, 2025.

22. https://www.fda.gov/drugs/fdas-adverse-event-reporting-system-faers/fda-adverse-event-reporting-system-faers-public-dashboard, accessed online on the 25th of April, 2025.

23. Greenberg, B., Taylor, M., Adler, E., Colan, S., Ricks, D., Yarabe, P., Battiprolu, P., Shah, G., Patel, K., Coggins, M., et al. (2025). Phase 1 Study of AAV9.LAMP2B Gene Therapy in Danon Disease. N. Engl. J. Med. *392*, 972–983.

24. Hughes, D., Wilcox, W., Hopkin, R.J., Ganesh, J., Bernat, J., Goker-Alpan, O., Nicholls, K., Deegan, P., Pahl, M., Whitley, C.B., et al. (2025). Isaralgagene civaparvovec (ST-920) gene therapy in adults with Fabry disease: Updated results from an ongoing phase 1/2 study (STAAR). Mol. Genet. Metab. *44*, 108773.

25. Mendell, J.R., Sahenk, Z., Lehman, K.J., Lowes, L.P., Reash, N.F., Iammarino, M.A., Alfano, L.N., Lewis, S., Church, K., Shell, R., et al. (2024). Long-term safety and functional outcomes of delandistrogene moxeparvovec gene therapy in patients with Duchenne muscular dystrophy: A phase 1/2a nonrandomized trial. Muscle Nerve *69*, 93–98.

26. Bönnemann, C.G., Belluscio, B.A., Braun, S., Morris, C., Singh, T., and Muntoni, F. (2023). Dystrophin Immunity after Gene Therapy for Duchenne's Muscular Dystrophy. N. Engl. J. Med. *388*, 2294–2296.

27. Mendell, J.R., Sahenk, Z., Lehman, K., Nease, C., Lowes, L.P., Miller, N.F., Iammarino, M.A., Alfano, L.N., Nicholl, A., Al-Zaidy, S., et al. (2020). Assessment of Systemic Delivery of rAAVrh74.MHCK7.micro-dystrophin in Children With Duchenne Muscular Dystrophy: A Nonrandomized Controlled Trial. JAMA Neurol. *77*, 1122–1131.

28. Zaidman, C.M., Proud, C.M., McDonald, C.M., Lehman, K.J., Goedeker, N.L., Mason, S., Murphy, A.P., Guridi, M., Wang, S., Reid, C., et al. (2023). Delandistrogene Moxeparvovec Gene Therapy in Ambulatory Patients (Aged $\geq$4 to <8 Years) with Duchenne Muscular Dystrophy: 1-Year Interim Results from Study SRP-9001-103 (ENDEAVOR). Ann. Neurol. *94*, 955–968.

29. Mendell, J.R., Shieh, P.B., McDonald, C.M., Sahenk, Z., Lehman, K.J., Lowes, L.P., Reash, N.F., Iammarino, M.A., Alfano, L.N., Sabo, B., et al. (2023). Expression of SRP-9001 dystrophin and stabilization of motor function up to 2 years post-treatment with delandistrogene moxeparvovec gene therapy in individuals with Duchenne muscular dystrophy. Front. Cell Dev. Biol. *11*, 1167762.

30. Donisa, D.R., Redican, S., Lawrence, J., Brown, K., Wang, F., Gonzalez, F., Schneider, J., Morris, C., Shieh, P., and Byrne, B. (2022). FP.28 IGNITE DMD phase I/II study of SGT-001 microdystrophin gene therapy for DMD: Long-term outcomes and expression update. Neuromuscul. Disord. *32*, S98.

31. Laugel, V., De Lucia, S., Davion, J., Daniele, N., Cao, F., Sanz, M., Buscara, L., Blaie, S., Thibaut, L., Sagot, M., et al. (2024). 410P GNT0004, Genethon's AAV8 vector-delivered microdystrophin gene therapy of Duchenne muscular dystrophy, first data of the phase I/II part of the GNT-016-MDYF all-in-one clinical trial in ambulant boys. Neuromuscul. Disord. *43*, 104441-281.

32. Lek, A., Wong, B., Keeler, A., Blackwood, M., Ma, K., Huang, S., Sylvia, K., Batista, A.R., Artinian, R., Kokoski, D., et al. (2023). Death after High-Dose rAAV9 Gene Therapy in a Patient with Duchenne's Muscular Dystrophy. N. Engl. J. Med. *389*, 1203–1210.

33. Mendell, J.R., Muntoni, F., McDonald, C.M., Mercuri, E.M., Ciafaloni, E., Komaki, H., Leon-Astudillo, C., Nascimento, A., Proud, C., Schara-Schmidt, U., et al. (2025). AAV gene therapy for Duchenne muscular dystrophy: the EMBARK phase 3 randomized trial. Nat. Med. *31*, 332–341.

34. Flanigan, K.M., Vetter, T.A., Simmons, T.R., Iammarino, M., Frair, E.C., Rinaldi, F., Chicoine, L.G., Harris, J., Cheatham, J.P., Cheatham, S.L., et al. (2022). A first-in-human phase I/IIa gene transfer clinical trial for Duchenne muscular dystrophy using rAAVrh74.MCK.*GALGT2*. Mol. Ther. Methods Clin. Dev. *27*, 47–60.

35. Bowles, D.E., McPhee, S.W.J., Li, C., Gray, S.J., Samulski, J.J., Camp, A.S., Li, J., Wang, B., Monahan, P.E., Rabinowitz, J.E., et al. (2012). Phase 1 gene therapy for Duchenne muscular dystrophy using a translational optimized AAV vector. Mol. Ther. *20*, 443–455.

36. Mendell, J.R., Rodino-Klapac, L.R., Rosales, X.Q., Coley, B.D., Galloway, G., Lewis, S., Malik, V., Shilling, C., Byrne, B.J., Conlon, T., et al. (2010). Sustained alpha-sarcoglycan gene expression after gene transfer in limb-girdle muscular dystrophy, type 2D. Ann. Neurol. *68*, 629–638.

37. Mendell, J.R., Pozsgai, E.R., Lewis, S., Griffin, D.A., Lowes, L.P., Alfano, L.N., Lehman, K.J., Church, K., Reash, N.F., Iammarino, M.A., et al. (2024). Gene therapy with bidridistrogene xeboparvovec for limb-girdle muscular dystrophy type 2E/R4: phase 1/2 trial results. Nat. Med. *30*, 199–206.

38. Smith, E.C., Hopkins, S., Case, L.E., Xu, M., Walters, C., Dearmey, S., Han, S.O., Spears, T.G., Chichester, J.A., Bossen, E.H., et al. (2023). Phase I study of liver depot gene therapy in late-onset Pompe disease. Mol. Ther. *31*, 1994–2004.

39. Smith, B.K., Collins, S.W., Conlon, T.J., Mah, C.S., Lawson, L.A., Martin, A.D., Fuller, D.D., Cleaver, B.D., Clément, N., Phillips, D., et al. (2013). Phase I/II trial of adeno-associated virus-mediated alpha-glucosidase gene therapy to the diaphragm for chronic respiratory failure in Pompe disease: initial safety and ventilatory outcomes. Hum. Gene Ther. *24*, 630–640.

40. Corti, M., Liberati, C., Smith, B.K., Lawson, L.A., Tuna, I.S., Conlon, T.J., Coleman, K.E., Islam, S., Herzog, R.W., Fuller, D.D., et al. (2017). Safety of Intradiaphragmatic Delivery of Adeno-Associated Virus-Mediated Alpha-Glucosidase (rAAV1-CMV-hGAA) Gene Therapy in Children Affected by Pompe Disease. Hum. Gene Ther. Clin. Dev. *28*, 208–218.

41. Strauss, K.A., Farrar, M.A., Muntoni, F., Saito, K., Mendell, J.R., Servais, L., McMillan, H.J., Finkel, R.S., Swoboda, K.J., Kwon, J.M., et al. (2022). Onasemnogene abeparvovec for presymptomatic infants with three copies of SMN2 at risk for spinal muscular atrophy: the Phase III SPR1NT trial. Nat. Med. *28*, 1390–1397.

42. Strauss, K.A., Farrar, M.A., Muntoni, F., Saito, K., Mendell, J.R., Servais, L., McMillan, H.J., Finkel, R.S., Swoboda, K.J., Kwon, J.M., et al. (2022). Onasemnogene abeparvovec for presymptomatic infants with two copies of SMN2 at risk for spinal muscular atrophy type 1: the Phase III SPR1NT trial. Nat. Med. *28*, 1381–1389.

43. Mendell, J.R., Al-Zaidy, S., Shell, R., Arnold, W.D., Rodino-Klapac, L.R., Prior, T.W., Lowes, L., Alfano, L., Berry, K., Church, K., et al. (2017). Single-Dose Gene-Replacement Therapy for Spinal Muscular Atrophy. N. Engl. J. Med. *377*, 1713–1722.

44. Day, J.W., Finkel, R.S., Chiriboga, C.A., Connolly, A.M., Crawford, T.O., Darras, B.T., Iannaccone, S.T., Kuntz, N.L., Peña, L.D.M., Shieh, P.B., et al. (2021). Onasemnogene abeparvovec gene therapy for symptomatic infantile-onset spinal muscular atrophy in patients with two copies of SMN2 (STR1VE): an open-label, single-arm, multicentre, phase 3 trial. Lancet Neurol. *20*, 284–293.

45. Mercuri, E., Muntoni, F., Baranello, G., Masson, R., Boespflug-Tanguy, O., Bruno, C., Corti, S., Daron, A., Deconinck, N., Servais, L., et al. (2021). Onasemnogene abeparvovec gene therapy for symptomatic infantile-onset spinal muscular atrophy type 1 (STR1VE-EU): an open-label, single-arm, multicentre, phase 3 trial. Lancet Neurol. *20*, 832–841.

46. Guillou, J., de Pellegars, A., Porcheret, F., Frémeaux-Bacchi, V., Allain-Launay, E., Debord, C., Denis, M., Péréon, Y., Barnérias, C., Desguerre, I., et al. (2022). Fatal thrombotic microangiopathy case following adeno-associated viral SMN gene therapy. Blood Adv. *6*, 4266–4270.

47. Chand, D.H., Zaidman, C., Arya, K., Millner, R., Farrar, M.A., Mackie, F.E., Goedeker, N.L., Dharnidharka, V.R., Dandamudi, R., and Reyna, S.P. (2021). Thrombotic Microangiopathy Following Onasemnogene Abeparvovec for Spinal Muscular Atrophy: A Case Series. J. Pediatr. *231*, 265–268.

48. Finkel, R.S., Darras, B.T., Mendell, J.R., Day, J.W., Kuntz, N.L., Connolly, A.M., Zaidman, C.M., Crawford, T.O., Butterfield, R.J., Shieh, P.B., et al. (2023). Intrathecal Onasemnogene Abeparvovec for Sitting, Nonambulatory Patients with Spinal Muscular Atrophy: Phase I Ascending-Dose Study (STRONG). J. Neuromuscul. Dis. *10*, 389–404.

49. Gowda, V., Atherton, M., Murugan, A., Servais, L., Sheehan, J., Standing, E., Manzur, A., Scoto, M., Baranello, G., Munot, P., et al. (2024). Efficacy and safety of onasemnogene abeparvovec in children with spinal muscular atrophy type 1: real-world evidence from 6 infusion centres in the United Kingdom. Lancet Reg. Health Eur. *37*, 100817.

50. Servais, L., Day, J.W., De Vivo, D.C., Kirschner, J., Mercuri, E., Muntoni, F., Proud, C.M., Shieh, P.B., Tizzano, E.F., Quijano-Roy, S., et al. (2024). Real-World Outcomes in Patients with Spinal Muscular Atrophy Treated with Onasemnogene Abeparvovec Monotherapy: Findings from the RESTORE Registry. J. Neuromuscul. Dis. *11*, 425–442.

51. Weiß, C., Ziegler, A., Becker, L.L., Johannsen, J., Brennenstuhl, H., Schreiber, G., Flotats-Bastardas, M., Stoltenburg, C., Hartmann, H., Illsinger, S., et al. (2022). Gene replacement therapy with onasemnogene abeparvovec in children with spinal muscular atrophy aged 24 months or younger and bodyweight up to 15 kg: an observational cohort study. Lancet Child Adolesc. Health *6*, 17–27.
52. Bitetti, I., Lanzara, V., Margiotta, G., and Varone, A. (2023). Onasemnogene abeparvovec gene replacement therapy for the treatment of spinal muscular atrophy: a real-world observational study. Gene Ther. *30*, 592–597.
53. Mendell, J.R., Al-Zaidy, S.A., Lehman, K.J., McColly, M., Lowes, L.P., Alfano, L.N., Reash, N.F., Iammarino, M.A., Church, K.R., Kleyn, A., et al. (2021). Five-Year Extension Results of the Phase 1 START Trial of Onasemnogene Abeparvovec in Spinal Muscular Atrophy. JAMA Neurol. *78*, 834–841.
54. Favia, M., Tarantino, D., Cerbo, L.D., Sabia, A., Campopiano, R., and Pani, M. (2024). Onasemnogene Abeparvovec: Post-infusion Efficacy and Safety in Patients With Spinal Muscular Atrophy (SMA)-A Fondazione Policlinico Gemelli IRCCS Experience. Hosp. Pharm. *59*, 39–46.
55. Waldrop, M.A., Chagat, S., Storey, M., Meyer, A., Iammarino, M., Reash, N., Alfano, L., Lowes, L., Noritz, G., Prochoroff, A., et al. (2024). Continued safety and long-term effectiveness of onasemnogene abeparvovec in Ohio. Neuromuscul. Disord. *34*, 41–48.
56. Waldrop, M.A., Karingada, C., Storey, M.A., Powers, B., Iammarino, M.A., Miller, N.F., Alfano, L.N., Noritz, G., Rossman, I., Ginsberg, M., et al. (2020). Gene Therapy for Spinal Muscular Atrophy: Safety and Early Outcomes. Pediatrics *146*, e20200729.
57. Chencheri, N., Alexander, G., Nugud, A., Majadas, E., Salim, H., Prudhomme, K., DeJager, N., Janardhanan, V.S., and Elbashir, H. (2023). Gene transfer therapy in children with spinal muscular atrophy: A single-center experience with a cohort of 25 children. Muscle Nerve *68*, 269–277.
58. Pane, M., Coratti, G., Sansone, V.A., Messina, S., Catteruccia, M., Bruno, C., Sframeli, M., Albamonte, E., Pedemonte, M., Brolatti, N., et al. (2023). Type I spinal muscular atrophy patients treated with nusinersen: 4-year follow-up of motor, respiratory and bulbar function. Eur. J. Neurol. *30*, 1755–1763.
59. Tokatly Latzer, I., Sagi, L., Lavi, R., Aharoni, S., Bistritzer, J., Noyman, I., Ginsburg, M., Lev-Or, A., Katzenellenbogen, S., Nevo, Y., and Fattal-Valevski, A. (2023). Real-Life Outcome After Gene Replacement Therapy for Spinal Muscular Atrophy: A Multicenter Experience. Pediatr. Neurol. *144*, 60–68.Stettner, G.M., Hasselmann, O., Tscherter, A., Galiart, E., Jacquier, D., and Klein, A. (2023). Treatment of spinal muscular atrophy with Onasemnogene Abeparvovec in Switzerland: a prospective observational case series study. BMC Neurol. *23*, 88.
60. Matesanz, S.E., Battista, V., Flickinger, J., Jones, J.N., and Kichula, E.A. (2021). Clinical Experience With Gene Therapy in Older Patients With Spinal Muscular Atrophy. Pediatr. Neurol. *118*, 1–5.
61. Ali, H.G., Ibrahim, K., Elsaid, M.F., Mohamed, R.B., Abeidah, M.I.A., Al Rawwas, A.O., Elshafey, K., Almulla, H., El-Akouri, K., Almulla, M., et al. (2021). Gene therapy for spinal muscular atrophy: the Qatari experience. Gene Ther. *28*, 676–680.
62. D'Silva, A.M., Holland, S., Kariyawasam, D., Herbert, K., Barclay, P., Cairns, A., MacLennan, S.C., Ryan, M.M., Sampaio, H., Smith, N., et al. (2022). Onasemnogene abeparvovec in spinal muscular atrophy: an Australian experience of safety and efficacy. Ann. Clin. Transl. Neurol. *9*, 339–350.
63. Friese, J., Geitmann, S., Holzwarth, D., Müller, N., Sassen, R., Baur, U., Adler, K., and Kirschner, J. (2021). Safety Monitoring of Gene Therapy for Spinal Muscular Atrophy with Onasemnogene Abeparvovec -A Single Centre Experience. J. Neuromuscul. Dis. *8*, 209–216.
64. Shieh, P.B., Kuntz, N.L., Dowling, J.J., Müller-Felber, W., Bönnemann, C.G., Seferian, A.M., Servais, L., Smith, B.K., Muntoni, F., Blaschek, A., et al. (2023). Safety and efficacy of gene replacement therapy for X-linked myotubular myopathy (ASPIRO): a multinational, open-label, dose-escalation trial. Lancet Neurol. *22*, 1125–1139.
65. Ozelo, M.C., Mahlangu, J., Pasi, K.J., Giermasz, A., Leavitt, A.D., Laffan, M., Symington, E., Quon, D.V., Wang, J.D., Peerlinck, K., et al. (2022). Valoctocogene Roxaparvovec Gene Therapy for Hemophilia A. N. Engl. J. Med. *386*, 1013–1025.
66. Ozelo, M.C., Mason, J., Dunn, A.L., Villaça, P.R., Shen, M.C., Agarwal, S., Imtiaz, U., Liu, H., and Robinson, T.M. (2025). Safety and efficacy of valoctocogene roxaparvovec with prophylactic glucocorticoids: 1-year results from the phase 3b, single-arm, open-label GENEr8-3 study. J. Thromb. Haemost. *23*, 1496–1506.
67. Rangarajan, S., Walsh, L., Lester, W., Perry, D., Madan, B., Laffan, M., Yu, H., Vettermann, C., Pierce, G.F., Wong, W.Y., and Pasi, K.J. (2017). AAV5-Factor VIII Gene Transfer in Severe Hemophilia A. N. Engl. J. Med. *377*, 2519–2530.
68. Mahlangu, J., Kaczmarek, R., von Drygalski, A., Shapiro, S., Chou, S.C., Ozelo, M.C., Kenet, G., Peyvandi, F., Wang, M., Madan, B., et al. (2023). Two-Year Outcomes of Valoctocogene Roxaparvovec Therapy for Hemophilia A. N. Engl. J. Med. *388*, 694–705.
69. Leavitt, A.D., Konkle, B.A., Stine, K.C., Visweshwar, N., Harrington, T.J., Giermasz, A., Arkin, S., Fang, A., Plonski, F., Yver, A., et al. (2024). Giroctocogene fitelparvovec gene therapy for severe hemophilia A: 104-week analysis of the phase 1/2 Alta study. Blood *143*, 796–806.
70. Chapin, J., Álvarez Román, M.T., Ayash-Rashkovsky, M., Diogo, D., Kenniston, J., Lopez-Jaime, F.J., Maggiore, C., Mingot-Castellano, M.E., Rajavel, K., Rauch, A., et al. (2025). A phase 1/2 safety and efficacy study of TAK-754 gene therapy: The challenge of achieving durable factor VIII expression in haemophilia A clinical trials. Haemophilia. *31*, 108–117.
71. George, L.A., Monahan, P.E., Eyster, M.E., Sullivan, S.K., Ragni, M.V., Croteau, S.E., Rasko, J.E.J., Recht, M., Samelson-Jones, B.J., MacDougall, A., et al. (2021). Multiyear Factor VIII Expression after AAV Gene Transfer for Hemophilia A. N. Engl. J. Med. *385*, 1961–1973.
72. Manno, C.S., Pierce, G.F., Arruda, V.R., Glader, B., Ragni, M., Rasko, J.J., Ozelo, M.C., Hoots, K., Blatt, P., Konkle, B., et al. (2006). Successful transduction of liver in hemophilia by AAV-Factor IX and limitations imposed by the host immune response. Nat. Med. *12*, 342–347.
73. Pipe, S.W., Leebeek, F.W.G., Recht, M., Key, N.S., Castaman, G., Miesbach, W., Lattimore, S., Peerlinck, K., Van der Valk, P., Coppens, M., et al. (2023). Gene Therapy with Etranacogene Dezaparvovec for Hemophilia B. N. Engl. J. Med. *388*, 706–718.
74. George, L.A., Sullivan, S.K., Giermasz, A., Rasko, J.E.J., Samelson-Jones, B.J., Ducore, J., Cuker, A., Sullivan, L.M., Majumdar, S., Teitel, J., et al. (2017). Hemophilia B Gene Therapy with a High-Specific-Activity Factor IX Variant. N. Engl. J. Med. *377*, 2215–2227.
75. Nathwani, A.C., Tuddenham, E.G.D., Rangarajan, S., Rosales, C., McIntosh, J., Linch, D.C., Chowdary, P., Riddell, A., Pie, A.J., Harrington, C., et al. (2011). Adenovirus-associated virus vector-mediated gene transfer in hemophilia B. N. Engl. J. Med. *365*, 2357–2365.
76. Chowdary, P., Shapiro, S., Makris, M., Evans, G., Boyce, S., Talks, K., Dolan, G., Reiss, U., Phillips, M., Riddell, A., et al. (2022). Phase 1-2 Trial of AAVS3 Gene Therapy in Patients with Hemophilia B. N. Engl. J. Med. *387*, 237–247.
77. Cuker, A., Kavakli, K., Frenzel, L., Wang, J.D., Astermark, J., Cerqueira, M.H., Iorio, A., Katsarou-Fasouli, O., Klamroth, R., Shapiro, A.D., et al. (2024). Gene Therapy with Fidanacogene Elaparvovec in Adults with Hemophilia B. N. Engl. J. Med. *391*, 1108–1118.
78. Coppens, M., Pipe, S.W., Miesbach, W., Astermark, J., Recht, M., van der Valk, P., Ewenstein, B., Pinachyan, K., Galante, N., Le Quellec, S., et al. (2024). Etranacogene dezaparvovec gene therapy for haemophilia B (HOPE-B): 24-month post-hoc efficacy and safety data from a single-arm, multicentre, phase 3 trial. Lancet Haematol. *11*, e265–e275.
79. Tai, C.H., Lee, N.C., Chien, Y.H., Byrne, B.J., Muramatsu, S.I., Tseng, S.H., and Hwu, W.L. (2022). Long-term efficacy and safety of eladocagene exuparvovec in patients with AADC deficiency. Mol. Ther. *30*, 509–518.
80. Lv, J., Wang, H., Cheng, X., Chen, Y., Wang, D., Zhang, L., Cao, Q., Tang, H., Hu, S., Gao, K., et al. (2024). AAV1-hOTOF gene therapy for autosomal recessive deafness 9: a single-arm trial. Lancet *403*, 2317–2325.
81. Sevigny, J., Uspenskaya, O., Heckman, L.D., Wong, L.C., Hatch, D.A., Tewari, A., Vandenberghe, R., Irwin, D.J., Saracino, D., Le Ber, I., et al. (2024). Progranulin AAV gene therapy for frontotemporal dementia: translational studies and phase 1/2 trial interim results. Nat. Med. *30*, 1406–1415.

82. D'Antiga, L., Beuers, U., Ronzitti, G., Brunetti-Pierri, N., Baumann, U., Di Giorgio, A., Aronson, S., Hubert, A., Romano, R., Junge, N., et al. (2023). Gene Therapy in Patients with the Crigler-Najjar Syndrome. N. Engl. J. Med. *389*, 620–631.

83. Brunetti-Pierri, N., Ferla, R., Ginocchio, V.M., Rossi, A., Fecarotta, S., Romano, R., Parenti, G., Yildiz, Y., Zancan, S., Pecorella, V., et al. (2022). Liver-Directed Adeno-Associated Virus-Mediated Gene Therapy for Mucopolysaccharidosis Type VI. NEJM Evid. *1*, EVIDoa2200052.

84. Tardieu, M., Zérah, M., Husson, B., de Bournonville, S., Deiva, K., Adamsbaum, C., Vincent, F., Hocquemiller, M., Broissand, C., Furlan, V., et al. (2014). Intracerebral administration of adeno-associated viral vector serotype rh.10 carrying human SGSH and SUMF1 cDNAs in children with mucopolysaccharidosis type IIIA disease: results of a phase I/II trial. Hum. Gene Ther. *25*, 506–516.

85. Tardieu, M., Zérah, M., Gougeon, M.L., Ausseil, J., de Bournonville, S., Husson, B., Zafeiriou, D., Parenti, G., Bourget, P., Poirier, B., et al. (2017). Intracerebral gene therapy in children with mucopolysaccharidosis type IIIB syndrome: an uncontrolled phase 1/2 clinical trial. Lancet Neurol. *16*, 712–720.

86. Deiva, K., Ausseil, J., de Bournonville, S., Zérah, M., Husson, B., Gougeon, M.L., Poirier-Beaudouin, B., Zafeiriou, D., Parenti, G., Heard, J.M., and Tardieu, M. (2021). Intracerebral Gene Therapy in Four Children with Sanfilippo B Syndrome: 5.5-Year Follow-Up Results. Hum. Gene Ther. *32*, 1251–1259.

87. Mendell, J.R., Sahenk, Z., Malik, V., Gomez, A.M., Flanigan, K.M., Lowes, L.P., Alfano, L.N., Berry, K., Meadows, E., Lewis, S., et al. (2015). A phase 1/2a follistatin gene therapy trial for becker muscular dystrophy. Mol. Ther. *23*, 192–201.

88. Lyon, A.R., Babalis, D., Morley-Smith, A.C., Hedger, M., Suarez Barrientos, A., Foldes, G., Couch, L.S., Chowdhury, R.A., Tzortzis, K.N., Peters, N.S., et al. (2020). Investigation of the safety and feasibility of AAV1/SERCA2a gene transfer in patients with chronic heart failure supported with a left ventricular assist device - the SERCA-LVAD TRIAL. Gene Ther. *27*, 579–590.

89. Hammond, H.K., Penny, W.F., Traverse, J.H., Henry, T.D., Watkins, M.W., Yancy, C.W., Sweis, R.N., Adler, E.D., Patel, A.N., Murray, D.R., et al. (2016). Intracoronary Gene Transfer of Adenylyl Cyclase 6 in Patients With Heart Failure: A Randomized Clinical Trial. JAMA Cardiol. *1*, 163–171.

90. Jaski, B.E., Jessup, M.L., Mancini, D.M., Cappola, T.P., Pauly, D.F., Greenberg, B., Borow, K., Dittrich, H., Zsebo, K.M., and Hajjar, R.J.; Calcium Up-Regulation by Percutaneous Administration of Gene Therapy In Cardiac Disease CUPID Trial Investigators (2009). Calcium upregulation by percutaneous administration of gene therapy in cardiac disease (CUPID Trial), a first-in-human phase 1/2 clinical trial. J. Card. Fail. *15*, 171–181.

91. Jessup, M., Greenberg, B., Mancini, D., Cappola, T., Pauly, D.F., Jaski, B., Yaroshinsky, A., Zsebo, K.M., Dittrich, H., and Hajjar, R.J.; Calcium Upregulation by Percutaneous Administration of Gene Therapy in Cardiac Disease CUPID Investigators (2011). Calcium Upregulation by Percutaneous Administration of Gene Therapy in Cardiac Disease (CUPID): a phase 2 trial of intracoronary gene therapy of sarcoplasmic reticulum Ca2+-ATPase in patients with advanced heart failure. Circulation *124*, 304–313.

92. Greenberg, B., Butler, J., Felker, G.M., Ponikowski, P., Voors, A.A., Desai, A.S., Barnard, D., Bouchard, A., Jaski, B., Lyon, A.R., et al. (2016). Calcium upregulation by percutaneous administration of gene therapy in patients with cardiac disease (CUPID 2): a randomised, multinational, double-blind, placebo-controlled, phase 2b trial. Lancet *387*, 1178–1186.

93. Grines, C.L., Watkins, M.W., Helmer, G., Penny, W., Brinker, J., Marmur, J.D., West, A., Rade, J.J., Marrott, P., Hammond, H.K., and Engler, R.L. (2002). Angiogenic Gene Therapy (AGENT) trial in patients with stable angina pectoris. Circulation *105*, 1291–1297.

94. Grines, C.L., Watkins, M.W., Mahmarian, J.J., Iskandrian, A.E., Rade, J.J., Marrott, P., Pratt, C., and Kleiman, N.; Angiogene GENe Therapy AGENT-2 Study Group (2003). A randomized, double-blind, placebo-controlled trial of Ad5FGF-4 gene therapy and its effect on myocardial perfusion in patients with stable angina. J. Am. Coll. Cardiol. *42*, 1339–1347.

95. Nakamura, K., Henry, T.D., Traverse, J.H., Latter, D.A., Mokadam, N.A., Answini, G.A., Williams, A.R., Sun, B.C., Burke, C.R., Bakaeen, F.G., et al. (2024). Angiogenic Gene Therapy for Refractory Angina: Results of the EXACT Phase 2 Trial. Circ. Cardiovasc. Interv. *17*, e014054.

96. D'Avola, D., López-Franco, E., Sangro, B., Pañeda, A., Grossios, N., Gil-Farina, I., Benito, A., Twisk, J., Paz, M., Ruiz, J., et al. (2016). Phase I open label liver-directed gene therapy clinical trial for acute intermittent porphyria. J. Hepatol. *65*, 776–783.

97. Stewart, D.J., Hilton, J.D., Arnold, J.M.O., Gregoire, J., Rivard, A., Archer, S.L., Charbonneau, F., Cohen, E., Curtis, M., Buller, C.E., et al. (2006). Angiogenic gene therapy in patients with nonrevascularizable ischemic heart disease: a phase 2 randomized, controlled trial of AdVEGF(121) (AdVEGF121) versus maximum medical treatment. Gene Ther. *13*, 1503–1511.

98. Ferreira, V., Twisk, J., Kwikkers, K., Aronica, E., Brisson, D., Methot, J., Petry, H., and Gaudet, D. (2014). Immune responses to intramuscular administration of alipogene tiparvovec (AAV1-LPL(S447X)) in a phase II clinical trial of lipoprotein lipase deficiency gene therapy. Hum. Gene Ther. *25*, 180–188.

99. Weinstein, D.A., Derks, T.G., Rodriguez-Buritica, D.F., Ahmad, A., Couce, M.L., Mitchell, J.J., Riba-Wolman, R., Mount, M., Sallago, J.B., Ross, K.M., et al. (2025). Safety and Efficacy of DTX401, an AAV8-Mediated Liver-Directed Gene Therapy, in Adults With Glycogen Storage Disease Type I a (GSDIa). J. Inherit. Metab. Dis. *48*, e70014.

100. Flotte, T.R., Cataltepe, O., Puri, A., Batista, A.R., Moser, R., McKenna-Yasek, D., Douthwright, C., Gernoux, G., Blackwood, M., Mueller, C., et al. (2022). AAV gene therapy for Tay-Sachs disease. Nat. Med. *28*, 251–259.

101. Priddy, F.H., Lewis, D.J.M., Gelderblom, H.C., Hassanin, H., Streatfield, C., LaBranche, C., Hare, J., Cox, J.H., Dally, L., Bendel, D., et al. (2019). Adeno-associated virus vectored immunoprophylaxis to prevent HIV in healthy adults: a phase 1 randomised controlled trial. Lancet HIV *6*, e230–e239.

102. Flotte, T.R., Trapnell, B.C., Humphries, M., Carey, B., Calcedo, R., Rouhani, F., Campbell-Thompson, M., Yachnis, A.T., Sandhaus, R.A., McElvaney, N.G., et al. (2011). Phase 2 clinical trial of a recombinant adeno-associated viral vector expressing α1-antitrypsin: interim results. Hum. Gene Ther. *22*, 1239–1247.

103. Schulz, M., Levy, D.I., Petropoulos, C.J., Bashirians, G., Winburn, I., Mahn, M., Somanathan, S., Cheng, S.H., and Byrne, B.J. (2023). Binding and neutralizing anti-AAV antibodies: Detection and implications for rAAV-mediated gene therapy. Mol. Ther. *31*, 616–630.

104. https://www.mdaconference.org/abstract-library/safety-and-efficacy-of-pf-06939926-gene-therapy-in-boys-with-duchenne-muscular-dystrophy-update-on-data-from-the-phase-1b-study/.

105. https://4dmt.gcs-web.com/news-releases/news-release-details/4dmt-presents-interim-data-4d-310-inglaxa-phase-12-clinical/.

106. Lek, A., Atas, E., Hesterlee, S.E., Byrne, B.J., and Bönnemann, C.G. (2023). Meeting Report: 2022 Muscular Dystrophy Association Summit on 'Safety and Challenges in Gene Transfer Therapy'. J. Neuromuscul. Dis. *10*, 327–336.

107. https://ir.rocketpharma.com/news-releases/news-release-details/rocket-pharmaceuticals-provides-update-phase-2-clinical-trial-rp/.

108. https://investorrelations.sarepta.com/news-releases/news-release-details/sarepta-provides-safety-update-elevidys-and-initiates-steps.

109. Cao, D., Byrne, B.J., de Jong, Y.P., Terhorst, C., Duan, D., Herzog, R.W., and Kumar, S.R.P. (2024). Innate Immune Sensing of Adeno-Associated Virus Vectors. Hum. Gene Ther. *35*, 451–463.

YMTHE, Volume *34*

Supplemental Information

Incidence, timing, and clinical significance of adverse immune events after gene replacement therapy: A systematic review and meta-analysis

Niccolò Maurizi, Enrico Ammirati, Elizabeth Silver, Kimberly Hong, Quan Bui, Alessia Argirò, Iacopo Olivotto, and Eric D. Adler

Table S1. MOOSE (Meta-analyses Of Observational Studies in Epidemiology) Checklist.

Reporting Criteria	Reported (Yes/No)	Reported on Page No.
**Reporting of Background**		
Problem definition	Yes	1
Hypothesis statement	Yes	1
Description of Study Outcome(s)	Yes	5
Type of exposure or intervention used	Yes	4,5
Type of study design used	Yes	4,5
Study population	Yes	4,5
**Reporting of Search Strategy**		
Qualifications of searchers (eg, librarians and investigators)	Yes	4,5
Search strategy, including time period included in the synthesis and keywords	Yes	4
Effort to include all available studies, including contact with authors	Yes	4,5
Databases and registries searched	Yes	4
Search software used, name and version, including special features used (eg, explosion)	Yes	4
Use of hand searching (eg, reference lists of obtained articles)	Yes	6
List of citations located and those excluded, including justification	Yes	7
Method for addressing articles published in languages other than English	No	NA
Method of handling abstracts and unpublished studies	Yes	4
Description of any contact with authors	Yes	4
**Reporting of Methods**		
Description of relevance or appropriateness of studies assembled for assessing the hypothesis to be tested	Yes	5
Rationale for the selection and coding of data (eg, sound clinical principles or convenience)	Yes	5
Documentation of how data were classified and coded (eg, multiple raters, blinding, and interrater reliability)	Yes	5
Assessment of confounding (eg, comparability of cases and controls in studies where appropriate	Yes	5

Assessment of study quality, including blinding of quality assessors; stratification or regression on possible predictors of study results	Yes	5
Assessment of heterogeneity	Yes	5
Description of statistical methods (eg, complete description of fixed or random effects models, justification of whether the chosen models account for predictors of study results, dose-response models, or cumulative meta-analysis) in sufficient detail to be replicated	Yes	6
Provision of appropriate tables and graphics	Yes	6
**Reporting of Results**		
Table giving descriptive information for each study included	Yes	7
Results of sensitivity testing (eg, subgroup analysis)	Yes	7
Indication of statistical uncertainty of findings	Yes	7
**Reporting of Discussion**		
Quantitative assessment of bias (eg, publication bias)	Yes	7
Justification for exclusion (eg, exclusion of non–English-language citations)	No	NA
Assessment of quality of included studies	Yes	7
**Reporting of Conclusions**		
Consideration of alternative explanations for observed results	Yes	10
Generalization of the conclusions (ie, appropriate for the data presented and within the domain of the literature review)	Yes	12
Guidelines for future research	Yes	12
Disclosure of funding source	Yes	1

Table S2. Preferred Reporting Items for Systematic reviews and Meta-Analyses (PRISMA) checklist.

Section and Topic	Item #	Checklist item	Location where item is reported
**TITLE**			
Title	1	Identify the report as a systematic review.	P1
**ABSTRACT**			
Abstract	2	See the PRISMA 2020 for Abstracts checklist.	P2
**INTRODUCTION**			
Rationale	3	Describe the rationale for the review in the context of existing knowledge.	P4
Objectives	4	Provide an explicit statement of the objective(s) or question(s) the review addresses.	P4
**METHODS**			
Eligibility criteria	5	Specify the inclusion and exclusion criteria for the review and how studies were grouped for the syntheses.	P5-P6
Information sources	6	Specify all databases, registers, websites, organisations, reference lists and other sources searched or consulted to identify studies. Specify the date when each source was last searched or consulted.	P4-P5-P6
Search strategy	7	Present the full search strategies for all databases, registers and websites, including any filters and limits used.	P4-P5-P6
Selection process	8	Specify the methods used to decide whether a study met the inclusion criteria of the review, including how many reviewers screened each record and each report retrieved, whether they worked independently, and if applicable, details of automation tools used in the process.	P4-P5
Data collection process	9	Specify the methods used to collect data from reports, including how many reviewers collected data from each report, whether they worked independently, any processes for obtaining or confirming data from study investigators, and if applicable, details of automation tools used in the process.	P5
Data items	10a	List and define all outcomes for which data were sought. Specify whether all results that were compatible with each outcome domain in each study were sought (e.g. for all measures, time points, analyses), and if not, the methods used to decide which results to collect.	P5
	10b	List and define all other variables for which data were sought (e.g. participant and intervention characteristics, funding sources). Describe any assumptions made about any missing or unclear information.	P5
Study risk of bias assessment	11	Specify the methods used to assess risk of bias in the included studies, including details of the tool(s) used, how many reviewers assessed each study and whether they worked independently, and if applicable, details of automation tools used in the process.	P5
Effect measures	12	Specify for each outcome the effect measure(s) (e.g. risk ratio, mean difference) used in the synthesis or presentation of results.	P7
Synthesis methods	13a	Describe the processes used to decide which studies were eligible for each synthesis (e.g. tabulating the study intervention characteristics and comparing against the planned groups for each synthesis (item #5)).	P6-P7
	13b	Describe any methods required to prepare the data for presentation or synthesis, such as handling of missing summary statistics, or data conversions.	P5
	13c	Describe any methods used to tabulate or visually display results of individual studies and syntheses.	P5
	13d	Describe any methods used to synthesize results and provide a rationale for the choice(s). If meta-analysis was performed, describe the model(s), method(s) to identify the presence and extent of statistical heterogeneity, and software package(s) used.	P6
	13e	Describe any methods used to explore possible causes of heterogeneity among study results (e.g. subgroup analysis, meta-regression).	P6
	13f	Describe any sensitivity analyses conducted to assess robustness of the synthesized results.	P6
Reporting bias assessment	14	Describe any methods used to assess risk of bias due to missing results in a synthesis (arising from reporting biases).	P6
Certainty assessment	15	Describe any methods used to assess certainty (or confidence) in the body of evidence for an outcome.	P6

**RESULTS**			
Study selection	16a	Describe the results of the search and selection process, from the number of records identified in the search to the number of studies included in the review, ideally using a flow diagram.	P7
	16b	Cite studies that might appear to meet the inclusion criteria, but which were excluded, and explain why they were excluded.	P7
Study characteristics	17	Cite each included study and present its characteristics.	P7
Risk of bias in studies	18	Present assessments of risk of bias for each included study.	P7
Results of individual studies	19	For all outcomes, present, for each study: (a) summary statistics for each group (where appropriate) and (b) an effect estimate and its precision (e.g. confidence/credible interval), ideally using structured tables or plots.	P7
Results of syntheses	20a	For each synthesis, briefly summarise the characteristics and risk of bias among contributing studies.	P7
	20b	Present results of all statistical syntheses conducted. If meta-analysis was done, present for each the summary estimate and its precision (e.g. confidence/credible interval) and measures of statistical heterogeneity. If comparing groups, describe the direction of the effect.	P7-P8
	20c	Present results of all investigations of possible causes of heterogeneity among study results.	P7-P8
	20d	Present results of all sensitivity analyses conducted to assess the robustness of the synthesized results.	P7
Reporting biases	21	Present assessments of risk of bias due to missing results (arising from reporting biases) for each synthesis assessed.	P7
Certainty of evidence	22	Present assessments of certainty (or confidence) in the body of evidence for each outcome assessed.	P7
**DISCUSSION**			
Discussion	23a	Provide a general interpretation of the results in the context of other evidence.	P9-P10
	23b	Discuss any limitations of the evidence included in the review.	P12-P13
	23c	Discuss any limitations of the review processes used.	P12-P13
	23d	Discuss implications of the results for practice, policy, and future research.	P13
**OTHER INFORMATION**			
Registration and protocol	24a	Provide registration information for the review, including register name and registration number, or state that the review was not registered.	P4
	24b	Indicate where the review protocol can be accessed, or state that a protocol was not prepared.	P4
	24c	Describe and explain any amendments to information provided at registration or in the protocol.	NA
Support	25	Describe sources of financial or non-financial support for the review, and the role of the funders or sponsors in the review.	P1
Competing interests	26	Declare any competing interests of review authors.	P1
Availability of data, code and other materials	27	Report which of the following are publicly available and where they can be found: template data collection forms; data extracted from included studies; data used for all analyses; analytic code; any other materials used in the review.	P4

Table S3. Search criteria for each database and the query used for the search.

Database	Query	Results
Embase<br><br>Embase<br><br>Pubmed	(‘gene replacement therapy’/exp OR ‘gene replacement') AND (’gene therapy trial'/exp OR ‘gene therapy study' OR ‘AAV replacement therapy' OR ‘AAV replacement trial' OR ‘gene therapy trial'/exp OR ‘gene therapy adverse effect' OR ‘gene replacement adverse effect'/exp OR ‘AAV replacement adverse effects' OR ‘real world gene therapy adverse effects' OR ‘real world gene replacement therapy adverse effects'	802

Table S4 Overview of studies included in the systematic-review and meta-analysis.

Abbreviations: AAV: Adenovirus; DMD: Duchenne Muscolar Dystrophy; MD: Muscolar Dystrophy; SMA: Spinal Muscle Atrophy. Follow-up is presented in months and age in years or in months, when specifically indicated.

Study	Year	Diease	Follow-up	Age	Patients	AAV Delivery Vector	Total immune mediated adverse events	Myocarditis	Hepatotoxicity	Thrombotic microangiopathy	Pre-Delivery Immunosuppression	Post-therapy immunosuppression
Greenber et al.	2024[23]	Danon	24	8-20	7	AAV-9	1	0	0	1	Rituximab + Sirolimus	Prednisone, Tacrolimus/Sirolimus, Rituximab
Hughes et al.	2022[24]	Fabry	13	22-48	6	AAV-2	0	0	0	0	-	-
Mendell et al.	2024[25]	DMD	12	-	4	AAVrh74	0	0	0	3	Corticosteroids	Corticosteroids
Bonenemann et al.	2023[26]	DMD	0,3	2	1	AAV-8	1	1	1	0	Corticosteroids	Corticosteroids
Mendell et al.	2020[27]	DMD	12	4-6	4	AAVrh74	1	0	1	0	Prednisone	Prednisone
Zaidman et al.	2023[28]	DMD	12	4-8	20	AAVrh74	2	1	1	0	Corticosteroids	Corticosteroids
Mendell et al.	2023[29]	DMD	12	4-12	20	AAVrh74	0	0	0	0	Corticosteroids	Corticosteroids
Dreghici et al.	2022[30]	DMD	36	-	9	AAV-9	4	0	4	0	-	-
Laugel et al.	2024[31]	DMD	4	6-10	3	AAV2/8	1	0	1	0	Sirolimus	Sirolimus. Corticosteroids
Lek et al.	2023[32]	DMD	0.33	27	1	AAV-9	1	0	1	0	Rituximab, Corticosteroids	Sirolimus, Corticosteroids, Eculizumab, Rituximab, Toculizumab, IVIG
Mendell et al.	2025[33]	DMD	4	4-8	63	AAVrh74	6	1	5	0	Corticosteroids	Corticosteroids
Flaning et al.	2022[34]	DMD	12		2	rAAVrh74	0	0	0	0	Corticosteroids	Corticosteroids
Bowles et al.	2012[35]	DMD	4	15-24	6	AAV 2/5	0	0	0	0	Corticosteroids	Corticosteroids
Mendell et al.	2010[36]	Limb-Girdle MD 2B	6	11-43	6	rAVV-1	0	0	0	0	Corticosteroids	Corticosteroids
Mendell et al.	2024[37]	Limb-Girdle MD 2B	13	4-7	6	rAAV-rh74	1	0	1	0	Corticosteroids	Corticosteroids
Smith et al.	2023[38]	Pompe	13	52-71	3	AAV-8	0	0	0	0	No	Corticosteroids
Smith et al.	2013[39]	Pompe	6	-	5	rAAV (?)	0	0	0	0	No	Corticosteroids
Corti et al.	2018[40]	Pompe	6	2-18	9	AAV-1	0	0	0	0	Rituximab, Sirolimus	Corticosteroids
Strauss et al.	2022[41]	SMA	14	6 m	15	scAAV9	5	2	3	0	Corticosteroids	Corticosteroids
Strauss et al.	2022[42]	SMA	14	6 m	14	scAAV9	4	1	3	0	Corticosteroids	Corticosteroids
Mendell et al.	2017[43]	SMA	24	6 m	15	scAAV9	2	0	2	0	Corticosteroids	Corticosteroids
Day et al.	2021[44]	SMA	12	6 m	20	scAAV9	2	0	2	0	Corticosteroids	Corticosteroids
Mercuri et al.	2021[45]	SMA	12	6 m	33	scAAV9	9	0	9	0	Corticosteroids	Corticosteroids
Goilou et al.	2022[46]	SMA	1	4 m	1	scAAV9	1	0	0	1	Corticosteroids	Corticosteroids
Chand et al.	2021[47]	SMA	16	4-6 m	3	scAAV9	3	0	0	3	Corticosteroids	Corticosteroids
Finekel et al.	2023[48]	SMA	6	4-10 m	32	scAAV9	2	0	0	2	Corticosteroids	Corticosteroids
Gowda et al.	2022[49]	SMA	6	4-14 m	99	scAAV9	99	29	70	0	Corticosteroids	Corticosteroids
Servais et al.	2024[50]	SMA	5	4-14 m	168	scAAV9	67	18	49	1	Corticosteroids	Corticosteroids
Weib et al.	2022[51]	SMA	6	4-14 m	76	scAAV9	58	2	56	0	Corticosteroids	Corticosteroids
Bitetti et al.	2023[52]	SMA	3	4-14 m	9	scAAV9	6	1	5	0	Corticosteroids	Corticosteroids
Mendell et al.	2021[53]	SMA	60	4-14 m	10	scAAV9	0	0	0	0	Corticosteroids	Corticosteroids
Favia et al.	2024[54]	SMA		4-14 m	8	scAAV9	0	0	0	0	Corticosteroids	Corticosteroids
Waldrop et al.	2024[55]	SMA	/	4-14 m	46	scAAV9	18	0	18	0	Corticosteroids	Corticosteroids
Waldrop et al.	2020[56]	SMA	12	4-14 m	21	scAAV9	3	0	3	0	Corticosteroids	Corticosteroids
Chencheri et al.	2023[57]	SMA	6	4-14 m	25	scAAV9	1	0	1	0	Corticosteroids	Corticosteroids
Pane et al.	2023[58]	SMA	/	4-14 m	46	scAAV9	0	0	0	0	Corticosteroids	Corticosteroids
Tokaltly et al.	2023[59]	SMA	12	4-14 m	25	scAAV9	25	10	18	0	Corticosteroids	Corticosteroids
Stettner et al.	2023[60]	SMA	/	4-14 m	9	scAAV9	6	2	4	0	Corticosteroids	Corticosteroids
Matesanz et al.	2021[61]	SMA	/	4-14 m	7	scAAV9	6	0	6	0	Corticosteroids	Corticosteroids
Gaber et al.	2021[62]	SMA	3	4-14 m	9	scAAV9	7	0	7	0	Corticosteroids	Corticosteroids
D'Silva et al.	2022[63]	SMA	12	4-14 m	21	scAAV9	8	0	6	2	Corticosteroids	Corticosteroids
Friese et al.	2021[64]	SMA	6	4-14 m	9	scAAV9	2	0	2	0	Corticosteroids	Corticosteroids
Shieh et al.	2023[65]	X-Lynked Myotub Myo	12	2.5-5	26	AAV-8	9	4	7	0	Corticosteroids	Corticosteroids
Ozelo et al.	2022[66]	Hem A	13	19-43	134	AAV5	108	0	108	0	No	Reactive Corticosteroids
Ozelo et al.	2025[67]	Hem A	12	19-41	22	AAV-5	22	0	22	0	Corticosteroids	Corticosteroids
Manno et al.	200[73]	Hem B	2	-	7	rAVV-2	2	0	2	0	-	-
Pipe et al.	2023[74]	Hem B	13	19-75	54	AAV-5	11	0	11	0	No	Reactive Corticosteroids
Rangarajan et al.	2017[68]	Hem A	13	18-75	9	AAV-5	8	0	8	0	No	Corticosteroids
Mahlangu et al.	2024[69]	Hem A	52	18-47	132	AAV-5	108	0	108	0	No	Reactive Corticosteroids
Leavitt et al.	2024[70]	Hem A	26	19-47	11	rAAV-6	5	0	5	0	No	Corticosteroids
Chapint et al.	2017[71]	Hem A	36	18-45	4	AAV-8	4	0	4	0	No	Reactive Corticosteroids
George et al.	2017[72]	Hem B	12	18-53	10	rAAV-FIX	1	0	1	0	No	Reactive Corticosteroids
George et al.	2021[75]	Hem A	3	18-52	18	AAV-3	7	0	7	0	No	Reactive Corticosteroids
Nathwaani et al.	2011[76]	Hem B	3	31-64	6	scAAV2/8	2	0	2	0	No	Reactive Corticosteroids
Chowdary et al.	2022[77]	Hem B	12	29-67	10	AAV-3	8	0	8	0	No	Corticosteroids, Reactive Tacrolimus

Chowdary et al.	2022[77]	Hem B	12	29-67	10	AAV-3	8	0	8	0	No	Corticosteroids, Reactive Tacrolimus
Coppens et al.	2024[79]	Hem B	24	19-75	54	AAV-2	17	0	17	0	No	Reactive Corticosteroids
Cuker et al.	2024[78]	Hem B	15	18-62	45	AAV-5-FIX	28	0	28	0	No	Corticosteroids
Tai et al.	2022[80]	AADC Def	12	3-16	26	AAV-2	0	0	0	0	No	No
Lv et al.	2024[81]	Aut Rec Deaf	2	1-18	6	AAV-1	0	0	0	0	No	No
Sevignz et al.	2024[82]	Front Deme	12	78-86	19	AAV-9	0	0	0	0	Corticosteroids, Sirolimus, Rituximab	Corticosteroids, Sirolimus, Rituximab
D'Antigo et al.	2023[83]	Crigler Najar	4	18-27	5	AAV-8	4	0	4	0	Corticosteroids, Sirolimus	Corticosteroids, Sirolimus
Tardieu et al.	2014[85]	Mucopolys	12	1-5	4	AAVrh10	0	0	0	0	Corticosteroids	Corticosteroids, Tacrolimus, Mycophenolate
Tardieu et al.	2017[86]	Mucopolys	12	1-5	4	rAAV-2/8	0	0	0	0	Corticosteroids	Corticosteroids, Tacrolimus, Mycophenolate
Deiva et al.	2021[87]	Mucopolys	50	1-5	4	rAAV-2/8	0	0	0	0	Corticosteroids	Corticosteroids, Tacrolimus, Mycophenolate
Brunetti-Pierri et al.	2022[84]	Mucopolys	13	10-29	9	AAV-2/8	2	0	2	0	Corticosteroids	Corticosteroids
Mendell et al.	2015[88]	Becker MD	12	24-37	6	AAV-1	0	0	0	0	Corticosteroids	Corticosteroids
Lyon et al.	2020[89]	CHF	2	29-69	5	AAV-1	0	0	0	0	No	No
Hammond et al.	2016[90]	CHF	12	18-80	42	AAV-5	0	0	0	0	No	No
Stewart et al.	2006[96]	Angina	6	65-78	33	Ad5	0	0	0	0	No	No
Jasky et al.	2009[91]	CHF	12	-	9	AAV-1	0	0	0	0	No	No
Jessup et al.	2011[92]	CHF	12	-	39	AAV-1	0	0	0	0	No	No
Greenber et al.	2016[93]	CHF	12	18-80	123	AAV-1	0	0	0	0	No	No
Grines et al.	2002[94]	Stable Angina	10	35-67	60	AAV-5	2	0	2	0	No	No
Grines et al.	2003[95]	Stable Angina	19	-	52	AAV-5	10	0	10	0	No	No
Nakamura et al.	2024[96]	Stable Angina	12	39-80	32	AAV-5	0	0	0	0	No	No
D'Avola et al.	2016[97]	Porphyria	12	1-13	8	AAV-2/5	0	0	0	0	No	No
Ferreira et al.	2014[98]	Lipase Def	3	-	5	AAV-1	0	0	0	0	Corticosteroids	Corticosteroids
Weinstein et al.	2025[99]	Gly Stor IA	13	19-57	12	AAV-2/8	12	0	12	0	No	Reactive and prophylactic Corticosteroids
Flotte et al.	2022[100]	Tay-Sachs	6	12-14 m	2	AAVrh8	0	0	0	0	Corticosteroids	Corticosteroids
Priddy et al.	2019[101]	HIV	12	18-45	21	AAV-1	0	0	0	0	-	-
Flotte et al.	2011[102]	AAT Deficency	6	-	9	rAAV	0	0	0	0	Corticosteroids	Corticosteroids

Table S5 Critical appraisal of eligible clinical trial and risk of bias using the Cochrane risk-of-bias tool for randomized trials (RoB 2.0).

Domains: D1: Bias arising from the randomization process. D2: Bias due to deviations from intended intervention. D3: Bias due to missing outcome data. D4: Bias in measurement of the outcome.
D5: Bias in selection of the reported result. Judgment: + = Low; - = Some concerns; ? = No information; X = high.

Low Risk
Some concerns
High Risk
No information

Citation	D 1 (randomisation)	D 2 (intended intervention)	D 3 (missing outcome)	D 4 (mesaurment of outcome)	D 5 (selection of results)	Risk of Bias
Greenberg et al. 2025	+	+	+	+	+	+
Hughes et al. 2022	+	+	-	-	-	-
Mendell et al. 2024	+	+	-	-	-	-
Mendell et al. 2020	+	+	+	+	+	+
Zaidman et al. 2023	+	+	+	+	+	+
Mendell et al. 2023	+	+	+	+	+	+
Dreghich et al. 2022	+	+	-	-	-	-
Laugel et al. 2024	+	+	+	+	+	+
Mendell et al. 2025	+	+	+	+	+	+
Flaningn et al. 2022	+	+	+	+	+	+
Bowles et al. 2012	+	+	+	+	+	+
Mendell et al. 2024	+	+	+	+	+	+
Mendell et al. 2010	+	+	+	+	+	+
Smith et al. 2023	+	+	+	+	+	+
Smith et al. 2013	+	+	+	+	+	+
Corti E. et al. 2018	+	+	+	+	+	+
Strauss et al. 2022	+	+	+	+	+	+
Strauss et al. 2022	+	+	+	+	+	+
Mendell et al. 2017	+	+	+	+	+	+
Day et al. 2021	+	+	+	+	+	+
Shieh et al. 2023	+	+	+	+	+	+
Ozelo et al. 2022	+	+	+	+	+	+
Ozelo et al. 2025	+	+	+	+	+	+
Manno et al. 2006	+	X	+	+	+	-
Pipe et al. 2023	+	+	+	+	+	+
Rangarajan et al. 2017	+	+	+	+	+	+
Mahlangu et al. 2024	+	+	+	+	+	+
Leavitt et al. 2024	+	+	+	+	+	+
Chapint. et al. 2017	+	+	+	+	+	+
George L. et al. 2017	+	+	+	+	+	+
George et al. 2021	+	+	+	+	+	+
Mendell et al. 2021	+	+	+	+	+	+

Citation	D 1 (randomisation)	D 2 (intended intervention)	D 3 (missing outcome)	D 4 (mesaurment of outcome)	D 5 (selection of results)	Risk of Bias
Nathwani et al. 2011	-	+	+	+	+	+
Chowdary et al. 2022	+	+	+	+	+	+
Cuker et al. 2024	+	+	+	+	+	+
Coppens et al. 2024	+	+	+	+	+	+
Tai et al. 2022	+	+	+	+	+	+
Lv et al. 2024	+	+	+	+	+	+
Sevignz et al. 2024	+	+	-	-	-	-
D'antigo et al. 2023	+	+	+	+	+	+
Brunetti-Pierri et al. 2022	-	+	+	+	+	-
Tardieu et al. 2014	+	+	+	+	-	-
Tardieu et al. 2017	+	+	+	+	-	-
Deiva et al. 2021	+	+	+	+	+	+
Mendell et al. 2015	+	+	+	+	+	+
Lyon et al. 2020	+	+	+	+	+	+
Hammond et al. 2016	+	+	+	+	+	+
Grines et al. 2002	+	+	+	+	+	+
Grines et al. 2003	+	+	+	+	+	+
Jasky et al. 2009	+	+	+	+	+	+
Jessup et al. 2011	+	+	+	+	+	+
Greenberg et al. 2016	+	+	+	+	+	+
D'Avola et al. 2016	+	+	+	+	+	+
Ferreira et al. 2014	+	?	+	+	+	-
Weinsten et al. 2025	+	+	+	+	+	+
Flotte et al. 2022	+	+	+	+	+	+
Priddy et al. 2019	+	+	+	+	+	+
Flotte et al. 2011	+	+	+	+	+	+
Nagamura et al. 2024	+	+	+	+	+	+

Table S6. Critical appraisal of eligible observational studies and risk of bias using the Cochrane risk-of-bias tool for randomized trials (RoB 1.0).

Domains: D1: Bias arising from cofounding. D2: Bias due to selection. D3: Bias due classification of interventions. D4: Bias due to deviation from intendended intervention.
D5: Bias due to missing data. D6: Bias due to mesaurements of outcome. D7: Bias due to selection of reported outcomes. Judgment: + = Low; - = Some concerns; ? = No information; X = high.

Citation	D 1 (Cofounding)	D 2 (Selection)	D 3 (Classificiaetion of interventions)	D 4 (Deviation from intendend interventions)	D 5 (missing data)	D 6 (measurements of outcome)	D 7 (Selection of reported outcome)	Risk of Bias
Bonnemann et al. 2023	+	-	+	+	+	+	+	-
Lek et al. 2023	-	-	+	+	+	+	+	-
Guilou et al. 2022	+	-	+	+	+	+	+	-
Chand et al. 2021	+	+	+	+	+	+	+	+
Finkel et al. 2023	+	+	+	+	+	+	+	+
Gowda et al. 2024	+	+	+	+	+	+	+	+
Servais et al. 2024	+	+	+	+	+	+	+	+
Weib et al. 2022	+	+	+	+	+	+	+	+
Bitetti et al. 2023	-	-	+	+	+	+	+	-
Favia et al. 2024	-	+	+	+	+	+	+	-
Waldrop et al. 2024	+	+	+	+	+	+	+	+
Waldrop et al. 2020	+	+	+	+	+	+	+	+
Chencheri et al. 2023	+	+	+	+	+	+	+	+
Waldrop et al. 2024	+	+	+	+	+	+	+	+
Waldrop et al. 2020	+	+	+	+	+	+	+	+
Chencheri et al. 2023	+	+	+	+	+	+	+	+
Pane et al. 2023	+	+	+	+	+	+	-	-
Tokatly Latzer et al.	+	+	+	+	+	+	+	+
Stettner et al.	+	+	+	+	+	+	+	+
Matesanz et al.	-	+	+	+	+	+	+	-
Gaber ali et al.	+	+	+	+	+	+	+	+
D'Silva et al	+	+	+	+	+	+	+	+
Friese et al.	+	+	+	+	+	+	+	+

Low Risk | Some concerns | High Risk | No information

Table S7. List of missing data per each study incliuded in the analysis.

Study	Year	Missing Adverse Event Description	Missing Vector Type	Missing Vector Dose	Missing Immunosuppressive Regimen	Missing Follow-up data
Greenber et al.	2024	0	0	0	0	0
Hughes et al.	2022	0	0	0	0	0
Nakamura et al.	2023	0	0	0	0	0
Mendell et al.	2023	0	0	0	0	0
Zaidman et al.	2023	0	0	0	0	0
Bonnemann et al.	2023	0	0	0	0	0
Dreghici et al.	2022	0	0	0	0	0
Laugel et al.	2024	0	0	0	0	0
Lek et al.	2023	0	0	0	0	0
Mendell et al.	2025	0	0	0	0	0
Flaning et al.	2022	0	0	0	0	0
Bowles et al.	2012	0	0	0	0	0
Mendell et al.	2010	0	0	0	0	0
Mendell et al.	2024	0	0	0	0	0
Smith et al.	2023	0	0	0	0	0
Smith et al.	2013	0	0	0	0	0
Corti et al.	2018	0	0	0	0	0
Strauss et al.	2022	0	0	0	0	0
Strauss et al.	2022	0	0	0	0	0
Mendell et al.	2017	0	0	0	0	0
Day et al.	2021	0	0	0	0	0
Merci et al.	2021	0	0	0	0	0
Goilou et al.	2022	0	0	0	0	1
Chand et al.	2021	0	0	0	0	0
Finekel et al.	2023	0	0	0	0	0
Gowda et al.	2022	0	0	0	0	0
Servais et al.	2024	0	0	0	0	0
Weib et al.	2022	0	0	0	0	1
Bitetti et al.	2023	0	0	0	0	0
Mendell et al.	2021	0	0	0	0	0
Favia et al.	2024	0	0	0	0	1
Waldrop et al.	2024	0	0	0	0	0
Waldrop et al.	2020	0	0	0	0	0
Chencheri et al.	2023	0	0	0	0	0
Pane et al.	2023	0	0	0	0	0
Tokaltly et al.	2023	0	0	0	0	1
Stettner et al.	2023	0	0	0	0	0
Matesanz et al.	2021	0	0	0	0	0
Gaber et al.	2021	0	0	0	0	0
D'Silva et al.	2022	0	0	0	0	0
Friese et al.	2021	0	0	0	0	0
Shieh et al.	2023	0	0	0	0	0
Ozelo et al.	2022	0	0	0	0	0
Ozelo et al.	2025	0	0	0	0	0
Manno et al.	2006	0	0	0	0	0
Pipe et al.	2023	0	0	0	0	0
Rangarajan et al.	2017	0	0	0	0	0
Mahlangu et al.	2024	0	0	0	0	0
Leavitt et al.	2024	0	0	0	0	0
Chapint et al.	2017	0	0	0	0	0
George et al.	2017	0	0	0	0	0
George et al.	2021	0	0	0	0	0
Nathwaani et al.	2011	0	0	0	0	0
Chowdary et al.	2022	0	0	0	0	0
Coppens et al.	2024	0	0	0	0	0
Cuker et al.	2024	0	0	0	0	0
Tai et al.	2022	0	0	0	0	0
Lv et al.	2024	0	0	0	0	0
Sevignz et al.	2024	0	0	0	0	0
D'Antigo et al.	2023	0	0	0	0	0
Tardieu et al.	2014	0	0	0	0	0
Tardieu et al.	2017	0	0	0	0	0
Deiva et al.	2021	0	0	0	0	0
Brunetti-Pierri et al.	2022	0	0	0	0	0
Mendell et al.	2015	0	0	0	0	0
Lyon et al.	2020	0	0	0	0	0
Hammond et al.	2016	0	0	0	0	0
Jasky et al.	2009	0	0	0	0	0
Jessup et al.	2011	0	0	0	0	0
Greenber et al.	2016	0	0	0	0	0
Grines et al.	2002	0	0	0	0	0
Grines et al.	2003	0	0	0	0	0
D'Avola et al.	2016	0	0	0	0	0
Ferreira et al.	2014	0	0	0	0	0
Weinstein et al.	2025	0	0	0	0	0
Flotte et al.	2022	0	0	0	0	0
Priddy et al.	2019	0	0	0	1	0
Flotte et al.	2011	0	0	0	0	0

Table S8. Characteristics of the 72 myocarditis episodes related to immune activation after gene replacement therapy.

Abbreviations: AAV: Adenovirus; DMD: Duchenne Muscolar Dystrophy; EF: Ejection Fraction; PVCs: Premature Ventricular Contractions; PLT: Platelets; ARDS: Acute Respiratory Distress Syndrome; ECMO: Extracorporeal Membrane Oxygenation; MOF: Multi Organ Failure; LDH: Lactate Dehydrogenase; AST: Aspartate Transaminase; ALT: Alaniine Transaminase; UNL: Upper Normal Limit; Peric Effusion: Pericardial Effusion.

Patients	Study	Age	Disease	AAV Delivery Vector	Dose	Pre-Delivery Immunosuppression	Post-therapy immunosuppression	Pre-existing Conditions	Clinical Course	Echocardiogram	Explanation	Outcome
Case 1	Zaidman et al. 2023[28]	7	DMD	AAVrh74 + MHCK7 promoter	IV 1.3 x 10^{14} vg/kg	Corticosteroids	Corticosteroids (p.o. and I.V.)	Large deletion from exon 8 to 21 in dystrophion gene	3 weeks: severe diffuse muscular weakness. Myositis. ↑ TnnI, focal wall motion abnormalities decreased EF, ↑ T2 at Cardiac MR (CMR)	focal wall motion abnormalities decreased EF	Microdystrophin construct with hing 1 and the beginning of the spectrin like domain, absent in the patients and acting as 'non-self' epitope.	Resolved during follow-up
Case 2	Bonneman et al. 2023[26]	9	DMD	AAV-8	IV 1.3 x 10^{14} vg/kg	Corticosteroids	Corticosteroids (p.o. and I.V.)	Large deletion from exon 8 to 21 in dystrophion gene	3 weeks: severe diffuse muscular weakness. Myositis. ↑ TnnI, focal wall motion abnormalities decreased EF, ↑ T2 at Cardiac MR (CMR)	focal wall motion abnormalities decreased EF	Microdystrophin construct with hing 1 and the beginning of the spectrin like domain, absent in the patients and acting as 'non-self' epitope.	Resolved during follow-up
Case 3	Mendell et al. 2025[35]	-	DMD	rAAVrh74	IV 1.3 x 10^{14} vg/kg	Corticosteroids	Corticosteroids (p.o.)	-	Day 0: ↑ Tnn, vomiting, fever. Transient spontaneuous hypotension. No significant change in the echocardiogram.	No changes	Probable immune response of to the vecto caspid	Resolved during follow-up
Case 4	Lek et al. 2023[32]	27	DMD	rAAV-9 dSaCas9	IV 1 x 10^{14} vg/kg	Corticosteroids	Day 0: Corticosteroids<br>Day 1: Eculizumab<br>Day 6: Eculizumab, Tocilizumab, Anakinra	Lean muscle mass 45%<br>Restrictive Pulmonary Defect<br>Mild DMD Cardiomyopathy (EF 55%)	Day 1: PVCs, ↓ PLT<br>Day 5: ↓ EF (45-50%), ↑ TnnI, peric effusion. Day 6: ARDS ; Day 8: ECMO and death from MOF and hypoxic neurologic injury	Decreased EF (45-50%)	Cytokine-mediated Capillary Leak Syndrome due to treatment acute toxic effect	Death<br>(at autopsy severe DMD cardiomyopathy)
Case 5 - 7	Strauss et al. 2022[42]	6 m	SMA	scAAV-9-FL-SMNcDNA	IV 1.1 x 10^{14} vg/kg	Corticosteroids	Corticosteroids	-	Week 1: ↑ Tnn and Ck-MB	-	Probable immune response of to the vecto caspid	Resolved during follow-up
Case 8 to 36	Gowda et al. 2023[49]	6-10 m	SMA	scAAV-9-FL-SMNcDNA	IV 1.1 x 10^{14} vg/kg	Corticosteroids	Corticosteroids	-	Week 2: ↑ Tnn	Normal	Probable immune response of to the vecto caspid	Resolved during follow-up
Case 37 to 54	Servais et al. 2024[50]	4-14 m	SMA	scAAV-9-FL-SMNcDNA	IV 1.1 x 10^{14} vg/kg	Corticosteroids	Corticosteroids	-	Week 2: ↑ Tnn	Normal	Probable immune response of to the vecto caspid	Resolved during follow-up
Case 55 - 56	Weib et al. 2022[51]	12-16 m	SMA	scAAV-9-FL-SMNcDNA	1.1 x 10^{14} vg/kg	Corticosteroids	Corticosteroids	-	Week 1: ↑ Tnn	Normal	Probable immune response of to the vecto caspid	Resolved during follow-up
Case 57	Bitetti et al. 2023[52]	12-16 m	SMA	scAAV-9-FL-SMNcDNA	IV 1.1 x 10^{14} vg/kg	Corticosteroids	Corticosteroids	-	Week 2: ↑ Tnn	Normal	Probable immune response of to the vecto caspid	Resolved during follow-up
Case 58 to 67	Tokaty et al. 2023[59]	6 m -18 m	SMA	scAAV-9-FL-SMNcDNA	IV 1.1 x 10^{14} vg/kg	Corticosteroids	Corticosteroids	-	Week 2: ↑ Tnn	Normal	Probable immune response of to the vecto caspid	Resolved during follow-up
Case 68 - 69	Stettner et al. 2023[60]	6 m	SMA	scAAV-9-FL-SMNcDNA	IV 1.1 x 10^{14} vg/kg	Corticosteroids	Corticosteroids	-	Week 2: ↑ Tnn	Normal	Probable immune response of to the vecto caspid	Resolved during follow-up
Case 70 - 71	Shieh et al. 2023[65]		X-Lynk Myotub Myop	AAV-8	IV 3.5 x 10^{14} vg/kg	Corticosteroids	Corticosteroids + Sirolimus + Mycophenolate Mofetil	-	Within week 3: ↑ Tnn	Normal	Probable immune response of to the vecto caspid	Resolved during follow-up

Table S9. Characteristics of the 6 death cases related to immune activation after gene replacement therapy.

Abbreviations: AAV: Adenovirus; DMD: Duchenne Muscolar Dystrophy; EF: Ejection Fraction; PVCs: Premature Ventricular Contractions; PLT: Platelets; ARDS: Acute Respiratory Distress Syndrome; ECMO: Extracorporeal Membrane Oxygenation; MOF: Multi Organ Failure; LDH: Lactate Dehydrogenase; AST: Aspartate Transaminase; ALT: Alaniine Transaminase; UNL: Upper Normal Limit

Patients	Study	Age	Disease	AAV Delivery Vector	Dose	Pre-Delivery Immunosuppression	Post-therapy immunosuppression	Pre-existing Conditions	Clinical Course	Cause of death
Case 1	Lek et al. 2023[32]	27	DMD	rAAV-9 dSaCas9	IV 1 x 10^{14} vg/kg	Corticosteroids	Day 0: Corticosteroids<br>Day 1: Eculizumab<br>Day 6: Eculizumab, Tocilizumab, Anakinra	Lean muscle mass 45%<br>Restrictive Pulmonary Defect<br>Compensated Cardiomyopathy (EF 55%)	Day 1: PVCs, ↓ PLT<br>Day 5: Decreased EF (45-50%), ↑ TnnI, pericardial effusion.<br>Day 6: ARDS<br>Day 8: ECMO and death from MOF and hypoxic neurologic injury	Cytokine-mediated Capillary Leak Syndrome with cardiac dysfunction due to treatment acute toxic effect<br>(at autopsy presence of severe DMD cardiomyopathy without evidence of active inflammation)
Case 2	Guillou et al. 2022[46]	4 m	SMA	scAAV9	IV 1.1 x 10^{14} vg/kg	Corticosteroids	Corticosteroids<br>Day 12: Eculizumab	VUS in Complement Factor 1 Gene	Day 8: Vomiting, ↓ PLT, ↑ LDH, ↑ AST/ALT<br>Day 12: Acute Renal Failure and Haemolytic Anemia<br>Day 30: Cardiac Arrest	MOF with severe dysautonomia, hypovolemia, sepsis in a context of TMA
Case 3	Shieh et al. 2023[65]	5.6	X-Lynked Myotubular Myopathy	AAV-8	IV 1.3 x 10^{14} vg/kg	Corticosteroids	Corticosteroids, Anakinra, Tocilizumab, Ruxolitinib	pre-existing hepatobiliary vulnerability<br>Absence of liver peliosis	Within 1-4 weeks:<br>Hepatopathy, Severe Immune dysfunction, Ascites, Cholestatic Liver Failure with AST and ALT > 5 UNL	Sepis in a context of liver failure
Case 4	Shieh et al. 2023[65]	4.8	X-Lynked Myotubular Myopathy	AAV-8	IV 3.5 x 10^{14} vg/kg	Corticosteroids	Corticosteroids, Anakinra, Tocilizumab, Ruxolitinib	pre-existing hepatobiliary vulnerability<br>Absence of liver peliosis	Within 1-4 weeks:<br>Hepatopathy, Severe Immune dysfunction, Ascites, Cholestatic Liver Failure with AST and ALT > 5 UNL	Psudomonas Sepsis in a context of liver failure
Case 5	Shieh et al. 2023[65]	6.1	X-Lynked Myotubular Myopathy	AAV-8	IV 3.5 x 10^{14} vg/kg	Corticosteroids	Corticosteroids, Anakinra, Tocilizumab, Ruxolitinib	pre-existing hepatobiliary vulnerability<br>Absence of liver peliosis	Within 1-4 weeks:<br>Hepatopathy, Severe Immune dysfunction, Ascites, Cholestatic Liver Failure with AST and ALT > 5 UNL	Circulatory Collapse due to Gastrointestinal Bleeding in a context of liver failure
Case 6	Shieh et al. 2023[65]	2.5	X-Lynked Myotubular Myopathy	AAV-8	IV 3.5 x 10^{14} vg/kg	Corticosteroids	Corticosteroids, Anakinra, Tocilizumab, Ruxolitinib	pre-existing hepatobiliary vulnerability<br>Absence of liver peliosis	Within 1-4 weeks:<br>Hepatopathy, Severe Immune dysfunction, Ascites, Cholestatic Liver Failure with AST and ALT > 5 UNL	Septic Shock in a context of liver failure

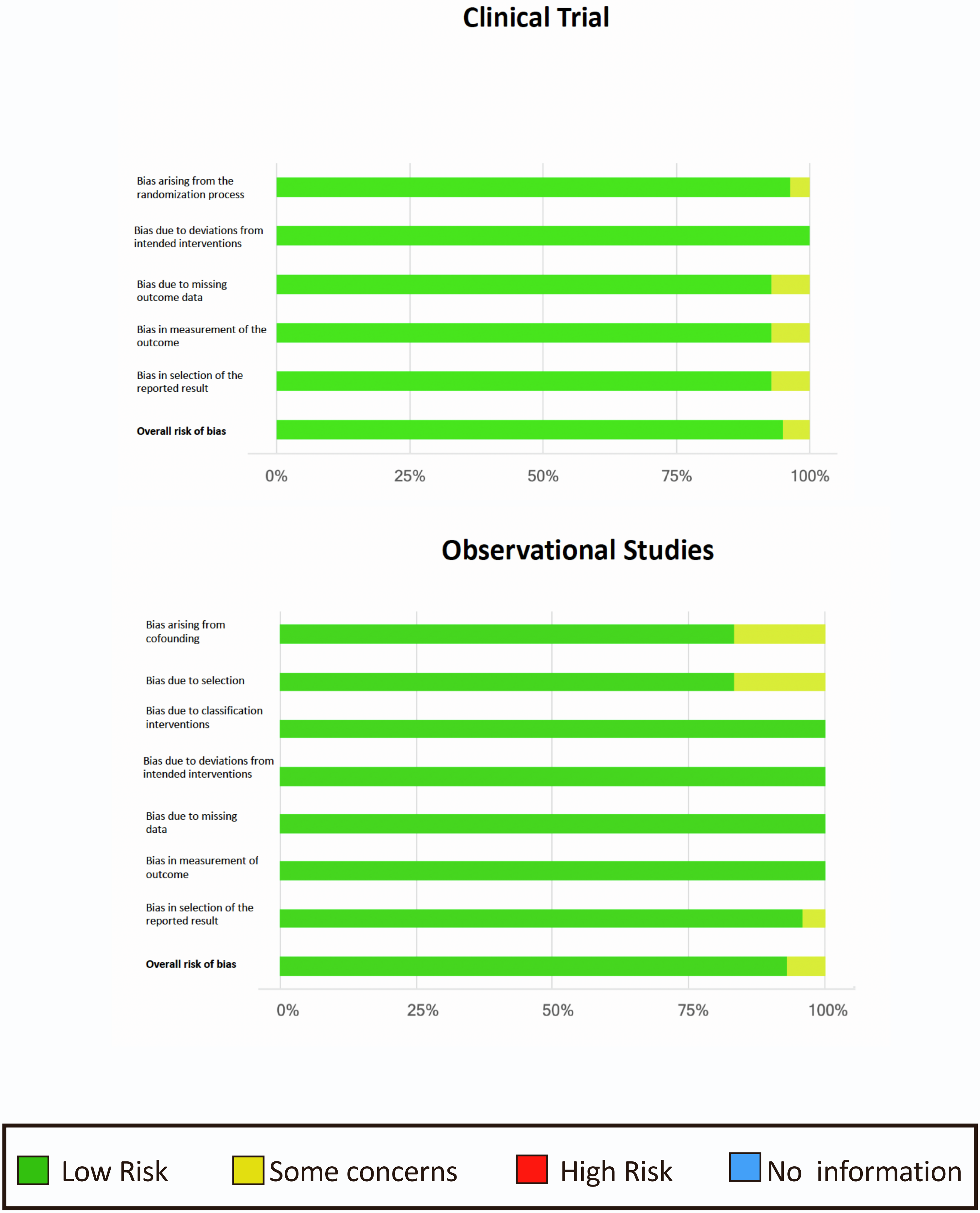

Figure S1. Synthesis of eligible clinical trial and of observational studies of risk of bias using the Cochrane risk-of-bias tool for randomized trials (RoB 1.0 and 2.0).

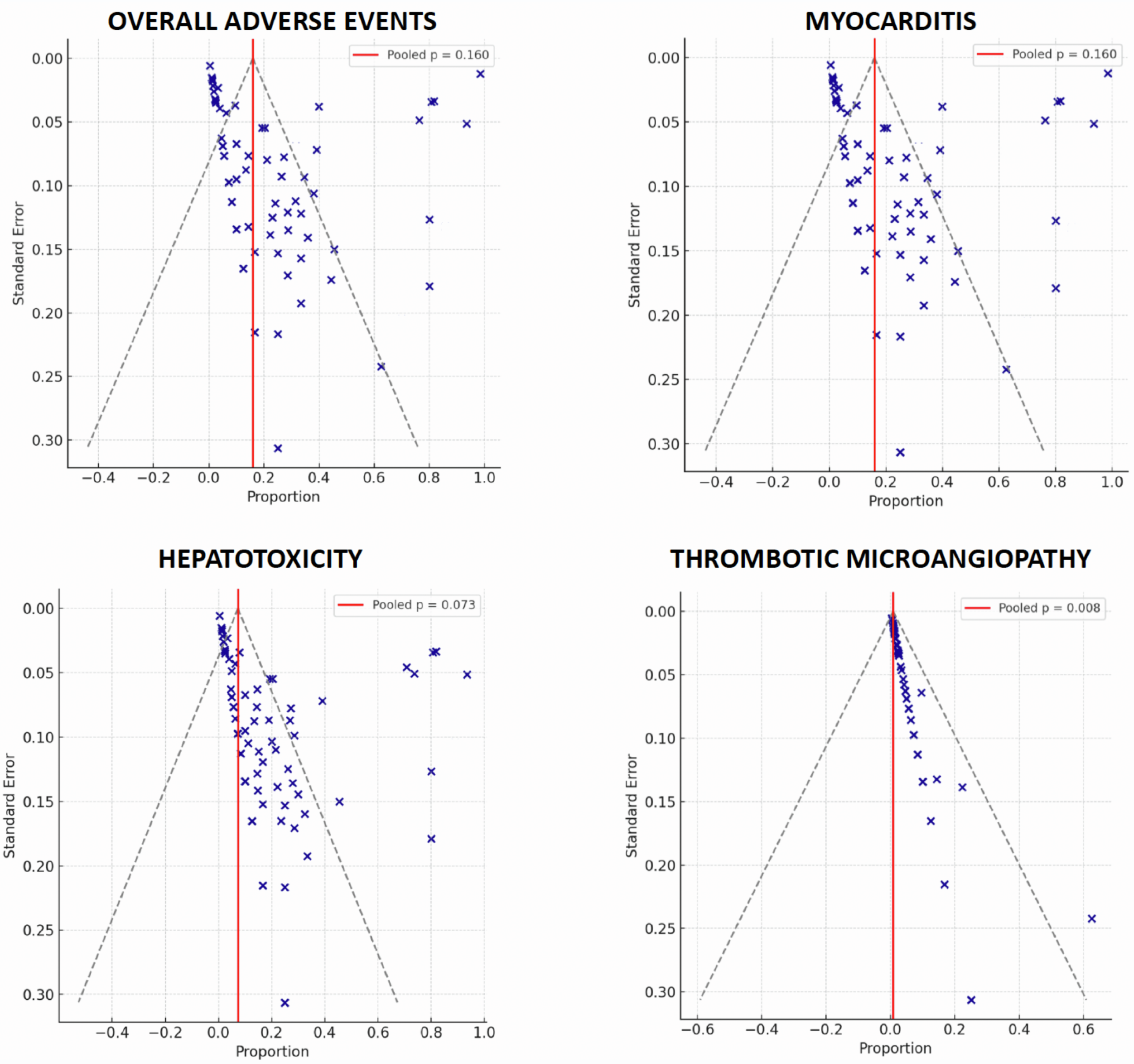

Figure S2 Funnel Plots for visual assessement of publication bias for the pooled incidence of Overall immune mediated adverse events (Top Left), Myocarditis (Top Right), Hepatotoxicity (Bottom Left) and Thrombotic Microangiopathy (Bottom Right).

Study	Year	Adverse Events	Patients	Incidence, %	95% CI, %	W, % (random)
Greenber et al.	2024	1	7	14.2	[2.5-51]	1.2
Hughes et al.	2022	0	6	0	[0-39]	1.1
Nakamura et al.	2023	0	32	0	[0-9.5]	1.3
Mendell et al.	2023	1	4	23	[12.4-35.7]	1
Zaidman et al.	2023	2	20	10.2	[2.8-30.1]	1.8
Belluscio et al.	2023	3	19	15.7	[2.7-64.3]	2.2
Dreghici et al.	2022	4	9	33.3	[12.1-64.5]	2.3
Laugel et al.	2024	1	3	33.3	[14.2-67.2]	1.2
Lek et al.	2023	1	1	100.0	[20.1-100]	0.1
Mendell et al.	2025	6	63	9.5	[4.4-19.2]	2.6
Flaning et al.	2022	0	2	0	[0-45.3]	1.1
Bowles et al.	2012	0	6	0	[0-42.1]	1.9
Mendell et al.	2010	0	6	0	[0-43.4]	1.9
Mendell et al.	2024	1	6	16.7	[3.5-56.5]	1.7
Smith et al.	2023	0	3	0	[0-46.3]	1.2
Smith et al.	2013	0	5	0	[0-44.1]	1.2
Corti et al.	2018	0	9	0	[0-38.4]	1.2
Strauss et al.	2022	5	15	33.3	[15.2-58.3]	2.5
Strauss et al.	2022	4	14	28.6	[11.8-54.7]	2.4
Mendell et al.	2017	2	15	13.3	[3.8-37.9]	2.2
Day et al.	2021	2	20	10	[2.8-30.1]	2.3
Merci et al.	2021	9	33	27.3	[15.1-44.2]	2.1
Goilou et al.	2022	1	1	100	[20.1-100]	1.1
Chand et al.	2021	3	3	100	[43-100]	1.2
Finekel et al.	2023	2	32	6.25	[1.7-20.1]	2.3
Gowda et al.	2022	99	99	100	[65-4-100]	1.3
Servais et al.	2024	67	168	39.8	[31.1-52.4]	2.1
Weib et al.	2022	58	76	76.7	[65.6-84.4]	2.2
Bitetti et al.	2023	6	9	66.7	[35.6-87.9]	2.1
Mendell et al.	2021	0	10	0	[0-38]	2.1
Favia et al.	2024	0	8	0	[0-32]	2.1
Waldrop et al.	2024	18	46	39.1	[26.4-53.5]	1.2
Waldrop et al.	2020	3	21	14.3	[4.9-34.6]	1.2
Chencheri et al.	2023	1	25	4	[0.7-19.5]	1.1
Pane et al.	2023	0	46	0	[0-19.4]	1.2
Tokaltly et al.	2023	25	25	100	[65.4-100]	3.1
Stettner et al.	2023	6	9	66.7	[35.4-87.9]	2.3
Matesanz et al.	2021	6	7	85.7	[48.7-97.4]	1.9
Gaber et al.	2021	7	9	77.8	[45.2-93.7]	2.2
D'Silva et al.	2022	8	21	38.1	[20.1-50.1]	2.1
Friese et al.	2021	2	9	25	[7.1-59.1]	2.1
Shieh et al.	2023	9	26	33.3	[18.6-52.1]	2.1
Ozelo et al.	2022	108	134	80	[72.5-85.1]	2.2
Ozelo et al.	2025	22	22	100	[51.4-100]	1.2
Manno et al.	2006	2	7	28.6	[28.2-61.1]	2.1
Pipe et al.	2023	11	54	20.3	[11.7-32.9]	2.1
Rangarajan et al.	2017	8	9	88.9	[56.5-98]	1.7
Mahlangu et al.	2024	108	132	80	[72.5-85.9]	1.4
Leavitt et al.	2024	5	11	45.5	[21.3-71.2]	1.3
Chapint et al.	2017	4	4	100	[65.3-100]	1.5
George et al.	2017	1	10	10	[1.8-40.4]	2.1
George et al.	2021	7	18	38.8	[23.4-56.7]	1.2
Nathwaani et al.	2011	2	6	33.3	[9.7-70]	1.2
Chowdary et al.	2022	8	10	80	[49.1-94.3]	1.2
Coppens et al.	2024	17	54	31.5	[23.2-51.3]	1.2
Cuker et al.	2024	28	45	62.2	[47.6-74.9]	1.2
Tai et al.	2022	0	26	0	[0-12.8]	1.2
Lv et al.	2024	0	6	0	[0-39.9]	1.2
Sevignz et al.	2024	0	19	0	[0-16.8]	1.2
D'Antigo et al.	2023	4	5	80	[47.5-96.3]	1.5
Tardieu et al.	2014	0	4	0	[9-48.9]	1.2
Tardieu et al.	2017	0	4	0	[0-48.9]	1.2
Deiva et al.	2021	0	4	0	[0-48.9]	1.2
Brunetti-Pierri et al.	2022	2	9	22.2	[6.3-54.7]	1.9
Mendell et al.	2015	0	6	0	[0-39.9]	1.2
Lyon et al.	2020	0	5	0	[0-25]	0.8
Hammond et al.	2016	0	42	0	[0-7.5]	0.8
Jasky et al.	2009	0	9	0	[0-21.3]	0.8
Jessup et al.	2011	0	39	0	[0-13.4]	0.8
Greenber et al.	2016	0	123	0	[0-6.7]	0.8
Grines et al.	2002	2	60	3.3	[0.9-11.4]	0.8
Grines et al.	2003	10	52	19.2	[10.8-31.9]	0.8
D'Avola et al.	2016	0	8	0	[0-16.6]	0.8
Ferreira et al.	2014	0	5	0	[0-25.5]	0.8
Weinstein et al.	2025	12	12	100	[67.8-100]	0.8
Flotte et al.	2022	0	2	0	[0-47.8]	0.8
Priddy et al.	2019	0	21	0	[0-6.8]	0.7
Flotte et al.	2011	0	9	0	[0-13.3]	0.7
		**Random Effects Model**	**1 908**	**30**	**[22.5-38.9]**	**100**
	Heteogeneity: I²= 83.1	τ^2 = 1.69	p<0.001			

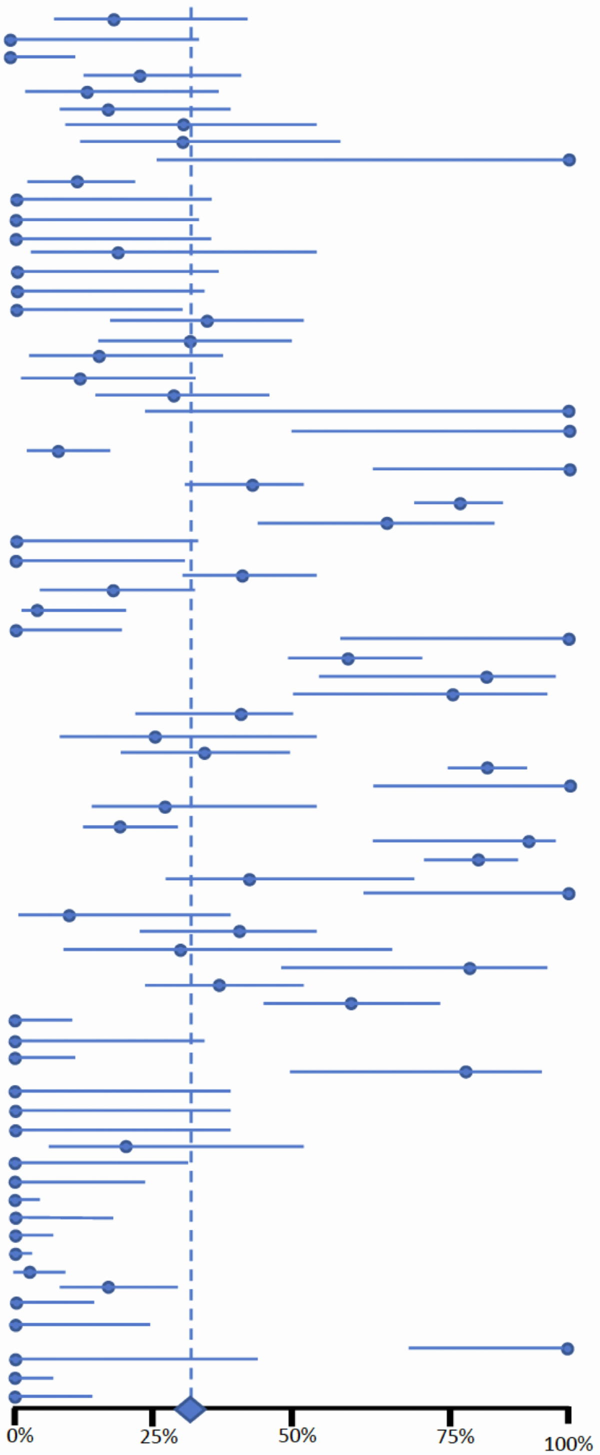

Figure S3 Forest Plot of incidence of immune mediated adverse events in AAV gene replacement therapies studies

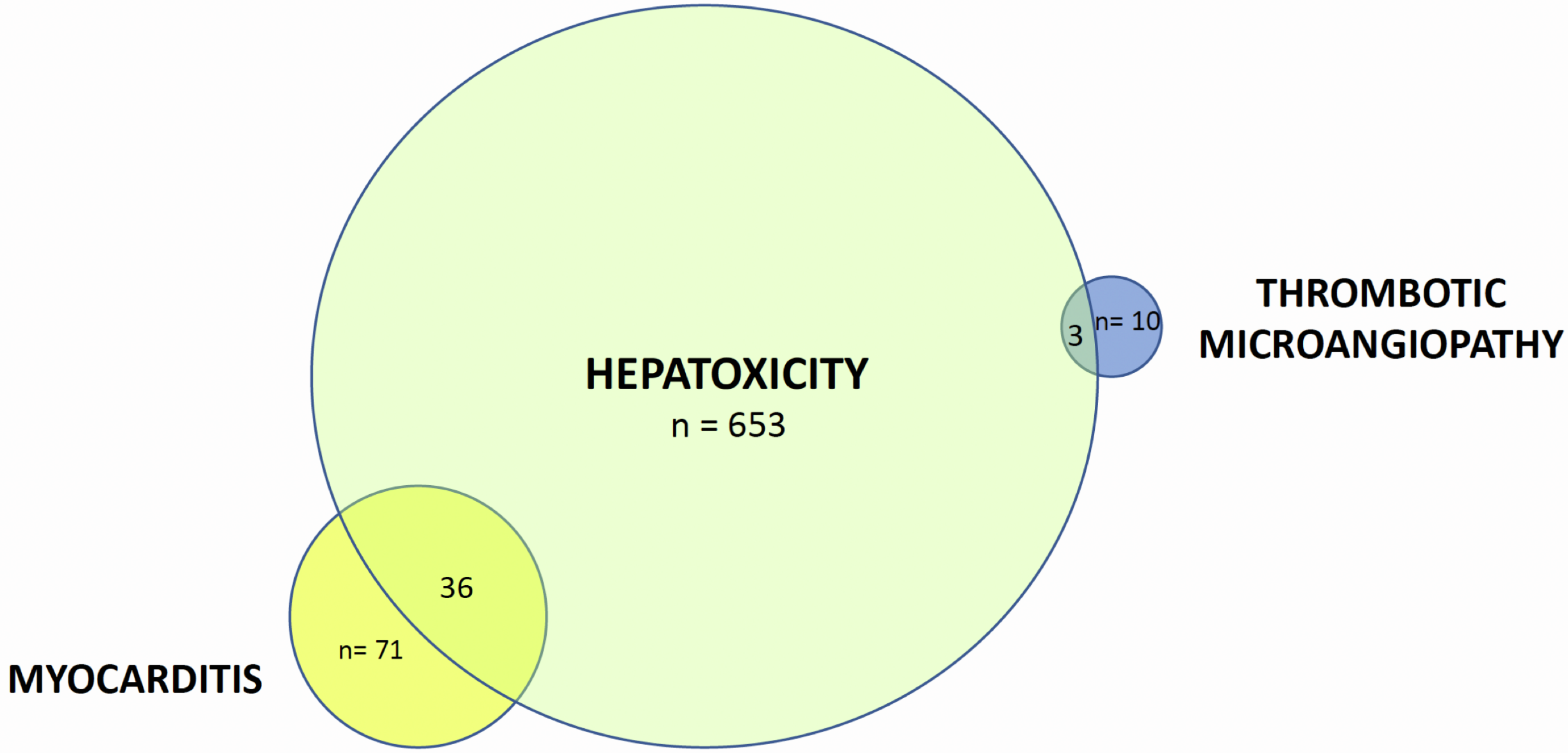

Figure S4 Overall occurrence and overlap of the 734 immune mediated AAV adverse events.

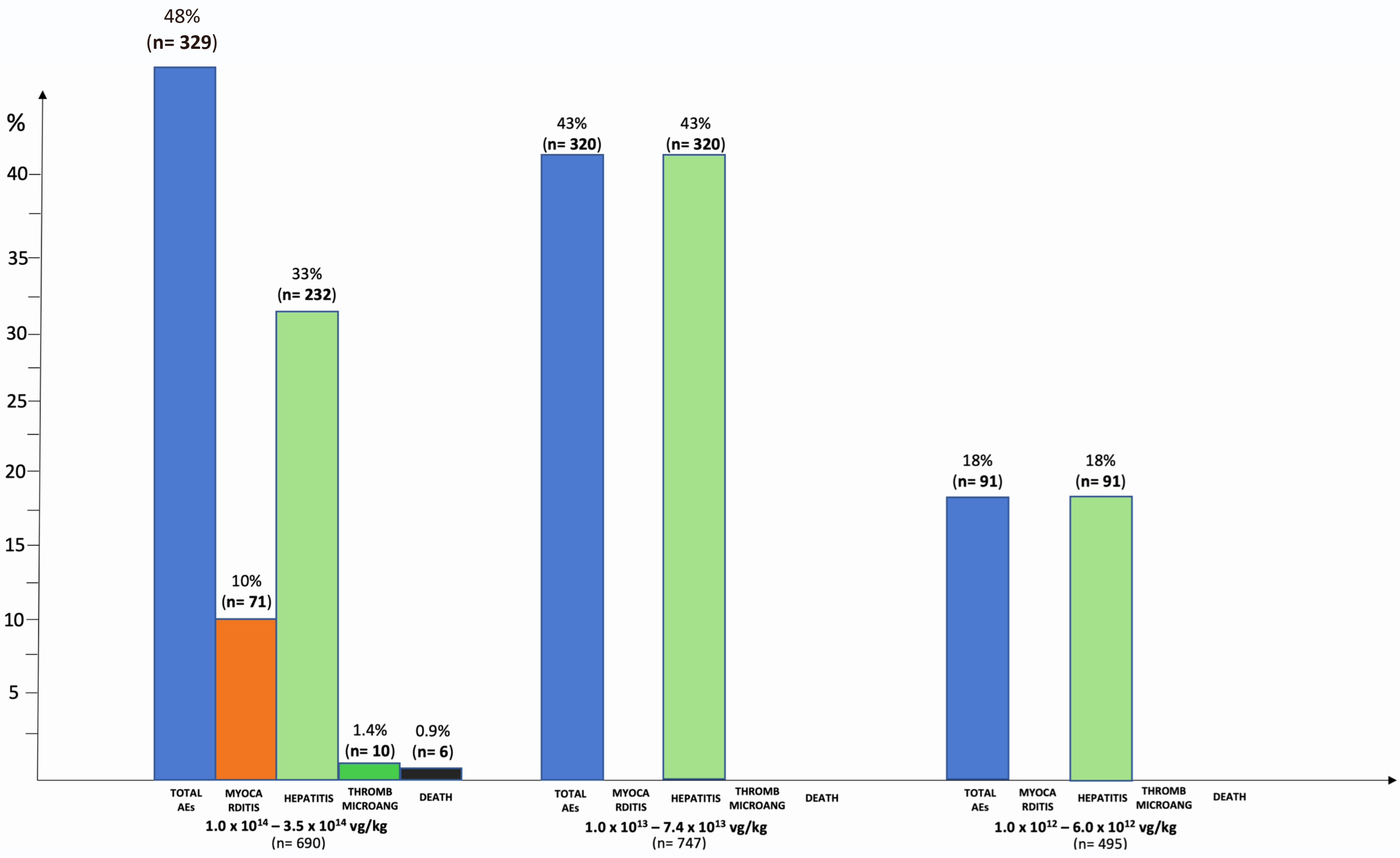

Figure S5 Prevalence of adverse events based on the infused dose.

Abbreviations: AEs: Adverse Events; THROM MICROANG: Thrombotic microangiopathy

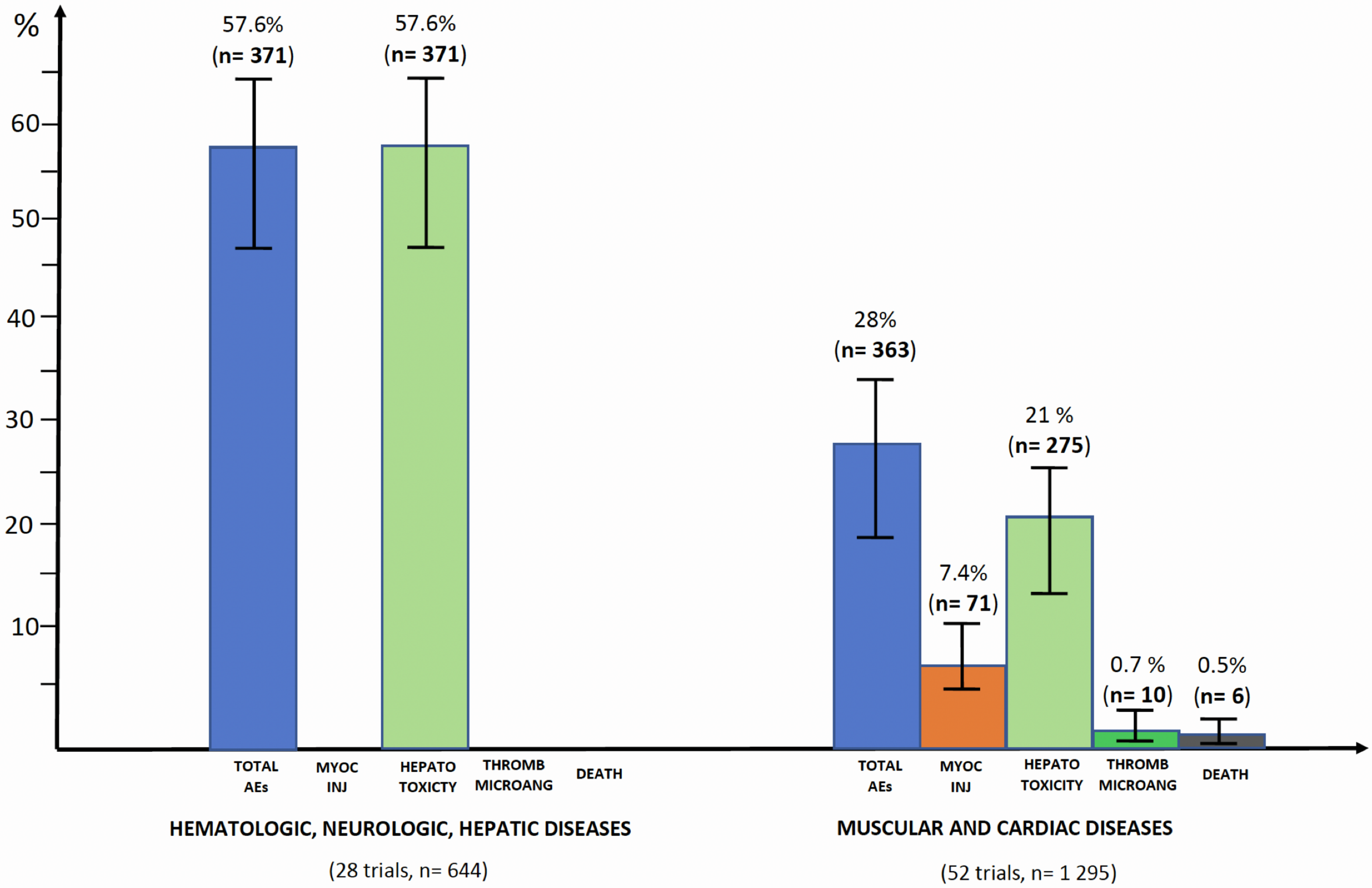

Figure S6 Prevalence of adverse events based on the type of treated disease.

Abbreviations: Aes: Adverse events; THROM MICROANG: Thrombotic microangiopathy
